# ﻿Three new species of torrent treefrogs (Anura, Hylidae) of the *Hyloscirtusbogotensis* group from the eastern Andean slopes and the biogeographic history of the genus

**DOI:** 10.3897/zookeys.1231.124926

**Published:** 2025-03-13

**Authors:** Andrea Varela-Jaramillo, Jeffrey W. Streicher, Pablo J. Venegas, Santiago R. Ron

**Affiliations:** 1 Museo de Zoología, Escuela de Biología, Facultad de Ciencias Exactas, Naturales y Ambientales, Pontificia Universidad Católica del Ecuador, Av. 12 de Octubre y Roca, Aptdo. 17-01-2184, Quito, Ecuador Pontificia Universidad Católica del Ecuador Quito Ecuador; 2 3Diversity, Santo Domingo Oe5-71 y Cuba, Quito, Ecuador 3Diversity Quito Ecuador; 3 Institute of Biology, Molecular Evolution and Systematics of Animals, University of Leipzig, Talstrasse 33, 04103 Leipzig, Germany University of Leipzig Leipzig Germany; 4 Herpetology, Natural History Museum, Cromwell Road, London, SW7 5BD, United Kingdom Natural History Museum London United Kingdom; 5 Rainforest Partnership, 4005 Guadalupe St., Austin, TX 78751, USA Rainforest Partnership Austin United States of America; 6 Instituto Peruano de Herpetología (IPH), Augusto Salazar Bondy 136, Urb. Higuereta, Surco, Lima, Peru Instituto Peruano de Herpetología (IPH) Lima Peru

**Keywords:** Amazon Basin, Andes, biogeography, Chocó, Peru, phenotypic plasticity, phylogenomics, phylogeny, speciation, ultra-conserved elements

## Abstract

The *Hyloscirtusbogotensis* group contains 17 species of treefrogs from the tropical Andes and Central America. A taxonomic review of the Amazonian clades of this group is presented based on DNA sequences of nuclear and mitochondrial DNA and a preliminary phylogenomic analysis of ultraconserved elements, as well as morphological, bioacoustic, and environmental characters. Additionally, the role of the Andes in the diversification of the genus *Hyloscirtus* is explored by reconstructing their ancestral basin (Amazon, Pacific, Caribbean). Our integrative analysis indicates the existence of eight undescribed candidate species within the group. Three of those species are described, previously masked within *H.albopunctulatus*, *H.phyllognathus*, and *H.torrenticola*. A lectotype is also designated for *Hylaalbopunctulata*. The new evidence suggests that neither *Hyloscirtusphyllognathus* nor *H.torrenticola* occur in Ecuador. The new species, *H.elbakyanae***sp. nov.**, *H.dispersus***sp. nov.**, and *Hyloscirtusmaycu***sp. nov.** differ from other members of the group in bioacoustics and external morphology. The most useful diagnostic characters among species were advertisement calls. In contrast, skin coloration is highly variable intraspecifically and, as a result, of low diagnostic value. High variation in color is partly a result of phenotypic plasticity. Our biogeographic reconstructions indicate that the Andean barrier influenced the diversification of *Hyloscirtus*. Since the early Oligocene, there have been only four colonization events across de Andes, between the Pacific and Amazon basins. Two of those events occurred more than 14 Mya, when most of the tropical Andes were below 3000 m. Species in the highland *H.larinopygion* group are younger, suggesting recent diversification as high montane forests and paramo habitats emerged.

## ﻿Introduction

There are more than 8800 amphibian species in the world (AmphibiaWeb 2024); however, many of them remain undescribed, especially in the tropics. Andean and Amazonian frog biodiversity remains severely underestimated due to cryptic diversity, where morphologically similar individuals actually represent multiple species (Funk et al. 2012). Several DNA-based studies using mitochondrial, nuclear, and genomic data have provided evidence that cryptic diversity is common in the Andes and Amazon region (Caminer et al. 2017; Nascimento et al. 2019; Páez and Ron 2019; Chasiluisa et al. 2020; Guillory et al. 2020) – proof that sustain scientists’ perseverance to unveil their real biodiversity.

The Andean genus *Hyloscirtus* Peters, 1882 (Hylidae, tribe Cophomantini) originated ~ 30–40 Ma (Wiens et al. 2006; Duellman et al. 2016; Feng et al. 2017) and is distributed in the Andes of Venezuela, Colombia, Ecuador, Peru, and Bolivia with a few species reaching lowland rainforests in Central America, the Chocó region, and the Amazon basin (Rojas-Runjaic et al. 2018; Yánez-Muñoz et al. 2021). Within *Hyloscirtus*, the *H.bogotensis* group currently contains 17 species that live associated with streams and riverine habitats, between 100–3600 m (Frost 2023; IUCN 2023). The monophyly of the group is mainly supported by molecular data, with 95 transformations in nuclear and mitochondrial proteins and ribosomal genes (Faivovich et al. 2005; Wiens et al. 2010). A mental gland, proposed to be involved in chemical communication during reproduction, is present in this group (Duellman 1972; Duellman et al. 1997); however, it is convergent in species of the *Hyloscirtusarmatus* species group and other species of the tribe Cophomantini (Brunetti et al. 2015).

Amazonian members of the *Hyloscirtusbogotensis* species group have several unresolved taxonomic issues (Almendáriz et al. 2014; Villacampa-Ortega et al. 2017). Within this group, *Hyloscirtusphyllognathus* Melin, 1941, *H.albopunctulatus* Boulenger, 1882 and *H.torrenticola* Duellman & Altig, 1978 occur in the Amazon basin of Colombia, Ecuador, and Peru, between 410–2190 m (Frost 2023; Suppl. material 1: fig. S1). *Hyloscirtusphyllognathus* inhabits from the northern Amazonian slopes of Cordillera Oriental of Colombia, continuing throughout the eastern Andes of Ecuador towards the southeast Andes of Peru. The type material was collected in Roque, San Martín Department, northern Peru (Melin 1941; Duellman 1972; Mueses-Cisneros 2005; Villacampa-Ortega et al. 2017). *Hyloscirtusalbopunctulatus* occurs in the Amazon region of Ecuador and Peru towards the southeastern tip of the Amazon in Colombia. The type material was collected in Sarayacu, Provincia Pastaza, Ecuador (Boulenger 1882; Lynch 2005). *Hyloscirtustorrenticola* is distributed in the southeastern slopes of the Oriental Andes of Colombia and the northeastern slopes of the Andes of Ecuador. The type material was collected near El Pepino, Putumayo Department, Colombia (Duellman and Altig 1978; Yánez-Muñoz and Reyes Puig 2008; Rivera-Correa 2016).

Many species of the *H.bogotensis* group have overlapping distribution ranges and are morphologically similar. As a result, their identification, based on morphological diagnosis from the literature, is problematic. For example, prior to Faivovich et al. (2006) taxonomic review, *H.albopunctulatus* was usually misidentified as *Boananympha* (e.g., Duellman and Mendelson 1995). The scarce information available for *H.torrenticola* mentions few morphological differences to *H.phyllognathus* and is based mainly on tadpole morphology. However, their songs are markedly different (Duellman and Altig 1978). Moreover, the phylogenetic affinities of these species are unknown, except for *H.phyllognathus* (Pyron and Wiens 2011; Almendáriz et al. 2014; Guayasamin et al. 2015; Duellman et al. 2016; Yánez-Muñoz et al. 2021). Almendáriz et al. (2014), based on genetic data, report that Peruvian and Ecuadorian populations of *H.phyllognathus* are likely separate species (see also Rivera-Correa 2016). Additionally, Villacampa-Ortega et al. (2017) suggest that populations from the south of Peru are a different species. Guayasamin et al. (2015) highlight the genetic separation between populations from northern and southern Ecuador, all identified as *H.phyllognathus*. Therefore, given the taxonomic problems among Amazonian members of the *H.bogotensis* group, a large-scale population sampling and an integrative approach are needed to determine their true species limits, phylogenetic relationships, and geographic distribution.

Most species in the *Hyloscirtusbogotensis* species group inhabit the northern Andes, one of the most species-rich regions on earth (Rahbek et al. 2019). The causes for the high species richness of the northern Andes, and tropical mountains in general, are still enigmatic, a knowledge gap known as Humboldt’s enigma (Rahbek et al. 2019). Biogeographic studies of densely sampled clades within Neotropical amphibians are scant (Crawford and Smith 2005; Wiens et al. 2006; Santos et al. 2009; Gonzalez-Voyer et al. 2011; Castroviejo-Fisher et al. 2014). Therefore, examining the diversification and biogeography of Andean clades, like *Hyloscirtus*, can help to understand why tropical mountains harbor so many species. Until now, the role of the Andes, as a barrier, in the diversification of *Hyloscirtus* has not been examined. In this paper, we investigate the phylogenetic relationships of treefrogs of the genus *Hyloscirtus* with emphasis on the Amazonian species of the *Hyloscirtusbogotensis* group. By using an integrative approach, we describe three new species and redefine the population content of *Hyloscirtusphyllognathus* and *H.albopunctulatus*. Additionally, we explore the ancestral geographic distribution and diversification history of the genus.

## ﻿Materials and methods

### ﻿Ethical statement

The procedures for handling specimens were conducted in full compliance with the guidelines for the management of live amphibians and reptiles in field and laboratory research (Beaupre et al. 2004; Esselstyn et al. 2008). Our research was conducted under collecting permits 001-11 IC-FAU-DNB/MA, 002-16 IC-FAU-DNB/MA, 003-17 IC-FAU-DNB/MA, 005-14 IC-FAU-DNB/MA, 005-2009-INVESTIGACIÓN-B-DPMS/MAE, 008-09 IC-FAU-DNB/MA, 011-2018-IC-FAU-DNB/MA, and 018-2016-IC-FLO-FAU-DPZCH-UPN-VS/M issued by the Ecuadorian Ministry of Environment.

### ﻿Molecular analyses

We extracted DNA from 93 samples of amphibian tissue (deposited at the QCAZ Museum at the Pontificia Universidad Católica del Ecuador) following standard phenol-chloroform extraction protocols (Sambrook et al. 1989). These samples belonged to individuals previously identified as *H.albopunctulatus* and *H.phyllognathus*, mainly based on geographic location and some coloration patterns (e.g., specimens with large white spots in the dorsum and thick white tarsal folds were identified as *H.albopunctulatus*). However, these identifications were considered tentative as it was clear that the published diagnostic characters to differentiate *H.phyllognathus* from *H.albopunctulatus* were unreliable among Ecuadorian populations (SRR pers. obs.)

For Sanger sequencing, we used a standard polymerase chain reaction (PCR) to amplify three mitochondrial genes (12S ribosomal subunit gene, 16S ribosomal subunit gene [final ~ 320 bp], and NADH dehydrogenase 1 [ND1] + adjacent tRNAs) and two nuclear genes (proto-oncogene cellular myelocytomatosis [c-myc] and recombination activating gene 1 [RAG-1]), using primers listed in Goebel et al. (1999), Faivovich et al. (2005), Wiens et al. (2005), Wiens and Moen (2008), and Zhang et al. (2013). We followed amplification protocols from Goebel et al. (1999). Amplicons were sequenced by the Macrogen® sequencing team (Seoul, Korea).

We assembled forward and reverse sequences and visually inspected the trimmed and edited sequences with Geneious 9.1.2 software (Gene Matters Corp; Kearse et al. 2012). 321 newly generated sequences, from 93 individuals from Ecuador and Perú (one individual from nearby the *H.phyllognathus* type locality), are available in GenBank under accession numbers shown in Suppl. material 1: table S1. Additionally, we included 51 GenBank sequences, belonging to 47 individuals (available at https://www.ncbi.nlm.nih.gov), from Panamá, Venezuela, Colombia, Ecuador, Peru, and Bolivia. These sequences corresponded to all species of *Hyloscirtus* and were published by Faivovich et al. (2004); Darst and Cannatella (2004); Faivovich et al. (2005); Wiens et al. (2005, 2006); Elmer and Cannatella (2008); Coloma et al. (2012); Almendáriz et al. (2014); Guayasamin et al. (2015), and Rojas-Runjaic et al. (2018). As outgroup, we used species of *Boana* and *Dendropsophus* (Caminer and Ron 2014; Caminer et al. 2017) (see Suppl. material 1: table S1).

A total of 137 individuals were included in the study. We aligned the sequences using MAFFT Multiple Alignment with the algorithm LINS-i (Katoh et al. 2002). We imported the alignments into Mesquite version 3.31 (Maddison and Maddison 2018) for final adjustments by hand. The final matrix had 3417 characters and 137 terminals and is available in Zenodo.org under DOI: 10.5281/zenodo.10733261.

We chose Maximum likelihood and Bayesian inference as optimality criteria because, unlike maximum parsimony, they allow the specification of models of substitution that capture well-known processes of DNA evolution (e.g., unequal rates among sites; Yang 2014). We used the software Partition Finder v. 2.1.1 (Lanfear et al. 2012) to identify the best partition strategy and best-fit model of nucleotide evolution for the dataset. The analysis was performed using the corrected Akaike Information Criterion (AICc). We also explored ModelFinder from IQ-Tree analysis. The preferred partitions found with their best substitution models for both searches are listed in Suppl. material 1: table S2.

We conducted Bayesian phylogenetic analyses in MrBayes 3.2.6 (Ronquist et al. 2012). The analysis involved four parallel runs of the Metropolis-coupled Monte Carlo Markov for 20 million generations. Each run had five chains with a temperature of 0.1 and was sampled every 1000 generations. Evolution models and partition strategies were applied according to the results from Partition Finder (see above). We used Tracer v. 1.6 (Rambaut et al. 2007) to measure the convergence of independent runs and considered reached when the average standard deviation of split frequencies was < 0.05 between runs and ESS values were higher than 200 for all parameters. For the consensus tree, we discarded 10% of the first trees as burn-in. We assessed the support in Bayesian trees using posterior probabilities derived from the final tree set. We carried out the Bayesian analyses at Cipres Science Gateway (available at https//www.phylo.org; Miller et al. 2010). We performed Maximum Likelihood analyses in IQ-Tree v. 1.6.8 (Nguyen et al. 2015; Trifinopoulos et al. 2016) using the partition schemes and best evolution models found by ModelFinder (see above) as implemented in IQ-Tree (TESTMERGE command; Kalyaanamoorthy et al. 2017) under the AICc criterion. A total of ten independent searches were run, looking for the best tree. Likelihood values of the searches were within 0.1 likelihood units, indicating convergence. We assessed the support using 100 bootstrap pseudoreplicates (-b 100 command).

We calculated genetic distances (uncorrected *p*-distances) using MEGA 5 (Tamura et al. 2011) for the 12S gene matrix; however, as the most widely used gene for species limits is 16S, we amplified 16S gene complete sequences (1388 kb) for the candidate species (see Suppl. material 1: table S3; Fouquet et al. 2007b).

Additionally, we sequenced nuclear ultra-conserved elements (UCEs) from a subset of 48 individuals (Suppl. material 1: table S1) to compare to the smaller (in terms of number of loci) mitochondrial and nuclear datasets. We followed a similar sequence capture protocol for anurans developed by Streicher et al. (2018), which uses the tetrapod probes of Faircloth et al. (2012) targeting 5060 UCE loci. We started with 200 ng/µL of double-stranded DNA (dsDNA) and built genomic shotgun libraries using New England Biolabs (NEB) reagents, including an initial fragmentation step with dsDNA fragmentase (NEB, M0348). Cleaning between all library construction steps (end-repair, a-tailing, ligation, size-selection, and enrichment) was performed with Serapure magnetic beads (Rohland and Reich 2012). We used the same custom adapters for the ligation step as Streicher et al. (2018). Size-selection of fragments between 200 and 500 base pairs was conducted using Blue Pippen (Sage Science) electrophoresis. The final PCR enrichment step included 12 cycles and libraries were amplified using TruSeq primers TS1 (5’-AGA TCG GAA GAG CAC ACG TCT GAA CTC CAG TCA C*AT CTC GTA TGC CGTC TTC TGC TTG-3’) and TS2 (5’-AGA TCG GAA GAG CGT CGT GTA GGG AAA GAG TGT AGA TCT CGG TGG TCG CCG TAT CATT-3’) and a Phusion enzyme master mix (NEB, M0531). We conducted the final enrichment PCR as 15 equal replicates that were later pooled to avoid PCR biases. We confirmed successful genomic library construction with a 2200 Agilent Tape Station.

After genomic library construction, we used the MYbaits protocol (now Arbor Biosciences) for targeted sequence capture. To hybridize UCE probes with targeted *Hyloscirtus* DNA, we used a temperature of 65 C for 24 hours. We used Dynabeads (M-270 Streptavidin, Life Technologies) to isolate the biotinylated UCE probes. A post-capture enrichment PCR of 18 cycles was performed prior to sequencing using the same reagents as in the shotgun library construction. We confirmed a successful capture library construction with a 2200 Agilent Tape Station. Sequencing of the final UCE capture library was conducted using an Illumina NextSeq 500 and a paired end 2 × 150 medium output kit at the NHMUK sequencing facility (London, UK).

We processed the UCE-capture data using the bioinformatics suite Phyluce v. 1.5.0 (Faircloth 2016). Briefly, this involved the removal of adapter contamination and low-quality bases with trimmomatic (Bolger et al. 2014), de novo assembly of contigs using Velvet 1.2.10 (Zerbino and Birney 2008), matching to UCE probe sequenced in Phyluce, and exportation of a concatenated alignment for phylogenomic analysis. We included previously generated UCE data (Streicher et al. 2018) for nine outgroup taxa, *Lepidobatrachus*, *Ceratophrys*, *Agalychnis*, *Phyllomedusa*, *Litoria*, *Boana*, *Dendropsophus*, *Hyla*, and *Scinax* (Suppl. material 1: table S1). To conduct phylogenomic analyses, we used RAxML 8.2.10 (Stamatakis 2014) using settings as described in Streicher et al. (2016). Given that allowing for missing data can improve phylogenetic support with UCEs (Streicher et al. 2016; Barrientos et al. 2021), we used a relatively high threshold for missing data (up to 80% missing data per taxon). We also ran a sensitivity test of missing data by inferring phylogenies for those samples with (1) > 200 UCEs enriched and (2) > 300 UCEs enriched. All bioinformatic analyses were conducted using the HPC server at NHMUK. The two phylogenomic data matrices generated are available on Zenodo.org under DOI: 10.5281/zenodo.14503491.

### ﻿Species limits and haplotype network

We used two criteria to determine the most likely number of species within the taxa of the *Hyloscirtusbogotensis* species group examined here, focusing on individuals assigned to *H.albopunctulatus* and *H.phyllognathus*, as described in Caminer and Ron (2020): (1) we applied a Poisson Tree Processes (PTP) model (Zhang et al. 2013) as implemented in the bPTP server (http://species.h-its.org/ptp/), and (2) we applied the Automatic Barcode Gap Discovery (ABGD) method (available at https://bioinfo.mnhn.fr/abi/public/abgd; Puillandre et al. 2012) using default parameters and the Kimura two-parameter distances. For both criteria, we used the available sequences for 12S. These analyses were also made for the 16S sequences to confirm the differentiation of our target species, but the results are based on the 12S matrix. To decrease the probability of committing a Type I error (i.e., incorrectly rejecting the null hypothesis of a group of individuals belonging to a single species), we chose the most conservative result between criteria 1 and 2; therefore, we adopted the result that found the lowest number of candidate species.

We also built a median joining network for the gene C-myc using the R package “pegas” (Paradis 2010). We interpreted the lack of haplotype sharing among candidate species as consistent with the hypothesis of their evolutionary independence. The aligned matrix for the haplotype network analysis is available in Zenodo.org under DOI 10.5281/zenodo.14935905

### ﻿Divergence times and ancestral area estimation

We used the software BEAST 2.6.6 (Bouckaert et al. 2019) to infer a time tree for *Hyloscirtus*. We analyzed the Sanger sequences matrix used for the ML phylogenetic searches, simplified into one terminal per species. The matrix was divided into five partitions under the models specified by PartitionFinder (see Results). We applied the relaxed exponential clock among branches and the rate for each branch was drawn independently from an underlying lognormal distribution with a Yule tree prior to speciation. Clock models and trees were linked among all partitions. We used two secondary calibrations based on node ages estimated by Hime et al. (2021). The first node was the most recent common ancestor of *Boana* and *Hyloscirtus* at 33.3 My (node 494 in Hime et al. 2021: fig. S2), and the most recent common ancestor of *Hyloscirtus* and *Dendropsophus* at 50.0 My (node 481). The primary calibration points used by Hime et al. (2021) fall outside Hylidae. Therefore, our estimates should be interpreted with caution. The calibration points had a lognormal distribution prior with a ± 1 standard deviation. Because the ML analysis does not estimate branch divergence dates, it is less parameterized and should provide a better estimate of tree topology. Therefore, we enforced two topological constraints in BEAST to match the topology of the ML tree. The topological constraints were: (1) the monophyly of the *Hyloscirtuslarinopygion* group, and (2) the monophyly of the *H.larinopygion* + *H.armatus* + *H.charazani* groups.

We conducted an ancestral area estimation aimed to explore the potential effect of the Andes as a vicariant barrier in the diversification of *Hyloscirtus*. The Andes separates two hydrographic basins, Amazon (east of the Andes) and Pacific (west of the Andes). We reconstructed the ancestral basin to determine the number of colonization events between basins. If the Andes are, in fact, a significant barrier, we expected a low number of events given that most species occur below 2000 m and, in the northern Andes, both basins have been separated by mountain passes higher than 2000 m for at least 20 Mya (Boschman 2021). Five species occurring in basins emptying in the Caribbean Sea were coded as “Caribbean” (*H.callipeza*, *H.jahni*, *H.japreria*, *H.lascinius*, and *H.platydactylus*). Ancestral basins were reconstructed as a discrete character with three states: (1) Pacific, (2) Amazon, and (3) Caribbean. For the reconstructions, we used “phytools” 2.0 R package (Revell 2024) under stochastic character mapping with three models: (1) Equal Rates, (2) Symmetric transition, and (3) All Rates Different (Harmon 2019). Central American species were coded as part of “Pacific” given the well-known biogeographic affinity between the Chocó and Central American amphibian communities (e.g., Lynch 1997; Ron 2000). Species distribution data was obtained from Anfibios del Ecuador (Ron et al. 2022), AmphibiaWeb (2023), and the IUCN Red List website (IUCN 2023).

### ﻿Morphology

We examined 87 alcohol-preserved specimens of the Amazonian species of the *Hyloscirtusbogotensis* group available from Museo de Zoología (**QCAZ**) at Pontificia Universidad Católica del Ecuador and División de Herpetología at Centro de Ornitología y Biodiversidad (**CORBIDI**) in Peru. We also examined four syntypes of *Hylaalbopunctulata* (syntypes BMNH 1880.12.5.159–162, 1880.12.5.230) deposited at the Natural History Museum, London (**NHMUK**). All type material of the new species is deposited at the QCAZ collection. Measured specimens are listed as Suppl. material 1: table S4 and belong to *H.albopunctulatus*, *H.phyllognathus* and the three new species described in this paper. We analyzed quantitative and qualitative morphological characters following the methodology and terminology described in Duellman (1970) and Duellman (2001). We measured ten characters using digital calipers (± 0.01 mm): (1) snout-vent length (SVL); (2) head length (HL); (3) head width (HW); (4) eye diameter (ED); (5) tympanum diameter (TD); (6) tibia length (TL); (7) femur length (FEL); (8) foot length (FL); (9) interorbital distance (IOD); and (10) internarial distance (InD). The webbing formula of hand and foot follows Savage and Heyer (1967) with modifications by Myers and Duellman (1982). Sex was determined by inspection of secondary sexual characters (e.g., mental gland, vocal slits, and vocal sac), and when in doubt, by gonadal inspection (presence of testes and ovaries/eggs). Raw morphometric data is available in Zenodo.org under doi: 10.5281/zenodo.14940291.

We also assessed 12 qualitative characters on preserved specimens, unless otherwise mentioned: (1) dorsal and ventral skin texture (smooth, finely granular or granular); (2) dorsal, ventral, and flank coloration; (3) snout (truncated to rounded) in dorsal and lateral views; (4) tympanum (conspicuous or inconspicuous); (5) mental gland (present or absent); (6) nuptial pads and projecting prepollex (present or absent); (7) ulnar, tarsal, and cloacal folds (conspicuous or inconspicuous); (8) calcar tubercle (absent or present), (9) pericloacal spots (ill-defined or well-defined); (10) ulnar and tarsal tubercles (present or absent); (11) subarticular and supernumerary tubercles (conspicuous or inconspicuous); and (12) webbing coloration in life. Coloration in life was obtained from digital photographs; for color terminology, we used the tool Name that Color (available in https://chir.ag/projects/name-that-color/).

We carried out a Principal Components Analysis (PCA) to quantitatively assess morphometric differences among species. To remove the effect of body size, the PCA was applied to the residuals of the linear regression between the SVL and the other morphometric variables. We applied a multivariate analysis of variance (MANOVA) to the residuals to test for morphometric differences between sexes for each species. Because we did not find significant differences, except for tibia length in *H.albopunctulatus*, we excluded this character and conducted the PCA combining data of both sexes – which benefited the analysis since our sample for females compared to males was small. We also excluded tympanum diameter because it was inconspicuous or difficult to measure in many specimens. We retained only components with eigenvalues > 1. Additionally, we ran pairwise comparisons of SVL measurements between species using Student t-tests. We used R statistical software for the analyses (RStudio Team 2023).

### ﻿Bioacoustics

Calls were recorded in the field with a Sennheiser K6 ME-67 directional microphone and an OlympusTM LS10 digital recorder. Calls are deposited in the audio archive of the Museo de Zoología QCAZ and are available through the Anfibios del Ecuador website (https://bioweb.bio/). We also analyzed calls available at The Cornell Lab of Ornithology website (https://www.macaulaylibrary.org/) of *Hyloscirtusphyllognathus* (eastern Ecuador) and *H.torrenticola* (type locality, southeastern Colombia: Putumayo Department, El Pepino, 1974), recorded by W. E. Duellman, M.S. Foster and R.W. McDiarmid. Additionally, we analyzed two calls of *Hyloscirtusphyllognathus* from Peru, CORBIDI 9590 from the Province of San Martín (geographically close to the type locality of the species) and CORBIDI 9976 (tissue QCAZ 60025) from the Province of Picota, recorded by PJV. The calls recorded by PJV were taken at 48 kHz, 24-bit, WAV format with a digital recorder Marantz PMD661MK2 and a unidirectional microphone Sennheiser ME64. We used the software Raven 1.5 (Charif et al. 2010) to analyze the calls. We obtained the measurements of spectral variables using a Fast Fourier Transformation (FFT) of 1024 points, a frequency resolution of 43.1 Hz, window type Hann and filter bandwidth of 248 Hz. We measured the temporal variables on the oscillogram and spectral variables on the power spectrum. We followed the call-centered approach (uninterrupted units as calls whenever they are separated by longer silence intervals) and terminology for call parameters described in Köhler et al. (2017). Measured call variables were: (1) call duration: time from the beginning to the end of the call; (2) rise time of the call: time from the beginning of the call to the point of its maximum amplitude; (3) inter-call interval: time between the end of one call and the beginning of the next call; (4) dominant frequency of the call: frequency with the most energy, measured along the entire call; (5) fundamental frequency of the call: frequency with the most energy of the first harmonic of the call; and (6) frequency bandwidth of the call: the higher frequency at any point of the call minus the lowest frequency at any point of the call. If available, up to five calls were analyzed per individual to calculate an individual average. We conducted a Principal Components Analysis (PCA) to assess call differentiation among species. We included all acoustic variables measured in the analysis. For the PCA, we retained only components with eigenvalues > 1. We ran pairwise comparisons using Student t-tests to compare call durations and dominant frequencies between species. We used R statistical software for the analyses (RStudio Team 2023). Raw call data is available in Zenodo.org under doi: 10.5281/zenodo.14940291.

### ﻿Environmental data and conservation assessments

We characterized environmental conditions for known localities of each species by measuring eight bioclimatic variables at each locality using layers obtained from WorldClim (30s resolution; Hijmans et al. 2005). The choice of the eight bioclimatic variables was based on Menéndez-Guerrero and Graham (2013): (1) annual mean temperature – BIO1; (2) mean diurnal range (Mean of monthly (max temp – min temp)) – BIO2; (3) temperature seasonality – BIO4; (4) maximum temperature of warmest month – BIO5; (5) minimum temperature of the coldest month – BIO6; (6) annual precipitation – BIO12; (7) precipitation of warmest quarter – BIO18, and (8) precipitation of coldest quarter – BIO19. We obtained locality data from the QCAZ collection database. We also included the type locality for *Hyloscirtustorrenticola* (10.3 km west, by road, of El Pepino, Departamento Putumayo, Colombia, 1,440 m, 01°11'N, 76°41'W; Duellman and Altig 1978) and for its Ecuadorian record (2 km SSW, by road, of the Río Reventador, Provincia Napo, Ecuador, 1490 m, 00°11'S, 77°39'W; Duellman and Altig 1978); the type locality for *H.albopunctulatus* (based on our lectotype designation: Sarayacu, Provincia Pastaza, Ecuador, 1243 m, 01°43'48"S, 77°29'24"W; Ron et al. 2022) and the type locality of *H.phyllognathus* (17 km NE Tarapoto, Departamento San Martín, Perú, 850 m, 06°21'16.56"S, 76°14'4"W, Wiens et al. 2006). Two localities close to *H.phyllognathus* type locality were included (Catarata Ahuashiyacu, Departamento San Martín, Perú, 730 m, 06° 30'0"S, 76°20'4.81"W and 35 km, road Tarapoto-Yurimaguas, Departamento San Martín, 594 m, Perú, 2°25'46.16"S, 76°16'3.65"W; CORBIDI). Additionally, we added another record of *H.phyllognathus* from Perú (Puesto de Control 15, Quebrada Mishquiyacu, Picota, Perú, 959 m, 6°56'26.81"S, 76°3'49.97"W; CORBIDI). We conducted a Principal Component Analysis (PCA) for all the variables and retained only components with eigenvalues > 1. We ran pairwise comparisons using Student t-tests to compare habitat preferences between species. We used R statistical software for the analyses (RStudio Team 2023). Raw bioclimatic data is available in Zenodo.org under doi: 10.5281/zenodo.14940291.

We also assessed the Red List status of each species according to the IUCN Red List criteria (IUCN 2001). Geographic ranges were estimated using minimum convex polygons in QGIS software version 3.12.

## ﻿Results

### ﻿Phylogenetic analyses and biogeographic history

**Figure 1.**
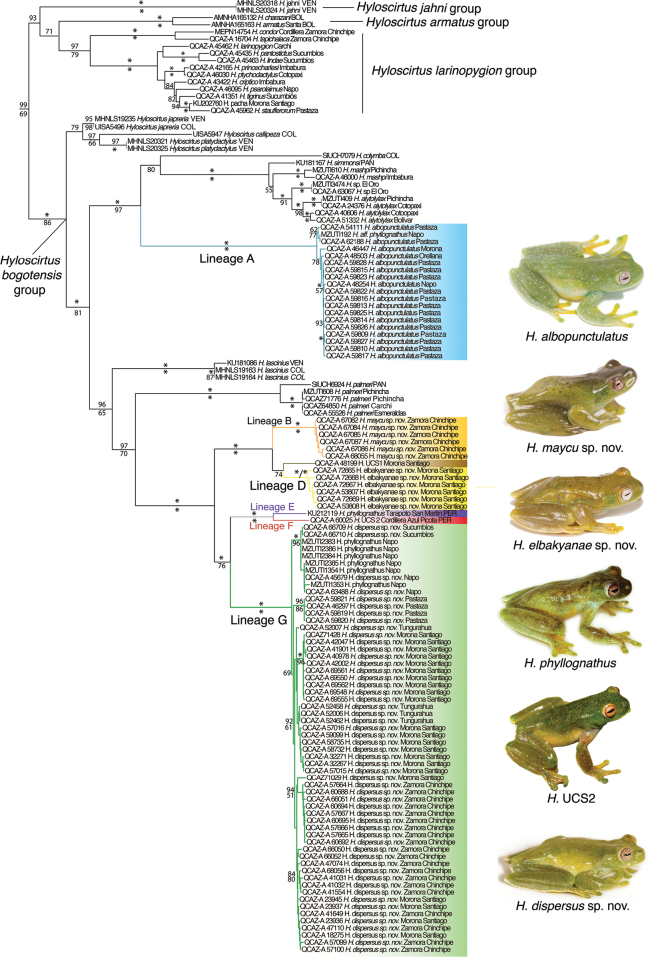
Maximum likelihood phylogram of *Hyloscirtus* for DNA sequences of mitochondrial (12S rRNA, 16S rRNA, ND1 and adjacent tRNAs) and nuclear genes (RAG1 and c-myc). Bayesian posterior probabilities (pp × 100) are shown above branches and bootstrap values below. Asterisks represent values of 100%. Missing values indicate posterior probabilities and bootstrap < 50. Amazonian species of the *H.bogotensis* group are shown with colored boxes. Outgroup species are not shown and include two species of *Boana* and two of *Dendropsophus* (Suppl. material 1: table S1). Voucher museum numbers are shown before the species name. For Ecuadorian populations, the province is provided after the species name. Abbreviations for other countries at the end of terminals: BOL (Bolivia), COL (Colombia), PAN (Panamá), PER (Perú), and VEN (Venezuela). UCS: unconfirmed candidate species. For locality data see Suppl. material 1: table S1.

**Figure 2.**
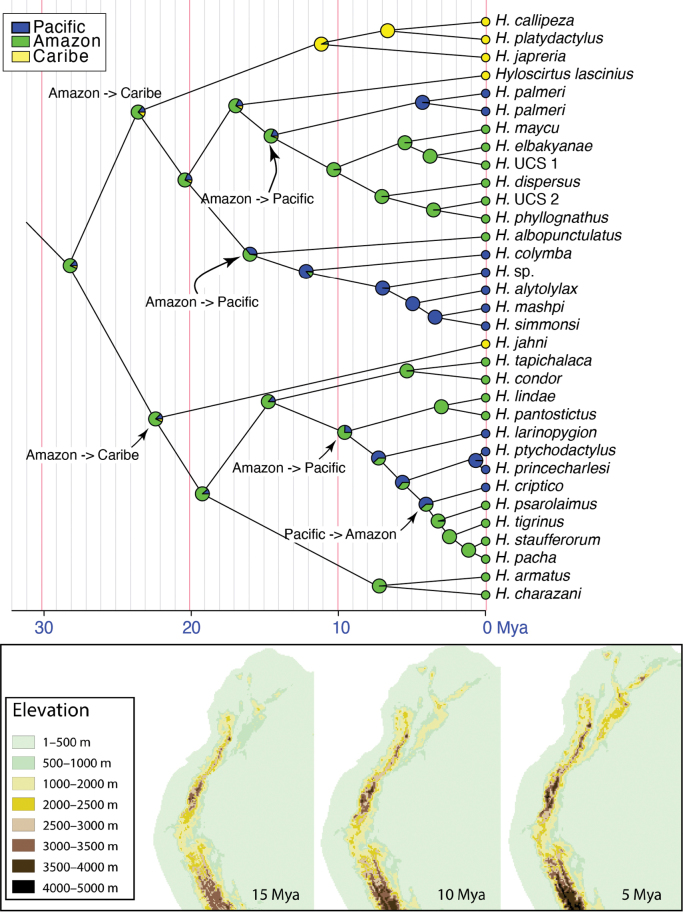
Time tree of *Hyloscirtus* species. Phylogeny based on Bayesian inferences with divergence time estimated in millions of years. One terminal per species is shown. Circles represent relative probabilities of ancestral distributions based on stochastic character mapping. Putative colonization events are shown with arrows. The maps below show an estimate of the elevation of the Andes, at the approximate times when trans-Andean colonizations took place (from Boschman 2021).

Maximum likelihood and Bayesian inference analyses resulted in similar topologies with incongruences only in clades with low support (Fig. 1). *Hyloscirtus* has two basally diverging clades, the *H.bogotensis* group and a clade including the *H.larinopygion*, *H.armatus*, and *H.jahni* species groups. Each species group had strong support in both analyses but weak support for relationships between groups, as previously reported (Coloma et al. 2012; Almendáriz et al. 2014; Guayasamin et al. 2015; Rojas-Runjaic et al. 2018; Ron et al. 2018). Amazonian members of the *Hyloscirtusbogotensis* group are part of two clades. One of them is composed predominantly by species from the Pacific basin and Central America, with a single Amazonian species (*H.albopunctulatus*). The other clade is composed predominantly by Amazonian species (Figs 1, 2), except for the basally diverging *H.lascinius* and *H.palmeri* (Caribbean, Pacific basin, and Central America).

**Figure 3.**
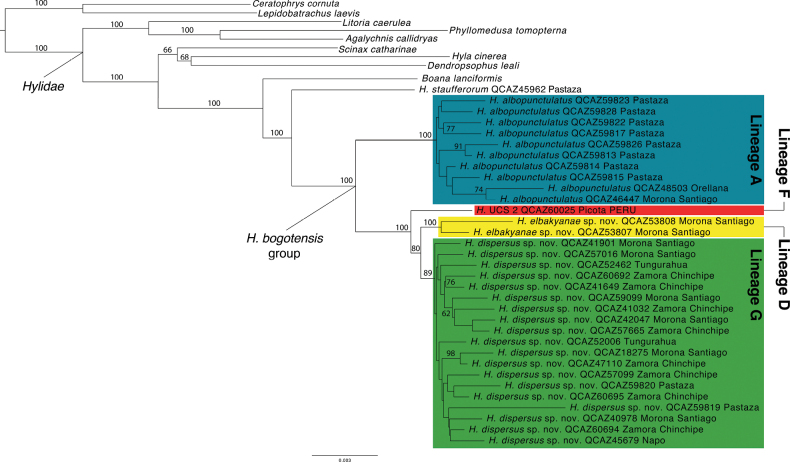
Best tree obtained under Maximum likelihood criterion when using taxa with more than 200 UCE loci enriched. Museum number of each individual is indicated. Numbers over the branches represent bootstrap values. Missing values indicate branch’s support below 60. All samples within the *H.bogotensis* group belong to Ecuador except for QCAZ 60025 (clade F) from Perú.

Compared to previous phylogenomic studies, the sequence capture obtained a low coverage of UCEs among *Hyloscirtus* samples with the number of UCEs captured ranging from 9 to 1773 and an average of 585 UCEs captured per individual (Table 1). This low coverage may be explained by laboratory issues during the capture step; however, due to funding constraints we were unable to repeat the sequencing experiment to determine the exact cause. Nonetheless, the average available UCE data still number in the hundreds per individual making them relevant to species delimitation. It is important to note that because we did not have complete sample overlap with the concatenated matrix from Sanger sequencing, some of the clades predicted by the species delimitation could not be evaluated. Eleven of the samples had fewer than 200 UCEs captured, so they were excluded from the phylogenomic analysis (Table 1). The > 200 UCEs per taxon dataset contained 37 ingroup taxa, 2125 UCEs and had a concatenated length of 479755 base pairs. The > 300 UCEs per taxon dataset contained 31 ingroup taxa, 2230 UCEs and a concatenated length of 515965. The maximum likelihood trees for these analyses are presented as Fig. 3 and Suppl. material 1: fig. S2. In both phylogenies, four taxa had unexpected phylogenetic placements when compared to the concatenated Sanger dataset (Fig. 1), including QCAZ 55766 (*H.dispersus* sp. nov.), QCAZ 55526 (*H.palmeri*), QCAZ 40606 (*H.alytolylax*), and QCAZ 51332 (*H.alytolylax*). These unexpected placements were all associated with poorly supported branches (< 64 bootstrap support in the > 200 UCEs analysis; < 75 bootstrap support in the > 300 UCEs analysis, Suppl. material 1: figs S3, S4). As such, we removed these four taxa and re-ran the likelihood analyses in RAxML. The revised datasets contained 2207 and 2327 UCEs, for the > 200 and > 300 sets, respectively. The revised phylogenies from these datasets are presented as Fig. 3 (> 200 UCEs per taxon) and Suppl. material 1: fig. S2 (> 300 UCEs per taxon). Removing the four taxa improved branch support deep in the tree (but not for all shallow branches). When compared to the Sanger mtDNA + nucDNA analysis (Fig. 1), the UCE-based phylogeny is broadly similar with strong support for the monophyly of *H.dispersus* sp. nov. (Lineage G), *H.elbakyanae* sp. nov. (Lineage D), and *H.albopunctulatus* (Lineage A), and the distinctiveness of Lineage F. *Hyloscirtusmaycu* sp. nov. (Lineage B) was not part of this analysis because the specimens and tissues were collected a year after the UCE sequencing was completed.

**Table 1.** Quality metrics for *Hyloscirtus* UCE samples in this study include number of contigs, number of UCEs enriched, and its coverage per sample. See Suppl. material 1: table S1 for the locations of the *Hyloscirtus* species. The first 10 rows belong to the outgroup used for this analysis. The asterisks (*) represent the samples included in the 200 UCEs phylogeny reconstructions after filtering for missing data. The sum symbol (+) are samples removed to reconstruct the phylogeny in Fig. 3 since they were problematic (see Results).

Species	Museum number	Contigs	UCEs	UCEs coverage (%)
*Dendropsophusleali*	SAMN 05559892	8555	1996	23.33
*Boanalanciformis*	SAMN 05559916	4949	1057	21.35
*Agalychniscallidryas*	SAMN 05559871	3105	1373	44.21
*Hylacinerea*	SAMN 05559910	764	126	16.49
*Phyllomedusatomopterna*	SAMN 05559926	564	134	23.75
*Litoriacaerulea*	SAMN 05559920	30,118	1898	6.30
*Scinaxcatharinae*	SAMN 05559930	3413	1273	37.29
*Lepidobatrachuslaevis*	SAMN 05559917	5814	2226	38.28
*Ceratophryscornuta*	SAMN 05559887	40,359	1967	4.87
*Boanafasciata*	QCAZ48583	63434	137	0.22
*H.alytolylax*	QCAZ40606^+^	2885	326	11.30
*H.alytolylax*	QCAZ51332^+^	10309	609	5.91
*H.criptico*	QCAZ43422	2101	157	7.47
*H.staufferorum*	QCAZ45962*	3180	231	7.26
*H.palmeri*	QCAZ55526^+^	7208	487	6.76
*H.albopunctulatus*	QCAZ46447*	34921	403	1.15
*H.albopunctulatus*	QCAZ48254	763	86	11.27
*H.albopunctulatus*	QCAZ48503*	2814	213	7.57
*H.albopunctulatus*	QCAZ59813*	10318	687	6.66
*H.albopunctulatus*	QCAZ59814*	3215	310	9.64
*H.albopunctulatus*	QCAZ59815*	11085	581	5.24
*H.albopunctulatus*	QCAZ59816	147	22	14.97
*H.albopunctulatus*	QCAZ59817*	7194	423	5.88
*H.albopunctulatus*	QCAZ59822*	28247	998	3.53
*H.albopunctulatus*	QCAZ59823*	3182	237	7.45
*H.albopunctulatus*	QCAZ59826*	5216	322	6.17
*H.albopunctulatus*	QCAZ59827	105	9	8.57
*H.albopunctulatus*	QCAZ59828*	20251	957	4.73
*H.elbakyanae* sp. nov.	QCAZ53807*	63934	1699	2.66
*H.elbakyanae* sp. nov.	QCAZ53808*	7758	500	6.44
*H.dispersus* sp. nov.	QCAZ18275*	10234	893	8.73
*H.dispersus* sp. nov.	QCAZ23937	140	17	12.14
*H.dispersus* sp. nov.	QCAZ23945	189	26	13.76
*H.dispersus* sp. nov.	QCAZ40978*	22659	1016	4.48
*H.dispersus* sp. nov.	QCAZ41032*	7430	542	7.29
*H.dispersus* sp. nov.	QCAZ41649*	22281	1058	4.75
*H.dispersus* sp. nov.	QCAZ41901*	73651	1773	2.41
*H.dispersus* sp. nov.	QCAZ41951	128	17	13.28
*H.dispersus* sp. nov.	QCAZ42047*	3407	289	8.48
*H.dispersus* sp. nov.	QCAZ45679*	11175	711	6.36
*H.dispersus* sp. nov.	QCAZ47110*	4643	553	11.91
*H.dispersus* sp. nov.	QCAZ52006*	54758	1619	2.96
*H.dispersus* sp. nov.	QCAZ52007	4	0	0
*H.dispersus* sp. nov.	QCAZ52458	1199	169	14.10
*H.dispersus* sp. nov.	QCAZ52462*	17787	936	5.26
*H.dispersus* sp. nov.	QCAZ57016*	17434	942	5.40
*H.dispersus* sp. nov.	QCAZ57099*	24843	968	3.90
*H.dispersus* sp. nov.	QCAZ57665*	7640	521	6.82
*H.dispersus* sp. nov.	QCAZ57666^+^	31536	1261	4.00
*H.dispersus* sp. nov.	QCAZ58735	253	43	17.00
*H.dispersus* sp. nov.	QCAZ59099*	4831	462	9.56
*H.dispersus* sp. nov.	QCAZ59819*	14063	281	2.00
*H.dispersus* sp. nov.	QCAZ59820*	2433	211	8.67
*H.dispersus* sp. nov.	QCAZ60692*	9358	621	6.64
*H.dispersus* sp. nov.	QCAZ60694*	49801	1554	3.12
*H.dispersus* sp. nov.	QCAZ60695*	22767	296	5.69
*H.* UCS 2	QCAZ60025*	17136	913	5.33

Ancestral area estimation shows that the genus *Hyloscirtus* originated in the Amazon basin (Fig. 2). Divergence between the *H.bogotensis* group and its sister clade took place 28.5 Mya (high posterior density interval [95% HPD] = 21.6–35.0), in the early Oligocene (Fig. 2, Table 2). Diversification within the *H.bogotensis* species group started 23.8 Mya (95% HDP = 16.6–30.8). Overall, species from the highland clade, the *H.larinopygion* species group, are younger than those of the mid-elevation-lowland clade, the *H.bogotensis* species group (Fig. 2). While in the *H.larinopygion* group, ¾ of the species are younger than 5 million years, in the *H.bogotensis* group, only 1/3 are. Two species pairs of the *H.larinopygion* group had recent speciation events, *H.pacha*-*H.staufferorum* (1.3 Mya) and *H.princecharlesi*-*H.ptychodactylus* (1.2 Mya). Within the *H.bogotensis* species group, the youngest species pair is *H.simmonsi*-*H.mashpi* (3.6 Mya).

**Table 2.** Node crown ages of representative *Hyloscirtus* clades in millions of years. For each node we show the estimated age with the 95% high posterior density interval (HDP).

Group/Clade/Species	Node age	95% HDP
	Age	
*H.armatus* group	7.45	1.44–14.97
*H.larinopygion* group	14.79	8.52–21.45
*H.bogotensis* group	23.84	16.64–30.80
(*H. colymba + H.alytolylax + H.mashpi + H.simmonsi*) + *H.albopunctulatus*	16.22	9.81–22.5
(*H.maycu* sp. nov. + *H.elbakyanae* sp. nov. + *H.phyllognathus + H.dispersus* sp. nov.) + *H.palmeri*	14.79	9.14–20.80
(*H.callipeza + H.platydactylus + H.japreria*)	11.61	2.67–22.17
*H.phyllognathus + H.dispersus* sp. nov.	7.12	3.05–11.99
*H.alytolylax + H.mashpi + H.simmonsi*	4.99	1.77–8.70
*H.palmeri*	4.63	0.7–10.01
*H.phyllognathus*	3.6	0.77–7.11

Colonization events across the Andes, between the Pacific and Amazon basin, were infrequent in *Hyloscirtus*. During the 28 My of history of the *H.bogotensis* species group, there were only two events. Both events took place within a short period in the Miocene (14.8 and 16.2 Mya) and both were from the Amazon to the Pacific (Fig. 2). The other colonization events occurred in the higher elevation *H.larinopygion* species group. One event took place nearly 10 Mya from the Amazon to the Pacific basin. The other event is the most recent, at 4.2 Mya, and is the only event from the Pacific to the Amazon. Two events from the Amazon to the Caribbean basin are the most ancient in *Hyloscirtus* and took place ~ 20 Mya. There were no colonization events from the Caribbean to other basins. Interestingly, *H.palmeri* populations from Ecuador and Panama diverged 4.5 Mya, prior to the closure of the Panama isthmus. The favored model for ancestral reconstruction was Equal Rates (AIC = 56.7) followed by Symmetric Transition (58.8) and All Rates Different (61.9). Genetic distance analysis found divergences ranging from 3.7–14.8% for 12S and 5.0–14.8% for 16S (Table 3).

**Table 3.** Pairwise genetic distances (uncorrected-*p*) between our target lineages of *Hyloscirtus. Hyloscirtusalbopunctulatus* (Lineage A), *H.maycu* sp. nov. (Lineage B), *H.* UCS 1 (Lineage C), *H.elbakyanae* sp. nov. (Lineage D), *H.phyllognathus* sensu stricto (Lineage E), *H.* UCS 2 (Lineage F), and *H.dispersus* sp. nov. (Lineage G), based on sequences of 12S (below the diagonal) and 16S (above the diagonal). Mean and ± standard deviations are given with range in parentheses. Sequences of 16S were not available for Lineage E.

Lineage	A	B	C	D	E	F	G
**A**	–	0.131	0.160	0.131	–	0.127	0.153 ± 0.001 (0.151–0.155)
**B**	0.143 ± 0.002 (0.138–0.146)	–	0.14.7	0.051	–	0.117	0.128 ± 0.001 (0.123–0.129)
**C**	0.139 ± 0.001 (0.136–0.14)	0.046 ± 0.002 (0.043–0.049)	–	0.050	–	0.110	0.101 ± 0.002 (0.100–0.106)
**D**	0.129 ± 0.001 (0.125–0.131)	0.037 ± 0.001 (0.035–0.041)	0.039 ± 0.001 (0.037–0.041)	–	–	0.100	0.099 ± 0.009 (0.097–0.105)
**E**	0.127 ± 0.001 (0.127–0.131)	0.082 ± 0.001 (0.078–0.082)	0.074	0.069 ± 0.001 (0.127–0.131)	–	–	–
**F**	0.138 ± 0.002 (0.136–0.140)	0.074 ± 0.001 (0.072–0.076)	0.076	0.062 ± 0.001 (0.062–0.064)	0.039	–	0.089 ± 0.001 (0.086–0.090)
**G**	0.141 ± 0.002 (0.131–0.148)	0.099 ± 0.003 (0.092–0.109)	0.086 ± 0.001 (0.082–0.092)	0.083 ± 0.002 (0.078–0.094)	0.091 ± 0.002 (0.088–0.097)	0.084 ± 0.002 (0.082–0.090)	0.006 ± 0.004 (0.00–0.018)

### ﻿Species limits

The species limits analyses support 19 candidate species within the *Hyloscirtusbogotensis* group according to the ABGD criteria and 21 candidate species according to the bPTP criteria (Suppl. material 1: figs S5, S6). Following our conservative rule, we propose 19 species for this group. Of these, 11 are named and eight are undescribed, representing a 72.7% increase in the number of species.

**Figure 4.**
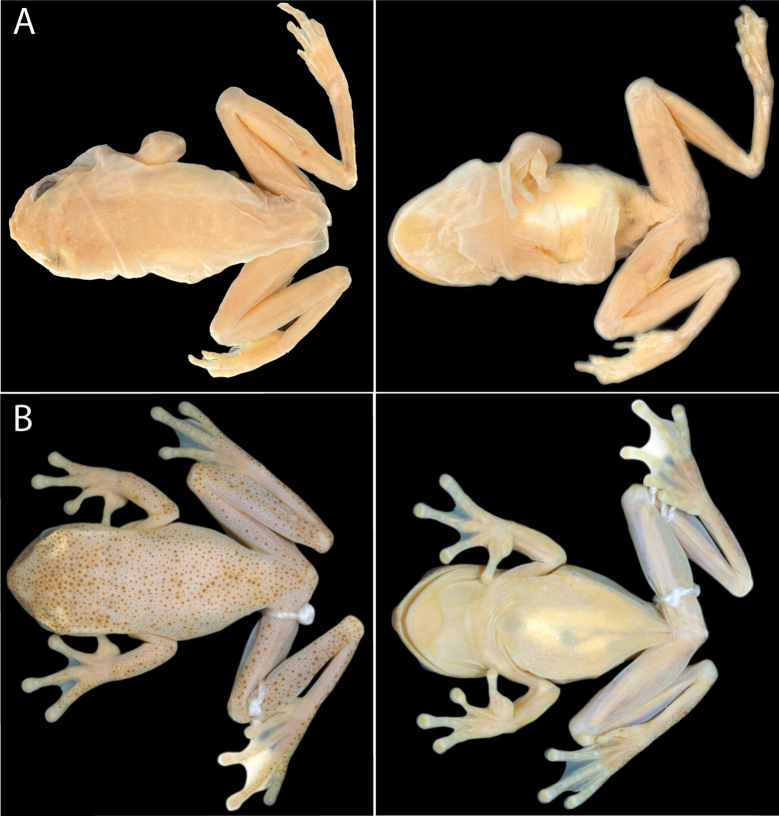
Photographs of the type material of *Hyloscirtusalbopunctulatus* and *H.torrenticola* species **A** dorsal and ventral views of the lectotype of *H.albopunctulatus* BMNH 1880.12.5.230 **B** dorsal and ventral views of the holotype of *H.torrenticola* KU 16957. Photographs by JWS (**A**) and Martín R. Bustamante (BIOWEB archive) (**B**).

The available names for target candidate species are *Hyloscirtusalbopunctulatus* (Boulenger, 1882), *Hyloscirtusphyllognathus* (Melin, 1941), and *Hyloscirtustorrenticola* (Duellman & Altig, 1978). Based on geographic location, species description, examination of the type material (Fig. 4A), and bioacoustic characteristics (e.g., differences in the rise time of the call and dominant frequency; see Table 4), we assign *Hyloscirtusalbopunctulatus* sensu stricto to Lineage A (Fig. 1). We redescribe it and redefine its distribution range based on the new evidence.

**Table 4.** Acoustic parameters comparing calls of *Hyloscirtusalbopunctulatus* (Lineage A), *H.maycu* sp. nov. (Lineage B), *H.elbakyanae* sp. nov. (Lineage D), *H.phyllognathus* sensu stricto (Lineage E), *H.* UCS 2 (Lineage F), *H.dispersus* sp. nov. (Lineage G) and *H.torrenticola* from type locality. Mean ± SD is given with range in parentheses. Five calls were analyzed per individual.

Parameter	*H.albopunctulatus* (*n* = 7)	*H.maycu* sp. nov. (*n* = 1)	*H.elbakyanae* sp. nov. (*n* = 2)	*H.phyllognathus* sensu stricto (*n* = 1)	UCS 2 (*n* = 1)	*H.dispersus* sp. nov. (*n* = 13)	*H.torrenticola* sensu stricto (*n* = 2)
**Series of calls**	2 – > 70	10–13	4–13	3–4	3	1–8	3–4
**Rise time of the call (s)**	2.28 ± 0.80 (1.43–3.69)	0.832	0.57 ± 0.05 (0.53–0.60)	0.28	0.17	0.61 ± 0.50 (0.053–2.01)	0.16 ± 0.017 (0.148–0.173)
**Call duration (s)**	0.051 ± 0.005 (0.045–0.058)	0.053	0.06 ± 0.001 (0.055–0.057)	0.04	0.05	0.11 ± 0.015 (0.082–0.130)	0.03 ± 0.001 (0.026–0.028)
**Inter-call duration (s)**	0.30 ± 0.06 (0.17–0.35)	0.09	0.12 ± 0.007 (0.11–0.12)	0.06	0.05	0.33 ± 0.044 (0.270–0.440)	0.07 ± 0.007 (0.060–0.070)
**Dominant frequency (Hz)**	2149.84 ± 137.36 (1903.42–2309.0)	2343.8	2321.29 ± 127.86 (2230.88–2411.7)	2177.0	1851.9	2795.41 ± 138.68 (2573.05–3014.60)	2743.79 ± 48.22 (2709.60–2777.80)
**Fundamental frequency (Hz)**	1214.12 ± 184.71 (1068.72–1571.90)	1171.90	1184.35 ± 30.48 (1162.8–1205.9)	1974.6	1851.9	2700.63 ± 195.66 (2210.80–2924.20)	2743.75 ± 48.16 (2709.70–2777.80)
**Bandwidth of the call (Hz)**	378.51 ± 24.98 (344.5–421.9)	515.6	581.4 ± 91.36 (516.8–646)	372.5	416.3	359.06 ± 12.71 (343.1–383.85)	459.0 ± 36.06 (433.50–484.50)

Based on genetic, morphological (e.g., differences in head width and tibia length), bioacoustic (e.g., differences in call duration, distance between calls and dominant frequency), and geographic data (e.g., considering the distance of Peruvian populations) (see Table 4 and Suppl. material 1: table S5), we conclude that *Hyloscirtusphyllognathus* sensu stricto is not distributed in Ecuador (see also Almendáriz et al. 2014). The ABGD and bPTP analyses indicate that Peruvian populations of “*Hyloscirtusphyllognathus*” represent two species (Lineages E and F). We tentatively assign *Hyloscirtusphyllognathus* sensu stricto to Lineage E as it is the closest to the type locality of the species (~ 50 km in a straight-line SSE of Roque, in San Martín, Perú). Advertisement calls of both lineages differ in dominant frequency and duration (Table 4). We could not make statistical comparisons as we only had one call per population. Likewise, morphological measurements of the individual of Lineage F overlap with the morphological space of individuals of Lineage E. More data is needed to confirm the assignment of Lineage E to *H.phyllognathus* and the status of Lineage F.

The species description of *H.torrenticola* (Duellman and Altig 1978) and photographs of its type material (Fig. 4B) are inconclusive to enable differentiation of this species from other Amazonian group members. However, advertisement calls from its type locality indicate that it represents a species distinct from the other Amazonian lineages (e.g., differences in call duration and dominant frequency; see Table 4). Therefore, we do not assign *Hyloscirtustorrenticola* to any of the Amazonian lineages (A–G) included in our phylogeny.

**Figure 5.**
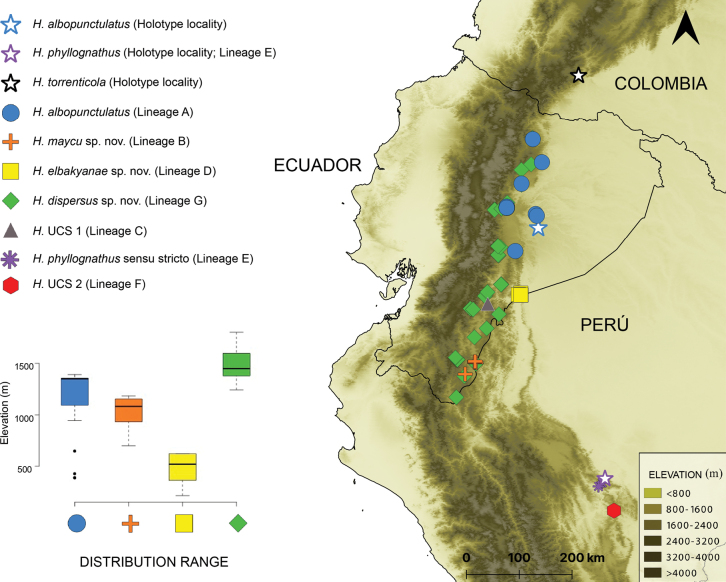
Geographic distribution of Amazonian species of the *Hyloscirtusbogotensis* group. Stars represent the type locality of *H.albopunctulatus*, *H.phyllognathus* and *H.torrenticola*. Symbol colors in the map match those of the lineages in the phylogeny. Boxplot comparing the elevational ranges are shown, which excludes *H.phyllognathus* and *H.torrenticola* due to the small sample.

Based on the assignments of the available binomials, we conclude that there are three new species that we describe below: Lineage B (*Hyloscirtusmaycu* sp. nov.), Lineage D (*Hyloscirtuselbakyanae* sp. nov.) and Lineage G (*Hyloscirtusdispersus* sp. nov.). Lineage C is left undescribed because its single specimen, QCAZ 48199, is a juvenile. See Fig. 5 for the new species’ geographic distribution. The haplotype network for the nuclear gene C-myc is consistent with the recognition of the three species as it shows that they lack shared haplotypes (Suppl. material 1: fig. S7).

### ﻿Morphological variation

**Figure 6.**
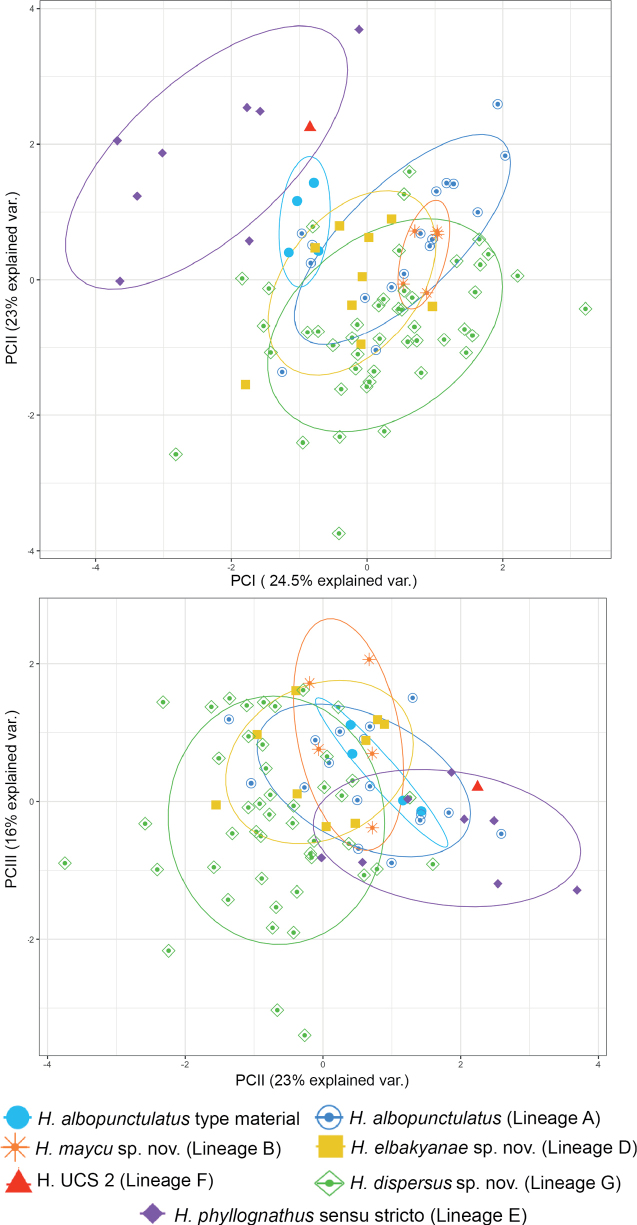
Principal components (PC) of the morphometric analysis. Seven size-corrected morphological variables were analyzed for adult females and males of *Hyloscirtusalbopunctulatus* (including paralectotypes), *H.maycu* sp. nov., *H.elbakyanae* sp. nov., *H.dispersus* sp. nov., *H.phyllognathus* sensu stricto, and one individual of Lineage F (UCS 2). Normal data ellipses are shown by group. The contribution of each principal component to explain total variation is shown in parenthesis. Results of the PC analysis are shown in Suppl. material 1: table S6.

**Figure 7.**
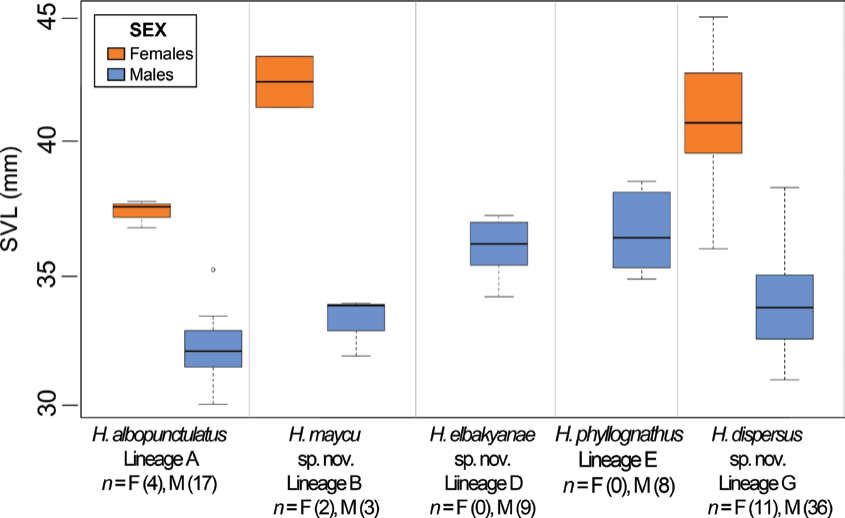
Boxplot of the snout-vent length (SVL) comparison analysis. SVL measurements of adult males and females of *Hyloscirtusalbopunctulatus*, *H.maycu* sp. nov., *H.elbakyanae* sp. nov., *H.dispersus* sp. nov., and *H.phyllognathus*. The line in the middle of the box represents the median and the upper and lower ends of the box are the 75% and 25% quartiles, respectively.

Morphological variables from adult males and females are summarized in Suppl. material 1: table S5 and compared in Fig. 6. The morphometric PCA of 74 males and 17 females resulted in three principal components with an eigenvalue > 1, accounting for 63.3% of total variation. PC I was positively correlated with eye diameter and head width, PC II with foot length and internarial distance, and PC III with internarial distance and femur length (see Suppl. material 1: table S6). The morphometric space shows high overlap among all species, except for *H.phyllognathus* sensu stricto, between PC1 and PC2. Moreover, paired comparisons for SVL showed intra and interspecific differentiation (Fig. 7). There is sexual dimorphism as females are larger than males in *H.albopunctulatus* (*t* = 14.15, df = 15, *p*-value < 0.01), *H.dispersus* sp. nov. (*t* = 7.23, df = 11, *p*-value < 0.01) and *H.maycu* sp. nov. (*t* = 7.78, df = 2, *p*-value = 0.01); females of *H.elbakyanae* sp. nov. and *H.phyllognathus* are unknown. Among species, females of *H.albopunctulatus* are smaller than those of *H.dispersus* sp. nov. (*t* = -4.27, df = 11, *p*-value = 0.001). Males of *H.elbakyanae* sp. nov. are larger than males of *H.albopunctulatus*, *H.maycu* sp. nov. and *H.dispersus* sp. nov. (*t* = -7.81, df = 18, *p*-value < 0.001; *t* = -3.57, df = 4, *p*-value = 0.03; *t* = 4.04, df = 20, *p*-value < 0.001). Males of *H.phyllognathus* are also larger than *H.albopunctulatus*; *H.maycu* sp. nov. and *H.dispersus* sp. nov. (*t* = 7.42, df = 11, *p*-value < 0.001; *t* = 4.05, df = 4, *p*-value = 0.01; *t* = 4.39, df = 12, *p*-value < 0.001).

### ﻿Advertisement calls

**Figure 8.**
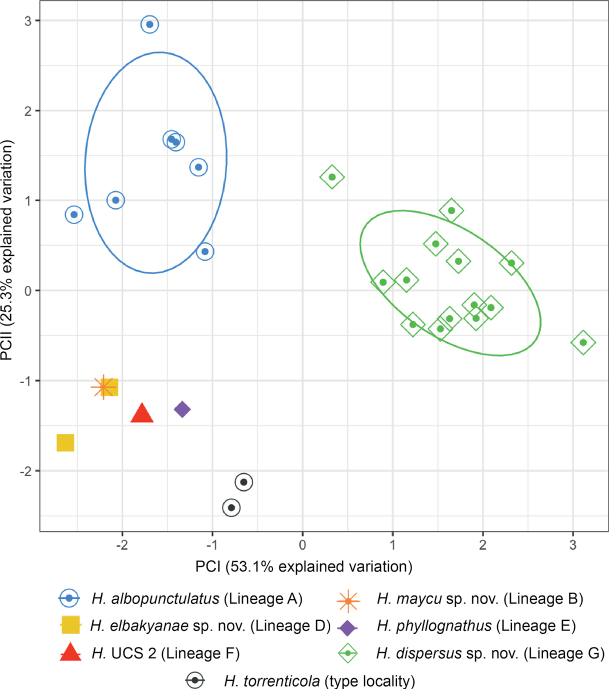
Principal components (PC) of the bioacoustic analysis. Six acoustic variables of advertisement calls were analyzed of *Hyloscirtusalbopunctulatus*, *H.maycu* sp. nov., *H.elbakyanae* sp. nov., *H.dispersus* sp. nov., *H.phyllognathus* sensu stricto, *H.* UCS 2 (Lineage F) and *H.torrenticola* sensu stricto. Normal data ellipses are shown by group. The contribution of each principal component to explain total variation is shown in parenthesis. Results of the PC analysis are shown in Suppl. material 1: table S7.

**Figure 9.**
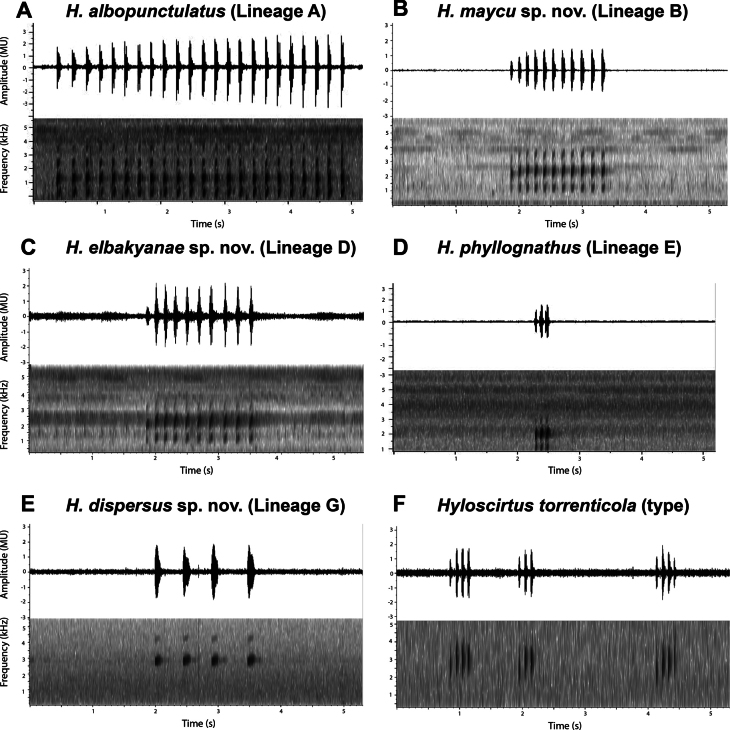
Advertisement calls of Amazonian species of the *Hyloscirtusbogotensis* species group. Oscillograms above with their corresponding spectrograms below are shown for each species.

Bioacoustic variables are summarized in Table 4 and call variation is shown in Fig. 8. The advertisement calls of all analyzed species are emitted in a series of 1 to 13 calls. The PCA of calls from 27 individuals resulted in two principal components with eigenvalues > 1. The two PCs accounted for 78.4% of the total variance. PC I was positively correlated with dominant and fundamental frequency and call duration. PC II was positively correlated with rise time of the call and intercall interval (see Suppl. material 1: table S7). The acoustic space shows clear differentiation for *H.albopunctulatus*, *H.dispersus* sp. nov., and *H.torrenticola* (Fig. 8). In contrast, the calls from *H.maycu* sp. nov. and *H.elbakyanae* sp. nov. overlap. Pairwise comparisons showed significant differences. *Hyloscirtusdispersus* sp. nov., has a significantly longer call (*t* = -11.46, df = 16.807, *p-value* < 0.001) with a higher dominant frequency (*t* = -8.227, df = 19, *p-value* < 0.001) than the sympatric *H.albopunctulatus*. *Hyloscirtustorrenticola* sensu stricto has a significantly shorter call (*t* = 17.46, df = 13.897, *p-value* < 0.001) than *H.dispersus* sp. nov., and a higher dominant frequency (*t* = -10.128, df = 5.5338, *p-value* < 0.001) than *H.albopunctulatus* – the two geographically closest lineages. We could not statistically compare the calls of *H.phyllognathussensu lato* from Peru as we only had one call per lineage. However, the spatial segregation in the PC is obvious in relation to *H.dispersus* sp. nov. (mainly regarding the rise time of the call, call duration and intercall duration, see Table 4)– its geographically and genetically closest species. Consequently, we did not assign *H.torrenticola* and *H.phyllognathus* to any of our Ecuadorian lineages (Fig. 5). Call differences are also noticeable in the spectrograms (Fig. 9).

### ﻿Environmental characteristics

**Figure 10.**
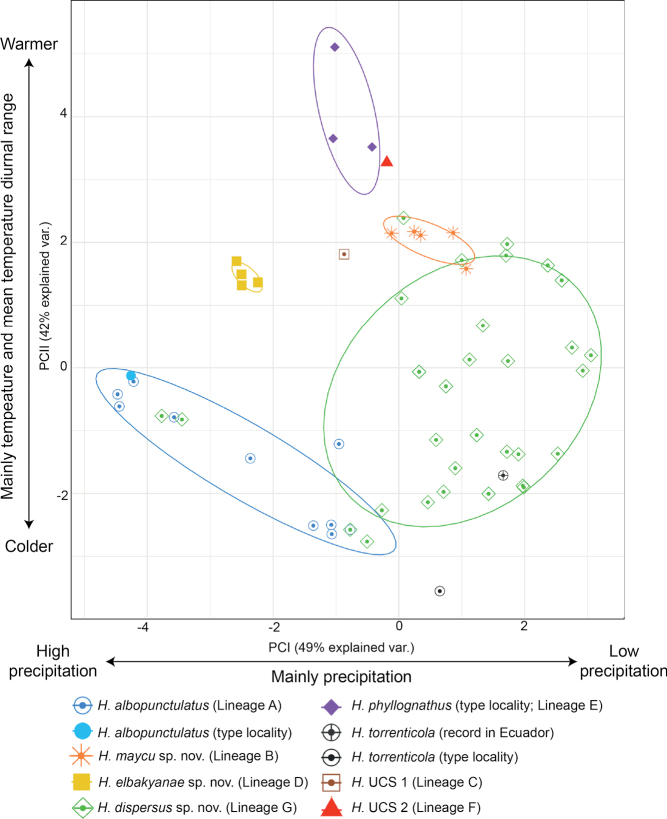
Principal components (PC) of the environmental envelope analysis. Eight environmental variables were analyzed of localities of *Hyloscirtusalbopunctulatus*, *H.maycu* sp. nov., *H.elbakyanae* sp. nov., *H.dispersus* sp. nov., *H.phyllognathus* sensu stricto, H. UCS 1 an individual from Lineage F (UCS 2), and *H.torrenticola* sensu stricto. Normal data ellipses are shown by group. The contribution of each principal component to explain total variation is shown in parentheses. The results of the PC analysis are shown in Suppl. material 1: table S8.

The PCA analysis of 67 localities summarized environmental variables in two principal components (with eigenvalues > 1). They accounted for 91.8% of the total variance (Fig. 10). PC I was negatively correlated with precipitation variables while PC II was positively correlated with temperature diurnal range, temperature of the warmest month, and annual temperature (see Suppl. material 1: table S8). The environmental space PC I vs. PC II shows that *H.elbakyanae* sp. nov. occurs in wetter environments relative to *H.dispersus* sp. nov. (*t* = -13.97, df = 41.95, *p-value* < 0.001) and warmer environments relative to *H.albopunctulatus* (*t* = -8.95, df = 10.99, *p-value* < 0.001). The three Peruvian localities of *H.phyllognathus* are warmer than those of *H.albopunctulatus* and *H.elbakyanae* sp. nov. (*t* = -9.197, df = 3.63, *p-value* = 0.001 and *t* = -5.15, df = 2.08, *p-value* = 0.033). *Hyloscirtusmaycu* sp. nov. occurs on dryer environments than *H.albopunctulatus* (*t* = -5.83, df = 13.08, *p-value* < 0.001). *Hyloscirtustorrenticola* has a low sample size, however, it appears different from *H.maycu* sp. nov., *H.elbakyanae* sp. nov., and the individuals from Peru (*H.phyllognathus* sensu stricto + Lineage F) as it seems to occur under colder conditions. Regarding altitudinal ranges, *H.elbakyanae* sp. nov. has been registered at lower elevations (214–622 m) and does not overlap with the elevation range of *H.maycu* sp. nov. (882–1183) and *H.dispersus* sp. nov. (1262–1807 m) (Fig. 5).

### ﻿Systematic accounts

#### ﻿The taxonomic status of *Hylaalbopunctulata* Boulenger, 1882

The description of *Hylaalbopunctulata* was based on five syntypes, all adult males: BMNH 1880.12.5.159–162 from “Ecuador’’ and 1880.12.5.230 from “Sarayacu, Ecuador” (= Sarayacu, Provincia Pastaza, Ecuador). In accordance with amended Article 74.7.3 of the Code (ICZN 2003) we designate the adult male BMNH 1880.12.5.230 as lectotype of *Hylaalbopunctulata* Boulenger, 1882 in order to clarify its precise type locality and by consequence the application of the name, with specimens BMNH 1880.12.5.159–162 becoming paralectotypes. The lectotype BMNH 1880.12.5.230 (formerly 80.12.5.230; Fig. 4A), is the only type with precise locality information (see the Taxonomy). Therefore, the type locality for *Hylaalbopunctulata* becomes “Sarayacu, Ecuador”.

We assign the binomial *Hyloscirtusalbopunctulatus* to Lineage A (Fig. 1) based on morphological and acoustic evidence and the location of the type locality, which overlaps with the distribution range of Lineage A exclusively (Fig. 5). In addition, localities from Lineage A are at lower elevations (389–1391 m) than *H.dispersus* sp. nov. (1262–1807 m), the closest species to the type locality. The description of the species is consistent with the morphology of Lineage A. Shared characters include vomerine teeth in a scarcely interrupted series, snout in males rounded, canthus rostralis distinct, tympanum very small and indistinct, subarticular tubercles indistinct and SVL = 33 mm in males (Boulenger 1882). Based on this evidence, we conclude that *H.albopunctulatus* corresponds to Lineage A in our phylogeny.

##### ﻿ Hyloscirtus albopunctulatus

Taxon classificationAnimaliaAnuraHylidae

(Boulenger, 1882)

8A6BDAA1-7B46-595E-BCE5-CE0D1B0346E4

Figs 4 , 5 , 7 , 9 , 11 , 13 , 14 , 15 Common name: Standard English name: White-speckled tree frog Standard Spanish name: Rana de torrente de puntos blancos ( Frank and Ramus 1996)

Hyla albopunctulata Boulenger, 1882: 385, fig. 4. Type locality: Sarayacu, Ecuador.

###### Type material.

Designated ***lectotype*** (Fig. 4A): BMNH 1880.12.5.230, adult male, from “Sarayacu, Ecuador”. ***Paralectotypes***: BMNH 1880.12.5.159–162 adult males from “Ecuador”

###### Definition.

In this section, coloration and characters refer to preserved specimens unless otherwise mentioned, based on four adult females and 17 adult males, including the paralectotypes. *Hyloscirtusalbopunctulatus* can be diagnosed by the combination of the following characters: (1) mean SVL 32.3 mm in adult males (range 30.3–35.5; *n* = 17) and mean SVL 37.8 mm in adult females (range 37.1–38.1; *n* = 4) (Suppl. material 1: table S5, Fig. 7); (2) white supralabial stripe present; (3) tympanum round, inconspicuous in males and distinct in females, supratympanic fold present and unpigmented; (4) white ulnar and tarsal folds present and thick; (5) subarticular tubercles varying from small to inconspicuous in hands and feet; (6) supernumerary tubercles inconspicuous in hands and feet; (7) calcar tubercle absent; (8) pericloacal spots well-defined; (9) all surfaces plain cream with a combination of black and white spots in the dorsum; (10) in life, dorsal surfaces and flanks olive green to yellowish green, covered with white spots and with or without sparce or clumped black spots; axillar and inguinal regions yellowish or blueish; venter and anterior and posterior surfaces of thighs yellow; other ventral surfaces silver, brownish or greenish; yellow pericloacal spots; webbing yellow orange; iris clam shell with black or sand dune reticulations; (11) the advertisement call consist of a single note, with a mean duration of 0.051 ± 0.005 s, a mean dominant frequency of 2147.84 ± 137.36 Hz and a fundamental frequency of 1214.12 ± 184.71 Hz. The call can be repeated consecutively for an indefinite number of times (2 – > 70) in a series of calls.

###### Diagnosis.

**Figure 11.**
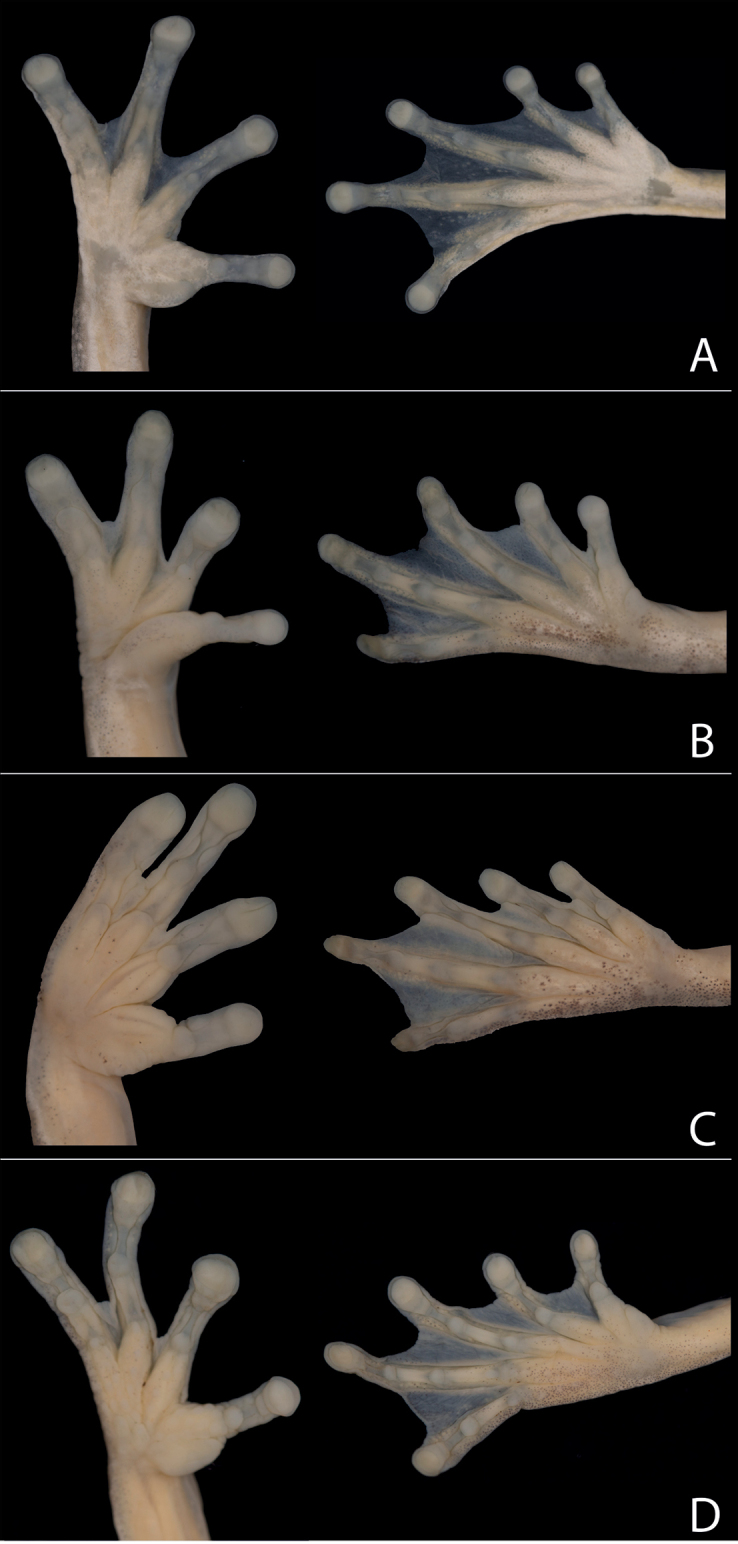
Ventral views of right hands and feet of the species **A***H.albopunctulatus*, QCAZ 59825, SVL: 37.9 mm, female **B***H.maycu* sp. nov., QCAZ 67087, SVL: 34.1 mm, male **C***H.elbakyanae* sp. nov., QCAZ 53808, SVL: 36.1 mm, male **D***H.dispersus* sp. nov., QCAZ 52006, SVL: 32.6 mm, male.

**Figure 12.**
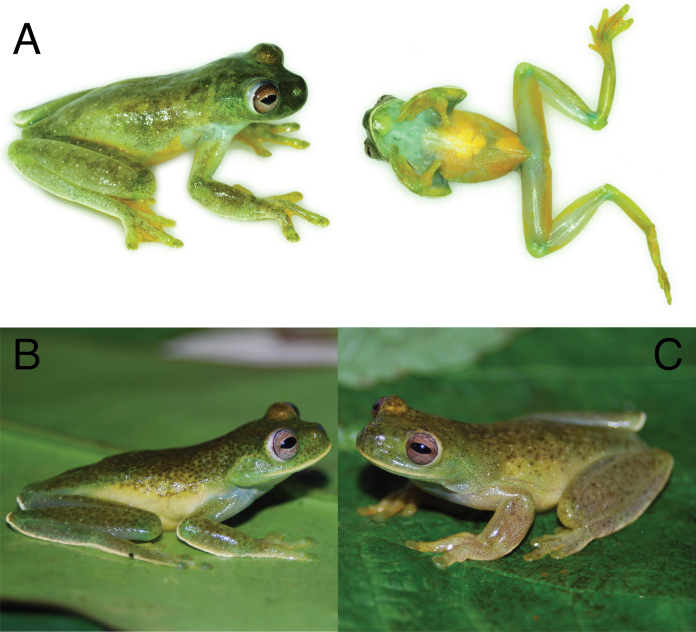
Photographs of individuals of *Hyloscirtusphyllognathus* and *H.torrenticola* species in life **A** dorsolateral and ventral views of a male of *H.phyllognathus* sensu stricto (CORBIDI 9590, Catarata Ahuashiyacu, San Martín, Perú) **B** dorsolateral view of a female of *H.torrenticola* (MAR 1938, Departamento de Caquetá, Colombia) **C** dorsolateral view of a male of *H.torrenticola* (MAR 1974, same locality as B). Photographs by Alessandro Catenazzi (**A**) and Marco Rada (**B, C**).

Characters in this section pertain to preserved specimens unless otherwise noticed. Coloration refers to live specimens. The most similar species to *Hyloscirtusalbopunctulatus* living in the Amazon basin are *H.maycu* sp. nov., *H.elbakyanae* sp. nov., *H.dispersus* sp. nov., *H.phyllognathus*, and *H.torrenticola. Hyloscirtusalbopunctulatus* differs by having a white supralabial stripe (absent in *H.elbakyanae* sp. nov.), a supratympanic fold (absent in all species except in *H.dispersus* sp. nov.), a thick tarsal fold (rudimentary in *H.maycu* sp. nov., *H.elbakyanae* sp. nov., and *H.phyllognathus*), small to inconspicuous subarticular tubercles in hands and feet (conspicuous in hands and feet in *H.maycu* sp. nov., *H.elbakyanae* sp. nov., and *H.dispersus* sp. nov.; Fig. 11), inconspicuous supernumerary tubercles in hands and feet (conspicuous in hands in *H.dispersus* sp. nov.), an absent calcar tubercle (present in *H.dispersus* sp. nov. and *H.phyllognathus*), well-defined pericloacal spots (ill-defined or absent in *H.maycu* sp. nov., *H.elbakyanae* sp. nov., and *H.torrenticola*), and a clam shell iris with black or sand dune reticulations (a clam shell iris with dark pinkish or leather reticulations in *H.maycu* sp. nov. and *H.elbakyanae* sp. nov., pearl or pinkish iris with leather reticulations in *H.dispersus* sp. nov. and bronze iris in *H.torrenticola*, Fig. 12).

The advertisement call of *H.albopunctulatus* has a rise time of 2.28 ± 0.80 s (shorter in *H.maycu* sp. nov. with 0.832 s and in *H.elbakyanae* sp. nov. with 0.57 ± 0.05 s), a dominant frequency of 2149.84 ± 137.36 Hz and a fundamental frequency of 1214.12 ± 184.71 Hz (higher dominant frequency of 2795.41 ± 138.68 Hz and fundamental frequency of 2210.8–2924.2 Hz in *Hyloscirtusdispersus* sp. nov.). *Hyloscirtustorrenticola* also has a higher dominant frequency of 2743.79 ± 48.22 Hz and a fundamental frequency of 2743.75 ± 48.16 Hz. *Hyloscirtusalbopunctulatus* has a call duration of 0.051 ± 0.005 s (longer in *H.dispersus* sp. nov. with 0.11 ± 0.015 s). *Hyloscirtus*albopunctulatus has an intercall duration of 0.30 ± 0.06 s (shorter in *H.phyllognathus* with 0.05 s and in *H.torrenticola* with 0.07 ± 0.007 s) (Table 4, Fig. 9; Melin 1941; Duellman and Altig 1978; Rivera-Correa 2016). Moreover, all males of *H.albopunctulatus* were registered calling from under rocks next to streams (*n* = 5; Pontificia Universidad Católica del Ecuador 2024), while all males of *H.dispersus* sp. nov. have been found calling while perching in vegetation over streams (*n* = 14; Pontificia Universidad Católica del Ecuador 2024).

###### Variation.

**Figure 13.**
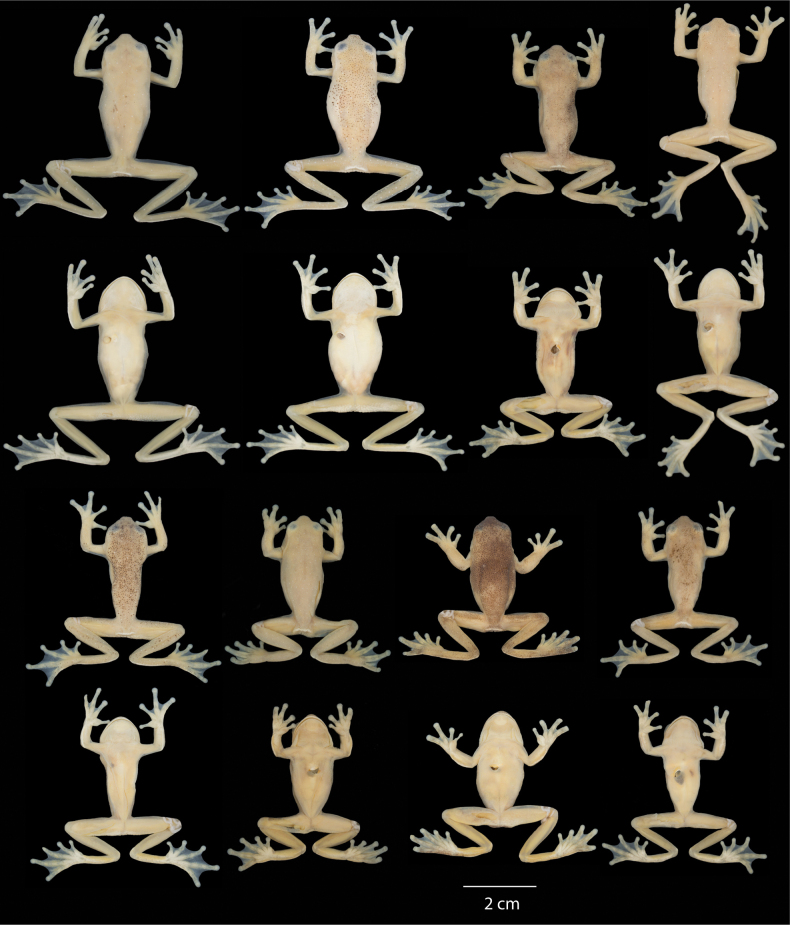
Variation of preserved specimens of *Hyloscirtusalbopunctulatus*. Dorsal and ventral views. From left to right, first and second rows: QCAZ 62188, 59825 (adult females), QCAZ 59817, 54111 (adult males); third and fourth rows: QCAZ 59823, 59827, 59814 (adult males), QCAZ 59809 (subadult female). See Suppl. material 1: table S1 for locality information. All specimens are shown at the same scale.

**Figure 14.**
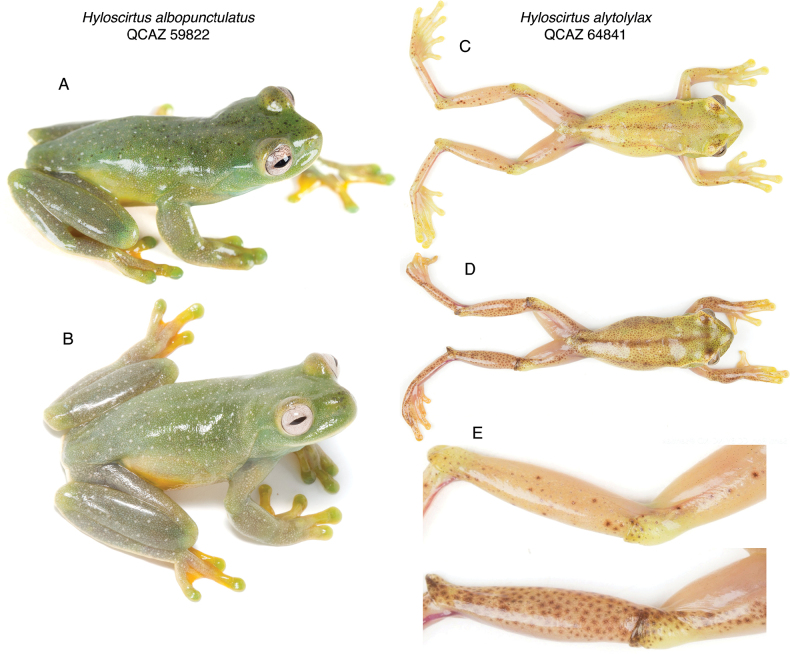
Phenotypic plasticity in dorsal color of *Hyloscirtus*. Left column: *H.albopunctulatus*, QCAZ 59822, adult male. Right column: *Hyloscirtusalytolylax*, QCAZ 64841, adult male. Photos in **A, B** were taken within 17 days of each other; photos in **C, D** within 16 minutes **E** Inset of the hindleg of QCAZ 64841 showing chromatophore change.

Dorsal and ventral variation of adult preserved specimens is illustrated on Fig. 13. In preservative, dorsum varies from cream with white spots distributed throughout the body and limbs and thick black spots scattered across the body (e.g., QCAZ 59825) and limbs (e.g., QCAZ 59823), with minute black spots scattered in the entire body (e.g., QCAZ 59817) or accumulated in the head (e.g., QCAZ 59824), with dark flecks in body and limbs (e.g., QCAZ 59809), with barely visible minute black spots (e.g., QCAZ 62188) or absent black spots (e.g., QCAZ 59827), to cream with barely visible white spots and minute and thick black spots covering almost the entire dorsum (e.g., QCAZ 59814). We found evidence of conspicuous intraindividual phenotypic plasticity in color. Photos of QCAZ 59825 taken within 14 days of each other show variation in the presence of black spots on the dorsum, head, and snout. Similarly, QCAZ 59822, after 17 days, gained abundant black spots on the dorsum and head and increased the conspicuousness of the dark reticulations on the iris (Fig. 14A, B). We did not find phenotypic plasticity in ventral coloration. Ventral surfaces vary from cream (e.g., QCAZ 59825) to darker cream (e.g., QCAZ 59817). Mental gland in males varies from whitish cream (e.g., QCAZ 59814) to darker cream (e.g., QCAZ 59827).

**Figure 15.**
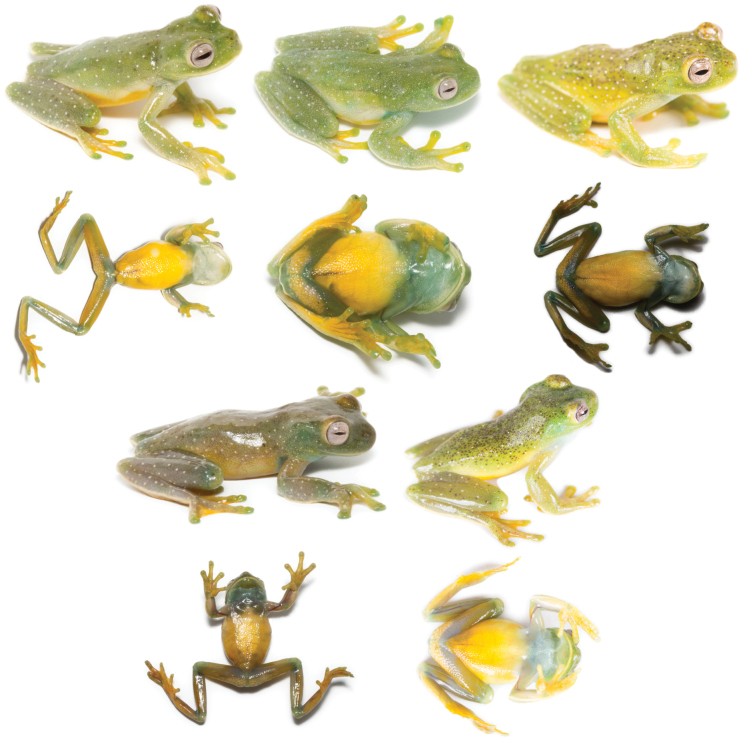
Variation in life of *Hyloscirtusalbopunctulatus*. Dorsolateral and ventral views. From left to right, first and second rows: QCAZ 59825, 59811, 59824 (adult females); third and fourth rows: QCAZ 59808, 59823 (adult males).

In life (Fig. 15), the dorsum varies from light green with white spots scattered throughout the body and limbs, and few spread black marks (e.g., QCAZ 59825) or without black spots or marks (e.g., QCAZ 59811), yellowish green with white spots covering the entire body and limbs and spread black marks, accumulated in the anterior part of the body (e.g., QCAZ 59824) or minute and thick blacks spots across the body (e.g., QCAZ 59823) to brownish green with white spots in all the body and limbs and few black marks randomly dispersed (e.g., QCAZ 59808). Venter and posterior surfaces of thighs vary from yellow (e.g., QCAZ 59811) to greenish yellow (e.g., QCAZ 59808). Other ventral surfaces vary from whitish (e.g., QCAZ 59823) to brownish green (e.g., QCAZ 59824). Iris varies from clam shell with thin (e.g., QCAZ 59825) to thick black or sand dune reticulations (e.g., QCAZ 59823). Examined specimens are listed in Suppl. material 1: tables S1, S9.

###### Distribution and natural history.

*Hyloscirtusalbopunctulatus* is known from seven localities, from North to Central eastern Ecuador at elevations between 389 and 1391 m (Fig. 5). Biogeographic regions are Amazon Humid Tropical Forest, Eastern Foothill Forest, and Eastern Montane Forest (Ron et al. 2022). They are nocturnal and associated with streams near ravines. Males call under rocks in ravines, leaf litter, streams, small creeks, caves, or cracks. Several individuals have been found perching on vegetation up to 2.5 m but have not been found calling there. The species occurs in sympatry with *Hyloscirtusdispersus* sp. nov.

###### Advertisement call.

We analyzed 26 calls from seven individuals. Six males (QCAZ 59813, 59815, 59817 and three non-collected males) from Comunidad Zarentza, Llanganates National Park, Provincia Pastaza, on 17–24 February 2017, air temperature 19–21 °C, recorded by D. Velalcázar and D. Rivadeneira. One individual (QCAZ 48503) from Reserva Río Bigal, Provincia Orellana, recorded by M. Read, on 22 May 2010. The advertisement call consists of a single tonal note, repeated a highly variable number of times in a series of calls (Fig. 9A). We found from two to more than 70 consecutive calls before long silence periods. One male (QCAZ 59815) called with short pauses during 4.08 s, broadcasting 926 calls. The average call duration is 0.051+0.005 s with an average inter-call interval of 0.30 ± 0.06 s. The average dominant frequency of the call is 2149.8 ± 137.36 Hz. Other call parameters are listed in Table 4.

###### Conservation status.

The distribution polygon of *Hyloscirtusalbopunctulatus* is 7921 km^2^. Habitat destruction for agriculture and cattle is rising within its distribution range (Ministerio del Ambiente 2013). However, they also inhabit undisturbed and protected areas like Parque Nacional Llanganates. Its tolerance to disturbed forests is unknown. Given its distribution range being less than 20000 km^2^ and by having less than 10 known localities, we propose that *H.albopunctulatus* remains assigned to the Red List category Vulnerable (B1abiii).

##### ﻿ Hyloscirtus maycu sp. nov.

Taxon classificationAnimaliaAnuraHylidae

701B7DE6-18A6-5A56-9AFE-C2FD989D1980

https://zoobank.org/2E9E3BAC-DE92-4DB2-80C2-FCFBDDB7065E

**Figure 16.**
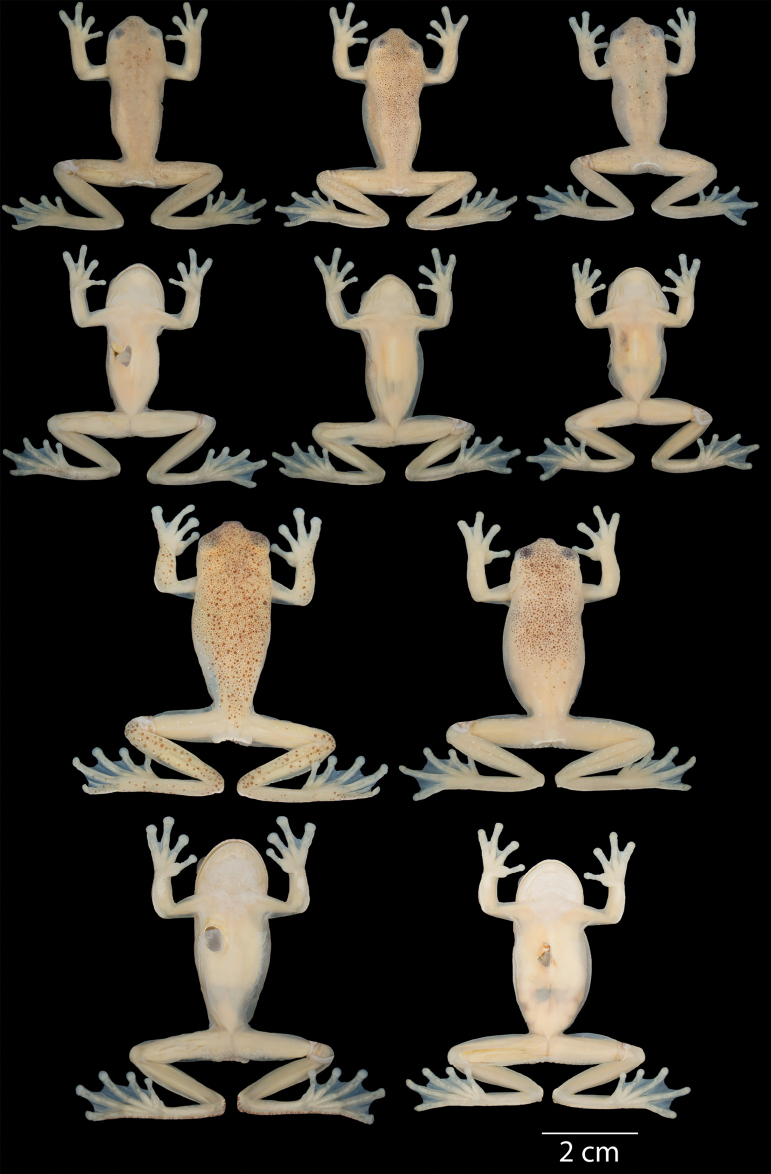
Variation of preserved specimens of *Hyloscirtusmaycu* sp. nov. Dorsal and ventral views. From left to right, first and second rows: QCAZ 67087 (holotype, adult male), QCAZ 67082, 68055 (adult males); third and fourth rows: QCAZ 67081, 67086 (adult females). See Suppl. material 1: table S1 for locality information. All specimens are shown at the same scale.

**Figure 17.**
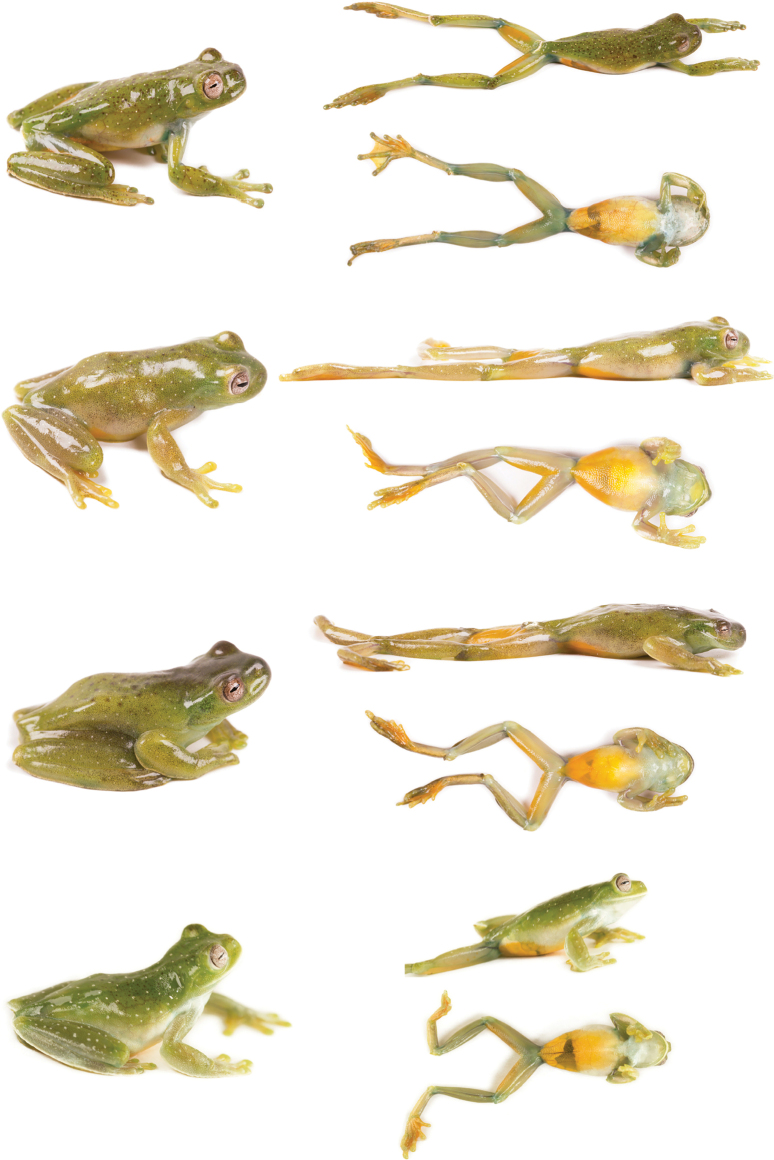
Variation in life of *Hyloscirtusmaycu* sp. nov. Dorsolateral, lateral, and ventral views. From top to bottom: QCAZ 67081 (adult female), QCAZ 67082 (adult male), QCAZ 67087 (holotype, adult male), QCAZ 67086 (adult female).

Figs 5 , 7 , 9 , 10 , 11 , 16 , 17 Common name: Proposed standard English name: Maycu stream frog Proposed standard Spanish name: Rana de torrente de Maycu

###### Type material.

***Holotype***. • QCAZ 67087 (Figs 16, 17), field no. SC 56707, adult male from Ecuador, Provincia Zamora Chinchipe, Reserva Natural Maycu, plateau (4.2067°S, 78.6326°W), 882 m above sea level, collected by D. Almeida, K. Nusirquia, D. Núñez, D. Paucar, F. Hervas, J. Ortega, A. Achig, S. Pillajo, R. Gavilanez, and J. Mora on 27 February 2017. A 3D model of the holotype is available at the Sketchfab platform (https://skfb.ly/oSqqr). ***Paratypes***. • All from Ecuador, Provincia Zamora Chinchipe. Collected with the holotype, QCAZ 67081, 67086 adult females, QCAZ 67082 adult male, QCAZ 67084, 67085 juveniles, QCAZ 67083 tadpole, 959–1219 m of elevation, collected on 23 and 25 February 2017 and 01 March 2017. Nuevo Paraíso, camp near Río Nangaritza, Cordillera del Oso (4.4442°S, 78.8134°W), 1127 m, QCAZ 68055 adult male, collected on 16 May 2017 by K. Nusirquia, Darwin Núñez, Andrea Calispa

###### Definition.

In this section, coloration and characters refer to preserved specimens unless otherwise mentioned. The definition and diagnosis are based on two adult females and three adult males. *Hyloscirtusmaycu* sp. nov. can be diagnosed by the combination of the following characters: (1) mean SVL 33.4 mm in adult males (range 31.9–34.2; *n* = 3) and mean SVL 42.7 mm in adult females (range 41.7–43.7; *n* = 2; Suppl. material 1: table S5; Fig. 7); (2) white supralabial stripe present or absent; (3) tympanum round, inconspicuous in males and distinct in females; supratympanic fold inconspicuous and unpigmented; (4) white ulnar and tarsal folds inconspicuous or conspicuous; (5) subarticular tubercles conspicuous, round and single, in hands and feet; (6) supernumerary tubercles inconspicuous in hands and feet; (7) calcar tubercle absent; (8) pericloacal spots well-defined; (9) all surfaces plain cream with a combination of black and white spots in the dorsum; (10) in life, dorsal surfaces and flanks olive green to crete with white spots and minute or thick black spots scattered over the body; axillar and inguinal regions blueish or mongoose; venter and posterior surfaces of thighs yellow; other ventral surfaces silver or greenish; bones and articulations blue; unpigmented pericloacal spots; webbing yellow orange; iris clam shell with thick dark pinkish to leather reticulations; (11) the advertisement call consists of a single note, with duration of 0.053 s (*n* = 1) and a dominant frequency of 2343.8 Hz and fundamental frequency of 1171.90 Hz. The call can be repeated consecutively from 10–13 times in a series of calls.

###### Diagnosis.

Characters in this section pertain to preserved specimens unless otherwise noticed. Coloration refers to life specimens. The most similar species to *H.maycu* sp. nov. living in the Amazon basin are *Hyloscirtusalbopunctulatus*, *H.elbakyanae* sp. nov., *H.dispersus* sp. nov., *H.phyllognathus*, and *H.torrenticola. Hyloscirtusmaycu* sp. nov. differs by the absence of a supratympanic fold (present in *H.albopunctulatus* and *H.dispersus* sp. nov.), an inconspicuous tarsal fold (present and thick in *H.albopunctulatus*, *H.phyllognathus*, and *H.torrenticola*), conspicuous subarticular tubercles in hands and feet (small to inconspicuous in hands and feet in *H.albopunctulatus*; Fig. 11), inconspicuous supernumerary tubercles in hands and feet (conspicuous in hands in *H.dispersus* sp. nov.), pericloacal spots ill-defined or absent (well-defined in *H.albopunctulatus* and *H.phyllognathus*), an absent calcar tubercle (present in *H.dispersus* sp. nov. and *H.phyllognathus*), and a clam iris with dark pinkish or leather reticulations (a clam shell iris with black or sand dune reticulations in *H.albopunctulatus* and a bronze iris in *H.torrenticola*, Fig. 12). Although our sample size is small, the available evidence indicates that *H.maycu* sp. nov. differs from *H.elbakyanae* sp. nov. by its smaller size (Fig. 7).

The advertisement call of *H.maycu* sp. nov. has a rise time of 0.8 s (longer in *H.albopunctulatus* with 2.28 ± 0.80 s and shorter in *H.elbakyanae* sp. nov. with 0.57 ± 0.05 s and in *H.torrenticola* with 0.16 ± 0.017 s), a dominant frequency of 2343.8 Hz and a fundamental frequency of 1171.9 Hz (higher dominant frequency in *H.dispersus* sp. nov. of 2795.41 ± 138.68 Hz and fundamental frequency of 2700.63 ± 195.66 Hz). *Hyloscirtustorrenticola* also has a higher dominant frequency of 2743.79 ± 48.22 Hz and a fundamental frequency of 2743.75 ± 48.16 Hz. *Hyloscirtusmaycu* sp. nov. has a call duration of 0.053 s (longer in *H.dispersus* sp. nov. of 0.11 ± 0.015 s) (Table 4, Fig. 9; Melin 1941; Duellman and Altig 1978; Rivera-Correa 2016).

Additionally, *H.maycu* sp. nov. inhabits elevations between 882 and 1183 m, while *H.elbakyanae* sp. nov. lives lower between 214 and 622 m and in wetter and warmer environments (Figs 5, 10).

###### Description of the holotype.

Description of characters based on the preserved specimen. Adult male (Figs 16, 17). Measurements (in mm): SVL 34.1; foot length 13.9, head length 9.7, head width 10.2, eye diameter 3.0, tympanum diameter 2.0, tibia length 17.2, femur length 17.2, internarial distance 3.1, inter-orbital distance 4.9. Head wider than long; body slender; snout rounded in dorsal and lateral view; distance from nostril to eye same as diameter of eye; canthus rostralis distinct; loreal region convex; internarial region slightly curved; top of the head slightly concave; nostrils not protuberant, round, directed anterolaterally; lips rounded, not flared; interorbital area slightly concave, longer than upper eyelid; tympanum and tympanic fold inconspicuous; tympanic annulus absent; tympanic membrane absent; mental gland present, diamond-shaped, well defined, extending ~ 1/3 the size of the head; dentigerous processes of vomers slightly curved, transversal and posterior to ovoid choanae, each process narrowly separated from each other and bearing five teeth each; tongue cordiform, widely attached to mouth floor; vocal slits and subgular vocal sac present.

Forearms slender; axillary membrane absent; fingers bearing dermal fringes and rounded discs; relative lengths of fingers I < II < IV < III; webbing formula I basal II 2–—3– III 2^+^—2–IV; subarticular tubercles prominent, round, single; supernumerary tubercles small; thenar tubercle absent; palmar tubercle small; prepollex present, not modified as a spine; nuptial pads absent; ulnar tubercles absent; outer ulnar fold present. Hindlimbs slender; toes bearing dermal fringes and rounded discs; relative length of toes I < II < III < V < IV; extensive toe webbing, formula I 1–—2– II 1^+^—2– III 1^+^—2– IV 2–—1^+^ V; outer tarsal fold present; tarsal tubercles absent; calcar tubercle absent; subarticular tubercles conspicuous, round and single; supernumerary tubercles inconspicuous; inner metatarsal tubercle present and round, outer absent. Skin on dorsum, flanks, dorsal and ventral surfaces of limbs, thighs, and venter smooth; cloacal opening directed posteriorly at upper level of thighs; cloacal fold thick.

###### Color of holotype in preservative

(Fig. 16). Dorsal surfaces of the head, dorsum, flanks, and limbs cream with few minute black and white spots scattered through the body. Venter, throat, and ventral surfaces of limbs plain cream. Mental gland pale cream. White supralabial stripe absent. Ulnar, tarsal, and cloacal folds white. Webbing cream. Other details are shown in Fig. 16.

###### Color of holotype in life

(Fig. 17). Based on digital photographs. Dorsal surfaces and flanks olive green with white spots and minute black spots and a few dispersed black marks. Head darker brown, probably because of the high accumulation of minute black spots. Venter and posterior surface of thighs yellow. Axillar and inguinal regions and other ventral surfaces greenish white. Yellowish mental gland. Ulnar, tarsal, and cloacal folds white. Webbing yellow orange. Iris clam shell with leather reticulations.

###### Variation.

Dorsal and ventral variation of adult specimens is illustrated on Fig. 16 (in preservative) and Fig. 17 (in life). In preservative, dorsum varies from cream with white spots scattered throughout the body and limbs with minute and thick darker brownish black spots scattered across the entire body and limbs (e.g., QCAZ 67081) or accumulated in the anterior part of the body (e.g., QCAZ 67086) to darker cream with scattered spots dispersed in the body and limbs and minute black spots covering the dorsum (e.g., QCAZ 67082) or barely visible minute black spots and a few black marks in the anterior part of the body (e.g., QCAZ 68055). Venter varies from paler (e.g., QCAZ 67086) to darker cream (e.g., QCAZ 68055). White supralabial stripe varies from absent (e.g., QCAZ 67082), ill-defined (e.g., QCAZ 67081) to well-defined (e.g., QCAZ 67086).

In life (Fig. 17), dorsum varies from olive green with scattered white spots throughout the body and limbs with thick black spots in the body and limbs (e.g., QCAZ 67081) or few black marks in the body (e.g., QCAZ 67082) to paler olive green with spread white marks covering the entire body and limbs and few black marks in the mid part of the body (e.g., QCAZ 67086). Axillar and inguinal regions vary from blue (e.g., QCAZ 67081) to whitish. Ventral surfaces besides venter and posterior thighs vary from greenish (e.g., QCAZ 67081) to whitish (e.g., QCAZ 67082). A white supralabial stripe is present (e.g., QCAZ 67086) or absent (e.g., QCAZ 67082). Iris varies from clam shell with thin leather reticulations (e.g., QCAZ 67086), thick pinkish reticulations (e.g., QCAZ 67081) or thick leather reticulations (e.g., QCAZ 67082).

###### Distribution and natural history.

*Hyloscirtusmaycu* sp. nov. is known only from its type locality in Provincia Morona Santiago and one locality in Provincia Zamora Chinchipe (airline distance 32 km), at elevations between 882 and 1183 m, on the foothills of Cordillera del Cóndor, in Ecuador (Fig. 5). Biogeographic region is Eastern Lower Montane Forest (Ron et al. 2022). This species lives in primary and secondary forests. They are nocturnal and associated with ravines and streams. Males call from vegetation up to 2 m high, on the edge of the streams. One individual was recorded calling under a rock, and another on a rock in a ravine with low flow. A metamorph was found (in March) on an island of rock in the middle of a stream, suggesting that its tadpoles develop in streams, like other *Hyloscirtus*. A tadpole (QCAZ 67083) was found on a pool next to a stream in February.

###### Advertisement call.

We analyzed five calls from one individual (QCAZ 67087) from Reserva Natural Maycu, Provincia Zamora Chinchipe, 27 February 2017, recorded by J. Ortega. The advertisement call consists of a single tonal note, repeated 10–13 times in a series of calls (Fig. 9B). Average call duration is 0.053 s with an average inter-call interval of 0.09 s. The average dominant frequency of the call is 2343.8 Hz. Other call parameters are listed in Table 4.

###### Conservation status.

The distribution polygon of *H.maycu* sp. nov. is 54.8 km^2^ (based on two localities). Its distribution range is small but overlaps with a protected area, Reserva Natural Maycu; however, it is also found in Cordillera del Cóndor, an area severely fragmented by deforestation due to agriculture, cattle raising, and threatened mining activities. In response to its distribution range being less than 20000 km2 and by having less than 10 known localities, we propose assigning *H.maycu* sp. nov. to the Red List category Vulnerable c (VU B1a).

###### Etymology.

The specific epithet *maycu* is used as a noun in apposition and refers to the type locality of the species, a protected area in Ecuador named Reserva Natural Maycu, managed by the NGO Naturaleza y Cultura Internacional. “Maycu” seems to be a derivation of the Shuar word “Maycua” or “Maycuwa”, which the Shuar people use to refer to some species of small trees of the genus *Brugmansia* (angel’s trumpet). The southern border of the Reserve has been invaded by illegal miners and provides an additional example of the threat that mining represents for biodiversity conservation (F. Serrano, in litt.).

##### ﻿ Hyloscirtus elbakyanae sp. nov.

Taxon classificationAnimaliaAnuraHylidae

9705E95E-6258-503B-B3C8-55E24B78066D

https://zoobank.org/6F4B822B-79BD-44CB-A810-B9531CAE982D

**Figure 18.**
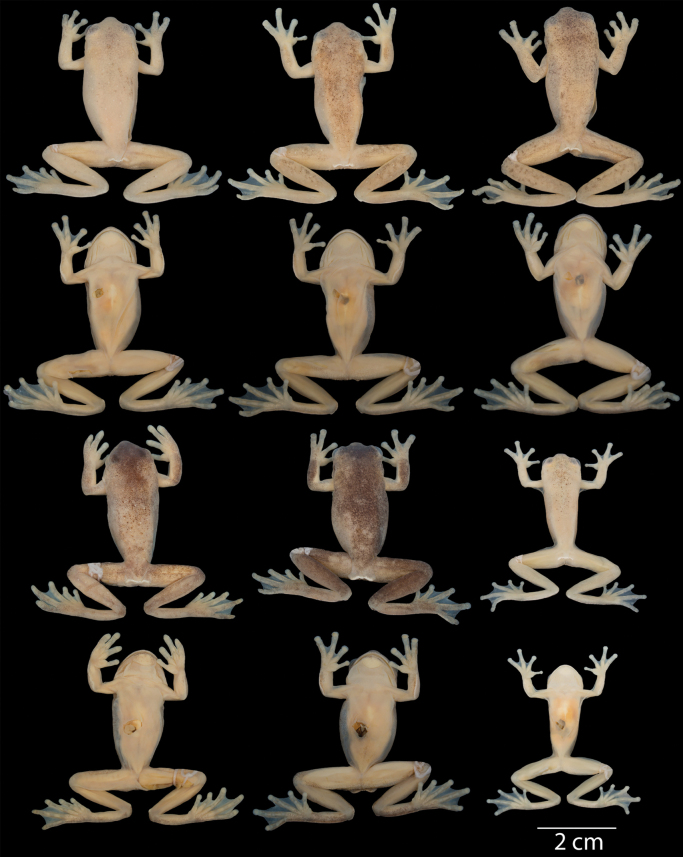
Variation of preserved specimens of *Hyloscirtuselbakyanae* sp. nov. Dorsal and ventral views. From left to right, first and second rows: QCAZ 72666, 73669, 72668 (adult males); third and fourth rows: QCAZ 53808 (holotype, adult male), QCAZ 72665, 73709 (adult males). See Suppl. material 1: table S1 for locality information. All specimens are shown at the same scale.

**Figure 19.**
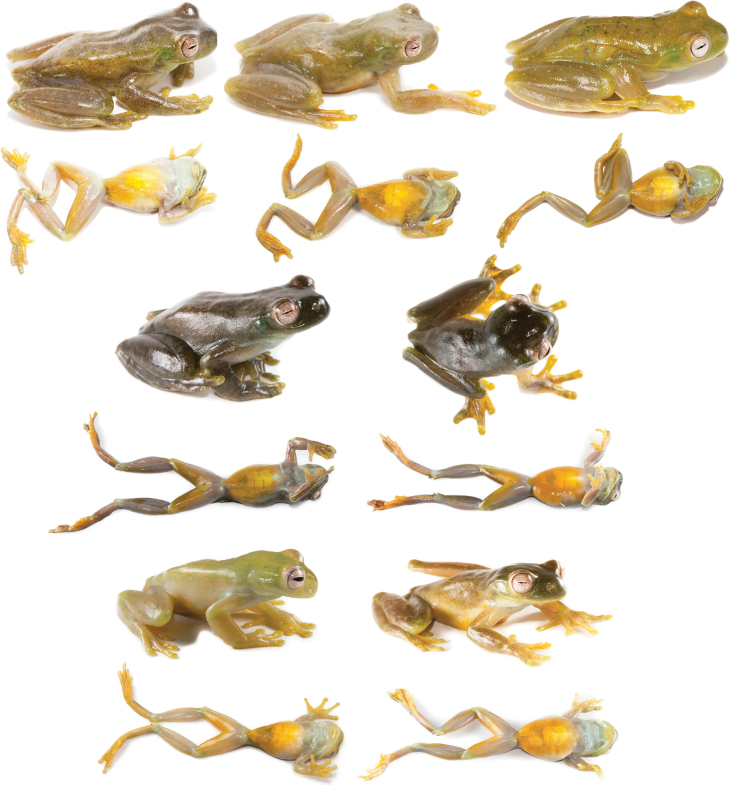
Variation in life of *Hyloscirtuselbakyanae* sp. nov. Dorsolateral and ventral views. From left to right, first and second rows: QCAZ 53808 (holotype, adult male), QCAZ 53807, 53831 (adult males); third and fourth rows: QCAZ 72667, 72665 (adult males); fifth and sixth rows: QCAZ 73709, 72669 (adult males).

Figs 5 , 7 , 9 , 11 , 18 , 19 Common name: Proposed standard English name: Elbakyan stream frog Proposed standard Spanish name: Rana de Torrente de Elbakyan

###### Type material.

***Holotype***. • QCAZ 53808 (Figs 18, 19), field no. SC 39260, adult male from Ecuador, Provincia Morona Santiago, Comunidad Shaime, near Mirador de la Virgen (2.975540°S, 77.80346°W), 622 m above sea level, collected by SRR, A. Merino, F. Ayala, T. Camacho, and M. Cohen on 23 July 2012. A 3D model of the holotype is available at Sketchfab platform (https://skfb.ly/oSXSH). ***Paratypes***. • All collected in Ecuador, Provincia Morona Santiago. Same locality and collection data as the holotype, QCAZ 53807, 53831 adult males. Surroundings of Río Shaime (2.9409°S, 77.8012°W), 511 m, QCAZ 72665–66 adult males, collected on 6 June 2018; • Tiwintza-Shaime road (2.9750°S, 77.7957°W), 211 m, QCAZ 72667, collected on 8 June 2018; • Mirador de la Virgen, Tiwintza-Shaime road (2.9756°S, 77.8015°W), 529 m, QCAZ 72668 adult male, collected on 8 June 2018; • Peñas-Shaime road (2.9663°S, 77.8468°W), 363 m, QCAZ 72669, collected on 9 June 2018; • Peñas-Shaime road, 2.8 km E Río Yaupi (2.9663°S, 77.8468°W), 363 m, QCAZ 73709 adult male. F. Ayala, D. Núñez, K. Nusirquia and A. Carvajal collected all specimens from 2018.

###### Definition.

In this section, coloration and characters refer to preserved specimens unless otherwise mentioned. The Definition and Diagnosis are based on eight adult males, females are unknown. *Hyloscirtuselbakyanae* sp. nov. can be diagnosed by the combination of the following characters: (1) male mean SVL 36.3 mm (range 34.5–37.6; *n* = 9; Suppl. material 1: table S5, Fig. 7); (2) white supralabial stripe absent; (3) tympanum round, inconspicuous; supratympanic fold inconspicuous and unpigmented; (4) white ulnar and cloacal folds present; white tarsal fold present, inconspicuous to conspicuous; (5) subarticular tubercles conspicuous in hands and feet; (6) supernumerary tubercles inconspicuous; (7) calcar tubercle absent; (8) pericloacal spots well-defined; (9) all surface plain cream with inconspicuous or absent white spots and minute or thick black spots or flecks in the dorsum; (10) in life, dorsal surfaces and flanks olive green, brownish green or greyish green with or without white flecks and with minute or thick black spots or flecks; axillar and inguinal regions silver or brownish; venter and posterior surfaces of tights yellow; other ventral surfaces silver or brownish; pericloacal spots ill-defined, unpigmented; webbing yellow orange; iris pearl to clam shell with pinkish drown or leather reticulations; (11) the advertisement call consists of a single note, with a mean duration of 0.056 ± 0.001 s and a dominant frequency of 2321.29 ± 127.86 Hz and fundamental frequency of 1184.35 ± 30.48 Hz. The call can be repeated in a series of 4–13 calls.

###### Diagnosis.

Characters in this section pertain to preserved specimens unless otherwise noticed. Coloration refers to live specimens. The most similar species to *Hyloscirtuselbakyanae* sp. nov. in the Amazon basin are *Hyloscirtusalbopunctulatus*, *H.maycu* sp. nov., *H.dispersus* sp. nov., *H.phyllognathus*, and *H.torrenticola. Hyloscirtuselbakyanae* sp. nov. differs by the absence of a white supralabial stripe (present in *H.albopunctulatus*, *H.phyllognathus* and *H.torrenticola*), an absent supratympanic fold (present in *H.albopunctulatus* and *H.dispersus* sp. nov.), an inconspicuous tarsal fold (conspicuous in *H.albopunctulatus*, *H.phyllognathus*, and *H.torrenticola*), conspicuous subarticular tubercles in hands and feet (inconspicuous in hands and feet in *H.albopunctulatus*, Fig. 11), inconspicuous supernumerary tubercles in hands and feet (conspicuous in hands in *H.dispersus* sp. nov.), absent pericloacal spots (present in *H.albopunctulatus* and *H.phyllognathus*), absent calcar tubercle (present in *H.dispersus* sp. nov. and *H.phyllognathus*), and a clam shell or pearl iris (clam shell with black or sand dune reticulations in *H.albopunctulatus* and a bronze iris in *H.torrenticola*, Fig. 12).

The advertisement call of *Hyloscirtuselbakyanae* sp. nov. has a rise time of 0.57 ± 0.05 s (longer in *H.albopunctulatus* with 2.28 ± 0.80 s and in *H.torrenticola* with 0.16 ± 0.017 s), a dominant frequency of 2321.29 ± 127.86 Hz and a fundamental frequency of 1184.35 ± 30.48 Hz (higher dominant frequency of 2795.41 ± 138.68 Hz and fundamental frequency of 2700.63 ± 195.66 Hz in *H.dispersus* sp. nov.). *Hyloscirtustorrenticola* also has a higher dominant frequency of 2743.79 ± 48.22 Hz and a fundamental frequency of 2743.75 ± 48.16 Hz. *Hyloscirtuselbakyanae* sp. nov. has a call duration of 0.06 ± 0.001 s (longer in *H.dispersus* sp. nov. with 0.11 ± 0.015 s) and an intercall duration of 0.12 ± 0.007 s (shorter in *H.torrenticola* with 0.07 ± 0.007 and longer in *H.albopunctulatus* with 0.30 ± 0.06 s and in *H.dispersus* sp. nov. of 0.33 ± 0.044 s) (Table 4, Fig. 9; Melin 1941; Duellman and Altig 1978; Rivera-Correa 2016).

Moreover, *H.elbakyanae* sp. nov. inhabits elevations between 214 and 622 m, while *H.maycu* sp. nov. lives higher between 882 and 1183 m, in colder and dryer environments (Figs 5 and 10). The available evidence indicates that *H.elbakyanae* sp. nov. is larger than *H.maycu* sp. nov. (Fig. 7).

###### Description of the holotype.

Description of characters based on preserved specimen. Adult male (Figs 18, 19). Measurements (in mm): SVL 36.1; foot length 14.3, head length 10.5, head width 11.7, eye diameter 3.4, tympanum diameter 1.6, tibia length 16.9, femur length 16.7, internarial distance 3.0, inter-orbital distance 5.5. Head wider than long; body slender; snout rounded in dorsal and lateral views; distance from nostril to eye shorter than diameter of eye; canthus rostralis distinct; loreal region concave; internarial region nearly flat; top of the head flat; nostrils not protuberant, round, directed anterolaterally; lips rounded, not flared; interorbital area slightly concave, longer than upper eyelid; tympanum inconspicuous, with upper and posterior margins barely covered by a curved and thin inconspicuous supratympanic fold reaching anterior margin of insertion of arm; tympanic annulus absent; tympanic membrane absent; mental gland present, oval-shaped, very distinct, extending ~ 1/2 the length of the head; dentigerous processes of vomers straight, in transverse row posterior to level of choanae, which is round, each process narrowly separated from each other and bearing 4 teeth; tongue slightly cordiform, widely attached to mouth floor; vocal slits and vocal sac present.

Forearms slender; axillary membrane absent; fingers bearing dermal fringes and rounded discs; relative lengths of fingers I < II < IV < III; webbing formula I basal II 2–—3– III 2^+^—2–IV; subarticular tubercles prominent, round, single; supernumerary tubercles inconspicuous; thenar and palmar tubercles absent; small prepollex, not modified as a spine; nuptial pads absent; ulnar tubercles absent; outer ulnar fold present. Hindlimbs moderately robust; toes bearing rounded discs; relative length of toes I < II < III < V < IV; extensive toe webbing, formula I 1–—1^3/4^ II 1–—2– III 1^+^—1– IV 1–—1– V; outer tarsal fold present; tarsal tubercles absent; calcar tubercle absent; subarticular tubercles round and single; supernumerary tubercles not distinctive; inner metatarsal tubercle present and ovoid, outer absent. Skin on dorsal surfaces and flanks smooth and ventral surfaces granular; cloacal opening directed posteriorly at upper level of thighs, rounded tubercles below; cloacal fold thick.

###### Color of holotype in preservative

(Fig. 18). Dorsal surfaces of the dorsum, flanks and limbs cream covered with minute black spots, more abundant in the head. Very few white spots barely visible dispersed on the posterior dorsum and dorsal surfaces of the hindlimbs. Venter, throat, and ventral surfaces of limbs plain cream. Mental gland cream with small black spots. White supralabial stripe absent. Ulnar, tarsal, and cloacal folds white. Webbing cream.

###### Color of holotype in life

(Fig. 19). Based on digital photographs. Dorsal surfaces and flanks dark brownish green with minute black spots, more abundant on the anterior part of the head and limbs, as if those areas were dark brown. Few barely visible white flecks spread on the hindlimbs. Venter and posterior surfaces of thighs yellow, other ventral surfaces whitish. Throat greenish white. Tympanum pale green. Mental gland calico. Ulnar, tarsal, and cloacal folds white. Webbing yellow. Iris pearl with pinkish-brown reticulations.

###### Variation.

Dorsal and ventral variation of adult specimens is illustrated on Figs 18, 19. In preservative, dorsum varies from cream with scattered white spots through the body and limbs and minute and thick black pots scattered across the body and limbs (e.g., QCAZ 73669) or barely visible black spots or marks (e.g., QCAZ 72666), without white spots and brownish black flecks dispersed in the body and limbs, more accumulated in the anterior part of the body (e.g., QCAZ 72668) or spots covering the entire body and limbs (e.g., QCAZ 72665) to paler cream without white spots and few black marks in the anterior part of the body (e.g., QCAZ 73709). Ventral surfaces vary from darker cream (e.g., QCAZ 72666) to paler cream (e.g., QCAZ 73709). Mental gland varies from cream (e.g., QCAZ 73669) to whitish (e.g., QCAZ 72665). White supralabial stripe varies from inconspicuous (e.g., QCAZ 72665) to absent (e.g., QCAZ 73709).

In life (Fig. 19), dorsum varies from pale olive green, olive green, brownish green, darker brownish green to greyish green with barely visible white spots scattered throughout the body and few black marks in the anterior part of the body (e.g., QCAZ 53831) or without black marks or spots (e.g., QCAZ 53807), to absent white or black spots (QCAZ 72665, 72667, 72669 and 73709). Ventral surfaces, besides venter and posterior thighs, vary from silver (e.g., QCAZ 53807) to whitish (e.g., QCAZ 73709). Iris varies from clam shell with thin reticulations (e.g., QCAZ 73709) to pearl with thicker reticulations (e.g., QCAZ 72667). White supralabial stripe varies from present (e.g., QCAZ 72665) to absent (e.g., QCAZ 72667).

###### Distribution and natural history.

*Hyloscirtuselbakyanae* sp. nov. is known from seven localities, nearby the type locality, Comunidad Shaime, Provincia Morona Santiago, Ecuador, at elevations between 214–622 m (Fig. 5). Biogeographic regions are Amazon Humid Tropical Forest and Eastern Lower Montane Forest (Ron et al. 2022). This species lives in hillside forest, with varying levels of anthropogenic disturbance. The habitat is dominated by palms (*Iriarteadeltoidea*) and trees up to 20–30 m high (collectors’ observations). They are nocturnal and have been found on ravines with shrub vegetation on the edge of torrent rivers and streams. *Hyloscirtuselbakyanae* sp. nov. calls from under rocks in streams with little water and cracks. There are no records of individuals perching on riparian vegetation.

###### Advertisement call.

We analyzed ten calls from two individuals. Both calls (QCAZ 53807–08) from Comunidad Shaime, Provincia Morona Santiago, recorded on 23 July 2012 by SRR and T. Camacho, water temperature 20–21 °C. The advertisement call consists of a single tonal note, repeated in series of 4–13 calls (Fig. 9C). Average call duration is 0.06 ± 0.05 s with an average inter-call interval of 0.12 ± 0.007 s. The average dominant frequency is 2321.29 ± 127.86. Other call parameters are listed in Table 4.

###### Conservation status.

The distribution polygon of *H.elbakyanae* sp. nov. is 11 km^2^. There is evidence of deforestation due to logging and it is not known to occur in protected areas. There might be undiscovered populations because the region where it occurs has not been thoroughly sampled. However, in response to its distribution range being less than 20000 km^2^ and having fewer than ten known localities, we propose assigning *H.elbakyanae* sp. nov. to the Red List category Vulnerable (VUB1abiii).

###### Etymology.

The specific name *elbakyanae* sp. nov. is a noun in the genitive case and is a patronym for Alexandra Elbakyan. She is a computer programmer and creator of Sci-Hub, a website which provides free access to scientific articles. Sci-Hub allows scientists worldwide to access articles that, otherwise, are behind paywalls and unaffordable in low- and middle-income countries. Our research has greatly benefited from access to relevant literature using Sci-Hub through the years.

##### ﻿ Hyloscirtus dispersus sp. nov.

Taxon classificationAnimaliaAnuraHylidae

43A76F83-26E6-5F84-81D2-F77878FB4876

https://zoobank.org/69BCDDF6-E41A-40E2-A389-44566337EC4C

Figs 5 , 7 , 9 , 10 , 11 , 20 , 21 , 22 Common name: Proposed standard English name: Dispersed stream frog Proposed standard Spanish name: Rana de Torrente dispersa

###### Type material.

**Figure 20.**
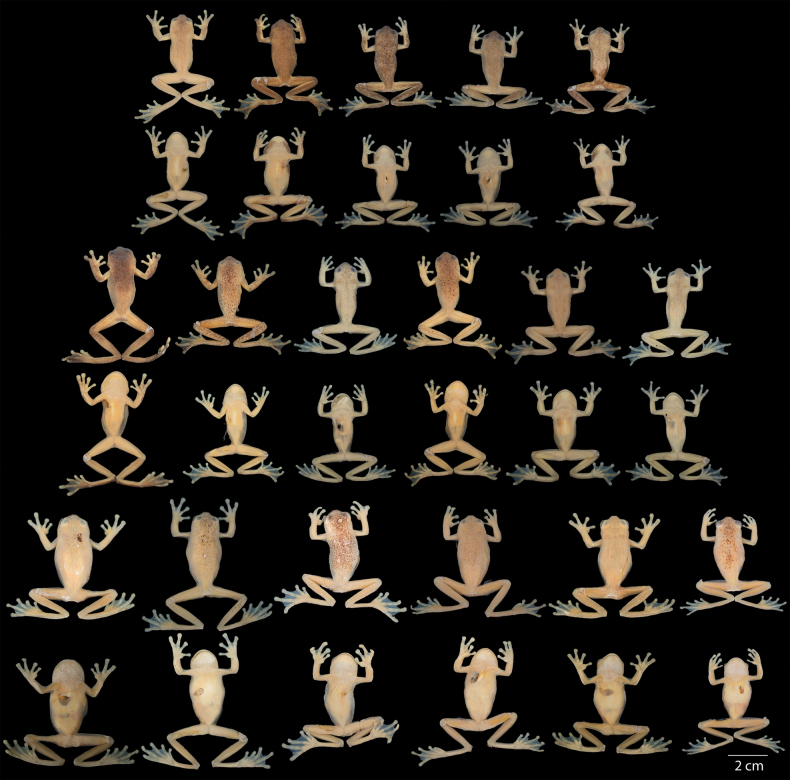
Variation of preserved specimens of *Hyloscirtusdispersus* sp. nov. Dorsal and ventral views. From left to right, first and second rows: QCAZ 52006 (holotype, adult male), QCAZ 41554, 69548, 69561, 66709 (adult males); third and fourth rows: QCAZ 66051, 52458, 66710, 66052, 69550, 63488 (adult males); fifth and six rows: QCAZ 60694, 57100, 41649, 41901, 60695, 41031 (adult females). See Suppl. material 1: table S1 for locality information. All specimens are shown at the same scale.

**Figure 21.**
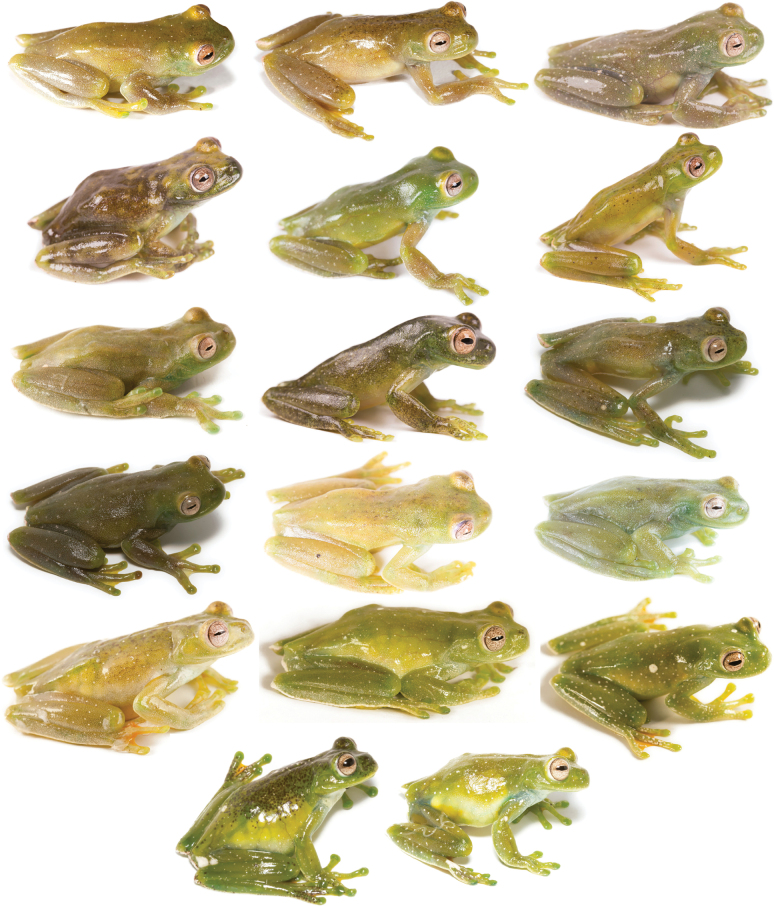
Variation in life of *Hyloscirtusdispersus* sp. nov. Dorsolateral view. From left to right, first row: QCAZ 52006 (holotype, adult male), 52463, 69562 (adult males); second row: QCAZ 66709, 59821, 66710 (adult males); third row: QCAZ 49032, 69563, 58732 (adult males); fourth row: QCAZ 58733, 63488, 69546 (adult males); fifth row: QCAZ 41031, 57100, 57666 (adult females); sixth row: QCAZ 60694, 60695 (adult females).

**Figure 22.**
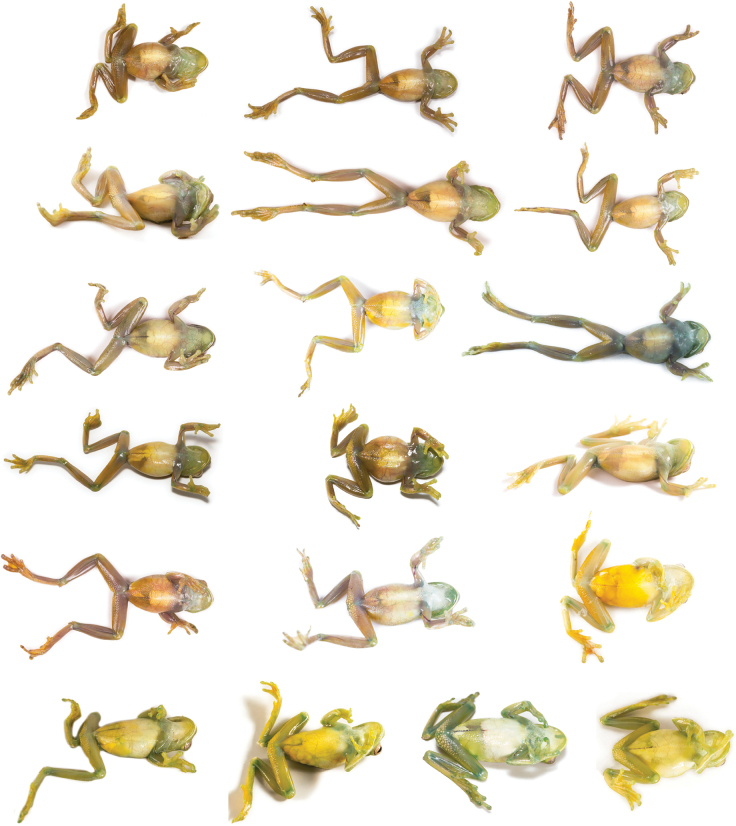
Variation in life of *Hyloscirtusdispersus* sp. nov. Ventral view. From left to right, first row: QCAZ 52006 (holotype, adult male), 52463, 69562 (adult males); second row: QCAZ 49032, 69563, 66709 (adult males); third row: QCAZ 59821, 66710, 58732 (adult males); fourth row: QCAZ 58733, 63408, 69456 (adult males); fifth row: QCAZ 69550, 59820 (adult males), QCAZ 41031 (adult female); sixth row: QCAZ 57099, 57666, 59099, 57100 (adult females).

***Holotype***. • QCAZ 52006 (Figs 20–22), field no. SC 38765, adult male from Ecuador, Provincia Tungurahua, Caserío Machay, 3 km E of Río Verde on the road to Puyo, (1.3923°S, 78.2801°W), 1349 m above sea level, collected by SRR, F. Ayala, T. Camacho, M. Yánez, D. Rivadeneira, S. Aldás and D. Pareja on 19 September 2011. A 3D model of the holotype is available at Sketchfab platform (https://skfb.ly/oSqqI). ***Paratypes***. • All collected in Ecuador. Provincia Sucumbíos: Río Azuela, Hostería El Reventador (0.0752°S, 77.5921°W), 1680 m, QCAZ 66709–10, adult males, collected by G. Vaca, M. Mejía and D. Escobar on 26 February 2017; • Provincia Napo: Cocodrilos, on Baeza–Archidona road (0.6710°S, 77.7928°W), 1575 m, QCAZ 63488, adult male, collected by SRR, S. Guamán, M.J. Navarrete, B. Proaño and A. Achig on 23 June 2016; • Provincia Tungurahua: same locality, date, and collectors as for the holotype, (1.4002°S, 78.2807°W), 1244 m, QCAZ 52007, adult male; • Reserva Río Zuñac (1.3765°S, 78.1540°W), 1594 m, QCAZ 52458, 52462, adult males, collected by F. Ayala, D. Paucar, Y. Sagredo, J.P. Reyes, F. Recalde, L. Recalde and S. Recalde on 16 January 2011; • Provincia Pastaza: Reserva Comunitaria Ankaku, on Puyo–Tena road (1.2676°S, 78.0479°W), 1668 m, QCAZ 46297, adult male, collected by E. Tapia on 15 October 2009; • Parque Nacional Llanganates, Comunidad Zarentza (0.3524°S, 78.072°W), 1419 m, QCAZ 59819–21, adult males, collected by D. Rivadeneira, F. Mora, J.C. Sánchez, D. Velalcázar, D. Núñez and J. Pinto; • Provincia Morona Santiago: Chiguinda (3.2278°S, 78.7200°W), 1741, QCAZ 18275, collected by Í. Tapia and G. Onore on 27 December 2001; • 16 km N El Ideal, on the road to Cuenca from Gualaquiza (3.2425°S, 78.6725°W), 1600 m, QCAZ 23936, metamorph, QCAZ 23937, 23945, adult males, collected by SRR and G. Romero on 09 April 2003; • 8.6 km E 9 de Octubre, Guamote–Macas road (2.24774°S, 78.2069°W), 1671 m, QCAZ 32267, adult male, collected by M. Bustamante, J. Guayasamin, E. Bonaccorso and J. F. Freile on 19 July 2006; • 4 km from Limón (Leonidas Plaza Gutiérrez), on the road to Plan de Milagro (2.9969°S, 78.4550°W), 1373–1409 m, QCAZ 40878, adult male, collected by I. Tapia, D. Salazar, L. Coloma and SRR on 07 June 2008, QCAZ 41901 adult female, collected by D. Salazar, E. Lemmon and A. Lemmon on 06 August 2008; • Limón (Leonidas Plaza Gutiérrez), Río Napinaza (2.9230°S, 78.4080°W), 1430 m, QCAZ 42002, adult male, collected by D. Salazar, E. Lemmon and A. Lemmon on 13 August 2008, QCAZ 42047, adult male, collected by D. Salazar and N. Peñafiel on 28 February 2008; • Bosque Protector Abanico (2.2448°S, 78.2053°W), 1646, QCAZ 49032, adult male, collected by Y. Sagredo and R. Jarrín on 26 July 2010; • 9 de Octubre–Macas road (2.2351°S, 78.2167°W), 1683 m, QCAZ 57014–16, adult males, collected by F. Ayala, Y. Sagredo, S. Arroyo, S. Valverde and L. Cedeño on 02 March 2014; • Parque Nacional Sangay, Sardinayacu (2.0928°S, 78.1687°W), 1475–1735 m, QCAZ 58732–3, 58735, adult males, collected by D. Rivadeneira, D. Velalcázar, J. Pinto, F. Mora, D. Núñez, J.C. Sanchez and A. Correa between 16 January 2015 and 26 January 2015, QCAZ 59099, adult female, collected by SRR, D. Paucar, PJV, P. Baldeón, M. Caminer and K. Nusirquia on 28 February 2015; • Puchimi (2.7774°S, 78.1595°W), 1365–1450 m, QCAZ 69548, 69550, 69555, 69561–63, adult males, collected by D. Almeida, D. Núñez, K. Nusirquia and J. Mora on 09 July 2017; • Comunidad Shuar Kunkuk, base of Cordillera del Cóndor mountain range (3.3302°S, 78.1972°W), 1521 m, QCAZ 71029, adult female, collected by D. Almeida, D. Núñez, K. Nusirquia and R. Gavilanes on 01 March 2018; • Cordillera de Cutucú, Carlos Hurtado’s house surroundings (2.7818°S, 78.1604°W), 1380 m, QCAZ 71428, adult male, collected by D. Almeida, D. Paucar, D. Núñez, K. Nusirquia and R. Gavilanes on 29 January 2018; • Provincia Zamora Chinchipe: Miazi Alto (4.2502°S, 78.6174°W), 1250 m, QCAZ 41031, adult female, 41032, male, collected by E. Tapia and J. Guayasamín on 12 April 2009, QCAZ 41554, adult male, collected by J. Guayasamín, E. Tapia and H. Braun on 07 April 2009, QCAZ 41649, adult female, collected by S. Aldás, J. Guayasamín and E. Tapia on 12 April 2009; • Los Encuentros (3.7568°S, 78.6457°W), QCAZ 47074, juvenile, 47110 female, collected by A. Almendáriz on 23 June 2009; Nuevo Paraíso, 700 m NO on the road to Las Tres Aguas (4.8710°S, 78.9757°W), 1742 m, QCAZ 57099–100, adult females, collected by D. Paucar, D. Almeida, G. Galarza and D. Pareja on 10 April 2014; • Reserva Numbami, 18 km on Zamora-Romerillos road (4.1760°S, 78.9561°W), 1434–1583 m, QCAZ 57664, 57667, adult males, 57665–66, adult females, collected by SRR, D. Paucar, PJV, D. Almeida, D. Velalcázar, M. J. Navarrete, S. Arroyo, N. Páez and Z. Lange on 11 July 2014; • Parque Nacional Podocarpus, Bombuscaro (4.1344°S, 78.9938°W), 1443 m, QCAZ 60688, 60692, adult males, 60694–95, adult females, collected by D. Rivadeneira, F. Mora, J. C. Sánchez, D. Velalcázar, D. Núñez, J. Pinto, K. Cruz and L. Tipantiza on 24 March 2015; • Concesión Mirador ECSA, Río Wawayme basin, towards Canales (3.59145°S, 78.4212°W), 1637 m, QCAZ 66050–52, adult males, collected by R. Betancourt, M. Cajamarca and L. Pandiguana on 23 November 2016; • Nuevo Paraíso, Ciudad Perdida (4.4803°S, 78.8294°W), 1334 m, QCAZ 68056, adult female, collected by F. Ayala, K. Nusirquia, D. Núñez and A. Calispa on 13 May 2017.

###### Definition.

In this section, coloration and characters refer to preserved specimens unless otherwise mentioned. The Definition and Diagnosis are based on 11 adult females and 36 adult males. *Hyloscirtusdispersus* sp. nov. can be diagnosed by the combination of the following characters: (1) mean SVL 34.1 mm in adult males (range 31.3–38.7; *n* = 36) and mean SVL 41.1 mm in adult females (range 35.4–45.2; *n* = 11; Suppl. material 1: table S5, Fig. 7); (2) white supralabial stripe present or absent; (3) tympanum rounded, inconspicuous to conspicuous in males and conspicuous in females; supratympanic fold present and unpigmented; (4) white ulnar and tarsal folds; (5) subarticular tubercles conspicuous in hands and feet; (6) supernumerary tubercles inconspicuous in feet and conspicuous in hands: (7) calcar tubercle present; (8) pericloacal spots well-defined; (9) all surfaces plain cream with a combination of scattered large or minute black spots and with white spots or flecks varying from ill-defined to large on the dorsum; (10) in life, dorsal surfaces and flanks yellowish green, olive green, dull green, brownish green or greyish green, with barely visible to thick white spots and minute or thick black spots or flecks, scattered throughout the body; venter yellow, gold, whitish, brownish green, grayish green, or dark pinkish; axillar and inguinal regions and ventral surfaces yellow, blueish, greenish, silver, brownish green or dark pinkish; pericloacal spots yellow, white or unpigmented; webbing yellow, yellow orange, whitish or dark pinkish; iris pearl or pinkish with leather reticulations; (11) the advertisement call consist of a single note, with a mean duration of 0.11 ± 0.015 s and a mean dominant frequency of 2795.4 ± 138.68 Hz and a fundamental frequency of 2700.63 ± 195.66 Hz. The call can be repeated in a series of 1 to 8 calls.

###### Diagnosis.

Characters in this section pertain to preserved specimens unless otherwise noticed. Coloration refers to life specimens. The most similar species to *Hyloscirtusdispersus* sp. nov. living in the Amazon basin are *Hyloscirtusalbopunctulatus* (sympatrically distributed, Fig. 5), *H.maycu* sp. nov., *H.elbakyanae* sp. nov., *H.phyllognathus*, and *H.torrenticola. Hyloscirtusdispersus* sp. nov. differs by having a supratympanic fold (absent in H.*maycu* sp. nov., *H.elbakyanae* sp. nov., *H.phyllognathus*, and *H.torrenticola*), an inconspicuous tarsal fold (thick in *H.albopunctulatus*, *H.phyllognathus*, and *H.torrenticola*), conspicuous subarticular tubercles in hands and fee (inconspicuous in hands and feet in *H.albopunctulatus*, Fig. 11), conspicuous supernumerary tubercles in hands (inconspicuous in hands and feet in *H.albopunctulatus*, *H.maycu* sp. nov., and *H.elbakyanae* sp. nov.), well-defined pericloacal spots (ill-defined or absent in *H.maycu* sp. nov. and *H.elbakyanae* sp. nov.), a calcar tubercle present (absent in all species except for *H.phyllognathus*), and an iris pearl or pinkish with leather-colored reticulations (clam shell with black or sand dune reticulations in *H.albopunctulatus* and a bronze iris in *H.torrenticola*, Fig. 12).

The advertisement call of *Hyloscirtusdispersus* sp. nov. differs by having a call duration of 0.11 ± 0.015 s (shorter in H.*albopunctulatus* with 0.051 ± 0.005 s and in *H.torrenticola* with 0.03 ± 0.001 s), a dominant frequency of 2743.79 ± 48.22 Hz (lower in *H.albopunctulatus* with 2149.84 ± 137.36 Hz and in *H.elbakyanae* sp. nov. with 2321.29 ± 127.86 Hz) and a fundamental frequency of 2700.63 ± 195.66 Hz (lower in *H.albopunctulatus* with 1214.12 ± 184.71 Hz and in *H.elbakyanae* sp. nov. with 1184.35 ± 30.48 Hz). *Hyloscirtusdispersus* sp. nov. has an intercall duration of 0.33 ± 0.044 s (shorter in *H.phyllognathus* with 0.06 s and in *H.torrenticola* with 0.07 ± 0.007 s) (Table 4, Fig. 9; Melin 1941; Duellman and Altig 1978; Rivera-Correa 2016). Moreover, all males of *H.dispersus* sp. nov. have been found calling while perching on vegetation over streams (*n* = 14; Pontificia Universidad Católica del Ecuador 2024), while all males of *H.albopunctulatus* were registered calling from under the rocks next to streams (*n* = 5; Pontificia Universidad Católica del Ecuador 2024).

Finally, *H.dispersus* sp. nov. inhabits elevations between 879 and 1807 m, while *Hyloscirtuselbakyanae* sp. nov. lives lower between 214 and 622 m and in warmer and wetter environments (Figs 5, 10).

###### Description of the holotype.

Description of characters based on preserved specimen. Adult male (Figs 20–22). Measurements (in mm): SVL 32.7; foot length 13.6, head length 9.6, head width 9.5, eye diameter 2.9, tympanum diameter 1.5, tibia length 15.8, femur length 14.3, internarial distance 2.7, interorbital distance 4.8. Head slightly longer than wide; body slender; snout rounded in dorsal view and slightly truncated in lateral view; distance from nostril to eye shorter than diameter of eye; canthus rostralis distinct, slightly convex; loreal region slightly concave; internarial region and top of the head flat; nostrils not protuberant, round, directed anterolaterally; lips rounded, not flared; interorbital area flat, longer than upper eyelid; tympanum round, with upper and posterior margins covered by a curved unpigmented supratympanic fold, reaching anterior margin of insertion of arm; tympanic annulus absent; tympanic membrane absent; mental gland present, oval-shaped, barely defined, extending ~ 1/3 the length of head; dentigerous processes of vomers straight, between round choanae, narrowly separated from each other, with five (right) and four (left) teeth; tongue slightly cordiform, widely attached to mouth floor; vocal slits and vocal sac present.

Forearms slender; axillary membrane absent; fingers bearing dermal fringes and rounded discs; relative lengths of fingers I < II < IV < III; webbing formula I basal II 2—3^+^ III 2^1/2^—2^+^ IV; subarticular tubercles prominent, round, single; supernumerary tubercles small; thenar tubercle absent, palmar tubercle small; prepollex present, not modified as a spine; nuptial pads absent; ulnar tubercles absent; outer ulnar fold present. Hindlimbs slender; toes bearing dermal fringes and rounded discs; relative length of toes I < II < III < V < IV; extensive toe webbing, formula I 2–—1^+^ II 1^+^—2– III 1^+^—2^+^ IV 2^+^—1– V; outer tarsal fold present; tarsal tubercles absent; calcar tubercle small, pinkish white; subarticular tubercles conspicuous, round and single; supernumerary tubercles inconspicuous in feet and conspicuous in hand; inner metatarsal tubercle present and ovoid, outer absent. Skin on dorsal surfaces and flanks smooth; venter finely granular; cloacal opening directed posteriorly at upper level of thighs, round tubercles below; cloacal fold present, thick.

###### Color of holotype in preservative

(Fig. 20). Dorsal surfaces of the head, body, limbs, and flanks cream densely covered with minute black spots, bigger black spots dispersed on the head and anterior part of the body. Very few, barely visible, white spots scattered on the posterior dorsum and hindlimbs. Venter, throat, and ventral surfaces of limbs cream. Mental gland cream. White supralabial stripe. White ulnar fold. Pinkish white cloacal and tarsal folds with black spots. Webbing cream.

###### Color of holotype in life

(Figs 21, 22). Based on digital photographs. Dorsal surfaces and flanks pale brownish green with minute black spots in the anterior part of the body and arms and white spots scattered throughout the body and limbs. Belly and other ventral surfaces are reddish brown. Tympanum and throat greenish. Mental gland yellowish green. Webbing reddish brown. Iris pinkish with leather reticulations.

###### Variation.

Dorsal and ventral variation of adult specimens is illustrated on Figs 20–22. In preservative (Fig. 20), dorsal background coloration varies from darker and brownish cream to pale cream. Background coloration has a variable pattern of white spots distributed throughout the body and limbs with minute black spots scattered in the body (e.g., QCAZ 41901), minute and thick brownish black spots (e.g., QCAZ 57100), few black marks scattered in the dorsum (e.g., QCAZ 60695) or without black spots (e.g., QCAZ 69550), to barely visible white spots in the body with minute black spots dispersed in the body (e.g., QCAZ 66710), minute and thick black or brownish black spots covering half the body or the entire body and limbs (e.g., QCAZ 41554, 69548), covering half the body (e.g., QCAZ 66052, 66709), with dark marks and flecks across the body and limbs (e.g., QCAZ 66051, 52458) and big white marks with thick brownish spots in the body (e.g., QCAZ 41649) and barely visible black spots (e.g., QCAZ 60694). Ventral surfaces vary from pale cream (e.g., QCAZ 57100) to cream (e.g., QCAZ 66051), without any pattern. Throat cream or whitish cream, with or without minute black spots (e.g., 66709). Mental gland varies from cream (e.g., QCAZ 69548) to whitish cream (e.g., QCAZ 41554). Cloacal fold varies from white (e.g., QCAZ 60695), pinkish white (e.g., QCAZ 69550), to pinkish white with black spots (e.g., QCAZ 69561). White supralabial stripe varies from absent (e.g., QCAZ 69548), inconspicuous (e.g., QCAZ 66051) to conspicuous (e.g., QCAZ 66710).

In life, dorsal background coloration varies from yellowish green, pale olive green, olive green, brownish green, darker brownish green, greyish green to lemon grass (Figs 21, 22). Background coloration has a variable pattern of white spots scattered throughout the body and limbs with minute and thick black spots or marks scattered across the body (e.g., QCAZ 52463, 58732), accumulated in the anterior part (e.g., QCAZ 60694), accumulated in the entire body (e.g., QCAZ 69563), with black flecks (e.g., QCAZ 41031) or without black spots or any marks (e.g., QCAZ 57100, 69546) to barely visible or absent white spots without any dark spots or marks (e.g., QCAZ 49032, 66710). Additionally, the dorsum can be covered by thick white marks (e.g., QCAZ 57666, 60694). Venter and posterior tights vary from yellow (e.g., QCAZ 41031, 57666 – less common), greenish (e.g., QCAZ 57099, 59099), lemon grass (e.g., QCAZ58732), brownish (e.g., QCAZ 52463, 69550), silver (e.g., QCAZ 59821) or white (e.g., QCAZ 69546). Ventral axillar and inguinal surfaces vary from yellow (e.g., QCAZ 41031) to green (e.g., QCAZ 59099) or white (e.g., QCAZ 69456). Throat white (e.g., QCAZ 41031), green (e.g., QCAZ 63488), or brownish (e.g., QCAZ 52463). White supralabial stripe varies from present (e.g., QCAZ 57100) to absent (e.g., QCAZ 66710). Iris varies from pearl with barely visible reticulations (e.g., QCAZ 69546) or leather reticulations (e.g., QCAZ 59821) to pinkish with leather reticulations (e.g., QCAZ 69563). Webbing matches ventral coloration.

###### Distribution and natural history.

*Hyloscirtusdispersus* sp. nov. is known from more than 25 localities from northern to southern Ecuador in the eastern Andean slopes, at elevations between 879–1807 m (Fig. 5). From the species analyzed in this study, this is the most widespread and its biogeographic regions are Eastern Lower Montane Forest and Eastern Montane Forest (Ron et al. 2022). This species lives in hillside forests, frequently found in secondary forest and artificial open areas. They are nocturnal and associated with streams of running water and ravines. Males call perched on riparian vegetation up to 2.5 m above the ground. It also occurs close to lagoons and small waterfalls. Perching sites include plants of Araceae, bromeliads, cedars, and ferns (Pontificia Universidad Católica del Ecuador 2024). This species has not been found living or calling under rocks. It is known to live in sympatry with *Hyloscirtusalbopunctulatus* and potentially with *H.maycu* sp. nov.

###### Advertisement call.

We analyzed 60 calls from 14 individuals. QCAZ 52006 from Caserío Machay, Provincia Tungurahua, 19 September 2011, recorded by SRR. QCAZ 59820 from Comunidad Zarentza, Llanganates National Park, Provincia Pastaza, 23 February 2015, air temperature 18 °C recorded by D. Rivadeneira. QCAZ 63488 from Cocodrilos, Provincia Napo, 23 June 2016, recorded by SRR. KU 164338 and one unvouchered specimen from 2 km SW of Río Reventador, Provincia Napo, 19 March 1975, temperature 18 °C, recorded by W. E. Duellman. USNM 286338, Río Reventador, Provincia Napo, recorded by R. McDiarmid. USNM 286349 from Baeza–Lago Agrio Road, Provincia Napo, 22 February 1985, recorded by R. McDiarmid. USNM 284316 from Cascada San Rafael, Provincia Napo, recorded by M. Foster. Two unvouchered specimens from San Rafael, Provincia Napo, recorded by R. McDiarmid. One unvouchered specimen from Sangay National Park, Provincia Morona Santiago, recorded by D. Batallas. Two unvouchered specimens from Río Azuela, Provincia Napo, 23 October 1971, temperature 18–19 °C, and one from Cordillera del Dué, Provincia Sucumbíos, recorded by W. Duellman. The advertisement call consists of a single note, repeated in series of 1–8 calls (Fig. 9E). Average call duration is 0.11 ± 0.015 s with an average inter-call interval of 0.33 ± 0.044 s. Mean dominant frequency is 2795.41 ± 138.68 Hz. Other call parameters are listed in Table 4.

###### Conservation status.

The distribution polygon of *H.dispersus* sp. nov. is 26,296 km^2^. Its distribution range overlaps with many protected areas. Its presence in secondary forests and artificial open areas indicates that it can withstand anthropogenic habitat change. Therefore, we propose assigning *H.dispersus* sp. nov. to the Red List category Least Concern.

###### Etymology.

The specific epithet comes from the Latin word *dispersus* in reference to the wide distribution range of this species, extending from north to south of the eastern Andes of Ecuador and probably with a wider unknown distribution that includes neighboring countries, Colombia, and Peru.

## ﻿Discussion

### ﻿Cryptic diversity

Our review of Amazonian species of the *Hyloscirtusbogotensis* species group, based on an integrative approach, resulted in the discovery of three new cryptic species. Prior to our review, the group only had three formally described Amazonian species. Therefore, the species described here represent a 100% increase in species content. The new species are visually cryptic as a result of shared patterns of highly variable dorsal coloration. However, some diagnostic morphological characters, genetic and bioacoustic data, and environmental conditions demonstrate that they represent evolutionary independent lineages.

Color is highly variable within the described species making morphology-based identification of closely related species challenging (see also Guayasamin et al. 2015). Our observations suggest that high variability is the result, in part, of phenotypic plasticity in coloration. Phenotypic plasticity refers to changes in the phenotype by a single genotype in different environments. We found two species of the *H.bogotensis* group with drastic coloration changes during short periods. One individual of *H.alytolylax*, for example, changed its dorsal coloration from greenish brown to greenish yellow, with scattered brown spots, within 16 minutes (Fig. 14C–E). We also found significant changes in *H.albopunctulatus* within periods of less than 20 days. Changes may have been faster, but we lacked photographs for shorter intervals. At least in *H.alytolylax*, color changes were likely physiological because they occurred within minutes. Physiological color plasticity is mediated by hormonal changes triggering intracellular mobilization of pigments (Duellman and Trueb 1994). A pending task is the assessment of the taxonomic extent of plastic color variation in *Hyloscirtus* and Hylidae, in general. Nevertheless, extensive intraspecific and intraindividual variation in dorsal coloration reinforce the notion of the unreliability of dorsal skin coloration in interspecies diagnosis.

As seen in other systematic reviews of Neotropical hylids (e.g., Funk et al. 2012; Rivadeneira et al. 2018), bioacoustic characters were one of the most useful phenotype components to diagnose closely related species. The most divergent calls were those of *H.dispersus* sp. nov. and *H.albopunctulatus* (sympatric species). The advertisement call of these species can be distinguished by ear and the parameters duration of the call and inter-call interval seem to play an important role. These differences, plus their preferred calling site, could help identify them in the field. Regarding morphology, some characters like iris coloration, tubercles and cloacal ornamentation can be useful for separating *H.albopunctulatus* from *H.dispersus* sp. nov. Other than size, we did not document morphological differences between *H.maycu* sp. nov. and *H.elbakyanae* sp. nov. However, they occur at different elevations and environments (dryer and colder for *H.maycu* sp. nov.) In addition to their high genetic distances (5.1% for gene 16S; Table 3), the haplotype network for the nuclear gene c-myc also supports their distinctiveness as they do not have shared haplotypes (Suppl. material 1: fig. S7). Our time tree indicates that they diverged over 5 Mya (Fig. 2).

*Hyloscirtustorrenticola* was not included in the phylogenetic or morphological analyses; however, the bioacoustic analysis, with calls from its type locality (Fig. 9F), show that this species is distinct from the geographically closer *H.albopunctulatus* and *H.dispersus* sp. nov. Because all known populations of the *H.bogotensis* group from northeastern Ecuador belong to either *H.albopunctulatus* or *H.dispersus* sp. nov., we propose that *H.torrenticola* is absent in Ecuador, at least until documented records become available. The report of *H.torrenticola* by Duellman and Altig (1978) from SW Río Reventador, Ecuador, likely corresponds to *H.dispersus* sp. nov. On the other hand, *Hyloscirtusphyllognathus* sensu stricto (Lineage E) occurs in Peru and is genetically divergent from the Ecuadorian species (see also Almendáriz et al. 2014).

Geographically, the Peruvian populations of *Hyloscirtusphyllognathus* sensu stricto are greatly distant (345 km) from southernmost Ecuadorian populations, and their bioacoustic and morphological space differ (Figs 5, 8, 9D). Based on this evidence, and the numerous Ecuadorian populations included in this study, we propose that *H.phyllognathus* sensu stricto does not occur in Ecuador and remains restricted to the Amazon basin of Peru. We do not know its actual distribution range, but excluding Ecuadorian populations greatly reduces its previous known range; however, extended sampling of northern Peruvian populations will provide better conclusions. Among all Amazonian species of the *Hyloscirtusbogotensis* group, only *H.dispersus* sp. nov., and *H.palmeri* maintain a large distribution range.

### ﻿Phylogenetic relationships and biogeographic history

*Hyloscirtus* monophyly is strongly supported, and four monophyletic species groups were recovered as previously reported (Rojas-Runjaic et al. 2018; Ron et al. 2018; Lyra et al. 2020). The phylogeny included 26 of the 40 described species of *Hyloscirtus*, one of the most comprehensive to date. Within the *Hyloscirtusbogotensis* species group, our results agreed with the latest phylogenies except for weakly supported nodes (Almendáriz et al. 2014; Guayasamin et al. 2015; Ron et al. 2018; Lyra et al. 2020). It should be noted that in all previous phylogenies of *Hyloscirtus* the identity of “*H.phyllognathus*” was mistaken and actually corresponds to *H.dispersus* sp. nov. – except for the individual KU212119, from Peru, which corresponds to *H.phyllognathus* sensu stricto (Lineage E, Fig. 1). The individual MZUTI192 identified as “H.aff.phyllognathus” by Guayasamin et al. (2015) actually corresponds to *H.albopunctulatus*.

While there were several limitations of our phylogenomic data generation and analyses (i.e., lower than expected UCE capture success, and four taxa with unexpected placements), the results of our preliminary analyses (Fig. 3, Suppl. material 1: figs S2–S4) are broadly consistent with the smaller molecular dataset. This includes strong support for the monophyly of *H.dispersus* sp. nov., *H.elbakyanae* sp. nov., and *H.albopunctulatus*, and the distinctiveness of one of the Peruvian lineages. Future scrutiny of this dataset is needed to clarify the four enigmatic taxa and issues associated with low-capture success. However, we interpret the general agreement with the smaller molecular dataset as evidence that many of the inferred phylogenetic relationships within the *Hyloscirtusbogotensis* species group are supported by widely distributed signal across the nuclear genome.

The lowest uncorrected *p*-genetic distance between species was 5.0% for the gene 16S (*H.maycu* sp. nov. and *H.elbakyanae* sp. nov.) and up to 15% (*Hyloscirtusalbopunctulatus* relative to the other Amazonian lineages). It has been suggested that distances > 3.0% for the gene 16S are indicative of separate species (e.g., Fouquet et al. 2007a). That threshold supports the description of the new species presented here, a result that is reinforced by our genomic, bioacoustic, and morphological datasets. Almendáriz et al. (2014) suggested that populations of *H.phyllognathus* of Ecuador and Peru represented separate species and we confirmed that hypothesis with our results. Moreover, we found 4% of genetic differentiation within Peruvian populations, suggesting the existence of one undescribed species. Guayasamin et al. (2015) mentioned a divergence of *H.phyllognathus* in Ecuador between north and south populations. However, according to our analysis, this divergence is < 1.8%, and we consider it intraspecific genetic variation. Overall, genetic distances should be interpreted with caution as a wide range of divergences have been observed both within and between species.

Our time tree agrees with the divergence time estimates by Portik et al. (2023), the most comprehensive time tree of Anura published to date. For example, Portik et al. (2023) estimate the divergence between the *H.bogotensis* group and its sister clade at 32.9 My, near our estimate of 28.5 My and within our 95% HPD, 21.6–35.0 My. Similarly, diversification within the *H.bogotensis* started at 27.9 My in Portik et al. (2023) vs. 23.8 My in our time tree and within our 5% HPD (16.64–30.80 My).

Our biogeographic analyses indicate that the barrier imposed by the high elevations of the Andes has played a crucial role in shaping the diversification of *Hyloscirtus*. Despite the ancient origin of the group (early Oligocene) we only found four trans-Andean colonization events between the Amazon and Pacific basins: three from east to west (Amazon to Pacific) and one from the Pacific to the Amazon. Therefore, communities of *Hyloscirtus* have evolved independently on opposite sides of the Andes since the mid-Miocene. The oldest colonization events for the genus (two events > 20 Mya) took place from the Amazon to the Caribbean basin. They occurred when most of the tropical Andes were below 3000 m (Fig. 2) and the hydrographic systems in South America were markedly different.

Species of the highland *Hyloscirtuslarinopygion* group were younger (average = 3.3 My) than those from lower elevations, the *Hyloscirtusbogotensis* group (7.8 My). This difference could be the result of the more recent origin of highland habitats as a result of Andean uplift during the last 5 My. A younger age for Andean species from higher elevations have been previously documented in plants (e.g., Hughes and Eastwood 2006). The most recent trans-Andean colonization event (~ 4 Mya) took place within the *H.larinopygion* group, a result consistent with its expected higher tolerance for cold climates relative to the lower elevation *H.bogotensis* species group. This scenario is supported by data on minimal critical temperature data (CT_min_) for both groups showing that tadpoles of the *H.larinopygion* group have CT_min_ ~ 4.5. °C lower than those of the *H.bogotensis* group (*H.alytolylax* CT_min_ = 6.3 °C; H.cf.albopunctulatus CT_min_ = 5.7 °C; vs. 1.5 in *H.lindae*; Pintanel et al. 2022). Thus, the timing of the colonization events across the Andes is consistent with the current elevation range and thermal tolerance of each group. Our biogeographic reconstructions are the first step in studying the genus’s origin and diversification history. Future studies can focus, for example, on the effects of barriers to gene flow or demographic modelling. This would allow for a more in-depth exploration of the biogeographic history and even comparisons with co-distributed genera.

Our environmental envelope analyses suggest the existence of environmental niche differences between closely related species of the *H.bogotensis* group. For example, *H.dispersus* sp. nov. occurs in drier environments relative to *H.elbakyanae* sp. nov. while *H.maycu* sp. nov. occurs in warmer environments than *H.albopunctulatus* (Fig. 10). Our analyses, however, are limited by the low number of known localities available for some species. We hope that the description of the new species will facilitate documenting new localities for the group and more comprehensive future analyses.

## ﻿Conclusions

Our results confirm that integrative approaches can help to assess the species boundaries of morphologically cryptic groups. Genetic and acoustic evidence played an important role in distinguishing species among the *Hyloscirtusbogotensis* group, letting us describe three new species of torrent frogs for the Amazon basin. Two of the new species are threatened with extinction, highlighting the need to protect Andean cloud forests. Our ancestral distribution analysis provides additional insights to understand the effect of geographic barriers in amphibian diversification in the Neotropics.

## Supplementary Material

XML Treatment for Hyloscirtus albopunctulatus

XML Treatment for Hyloscirtus maycu

XML Treatment for Hyloscirtus elbakyanae

XML Treatment for Hyloscirtus dispersus

## ﻿Additional information

### Author ORCIDs

Andrea Varela-Jaramillo https://orcid.org/0000-0002-0092-9915

Jeffrey W. Streicher https://orcid.org/0000-0002-3738-4162

Pablo J. Venegas https://orcid.org/0000-0002-6501-4492

Santiago R. Ron https://orcid.org/0000-0001-6300-9350

### Data availability

All of the data that support the findings of this study are available in the main text, Supplementary Information, and the repository Zenodo.org.

## Supplementary materials

Supplementary material 1 Additional information Data type doc Explanation note **table S1.** Taxon, voucher number, locality and GenBank data of the species included in the Sanger-based phylogenetic analysis. **table S2.** Details of the preferred partitions found with their best substitution models under Partition Finder and Model Find searches. **table S3.** Sequences for complete gene 16S, obtained from GenBank and amplified in this study. **table S4.** Examined specimens for the morphological analysis include the voucher number, sex of the specimen and the hosting museum. **table S5.** Measurements (in mm) of specimens of *Hyloscirtus*. **table S6.** Character loadings and eigenvalues for Principal Components (PC I, II and III) of the morphology analysis. **table S7.** Character loadings and eigenvalues for Principal Components (PC I–II) of the acoustic analysis. **table S8.** Character loadings and eigenvalues for Principal Components (PC I–II) of the environmental analysis. **table S9.** Additional specimens examined. **fig. S1.** Map showing the current distribution range known for *H.albopunctulatus*, *H.phyllognathus* and *H.torrenticola* before this study. **fig. S2.** Consensus tree obtained under Maximum likelihood criterion when using taxa with more than 300 UCE loci enriched. **fig. S3.** Consensus tree obtained under Maximum likelihood criterion when using taxa with more than 200 UCE loci enriched. **fig. S4.** Consensus tree obtained under Maximum likelihood criterion when using taxa with more than 300 UCE loci enriched. **fig. S5.** Species delimitation tree based on the Poisson Tree Process (PTP) criteria, using the 12S mitochondrial gene. **fig. S6.** Plot depicting the number of species recovered by the ABGD criteria as a function of the prior for intraspecific genetic divergences. **fig. S7.** Haplotype network for DNA sequences of 59 individuals for the nuclear gene C-myc. https://binary.pensoft.net/file/1279281This dataset is made available under the Open Database License (http://opendatacommons.org/licenses/odbl/1.0/). The Open Database License (ODbL) is a license agreement intended to allow users to freely share, modify, and use this Dataset while maintaining this same freedom for others, provided that the original source and author(s) are credited.Andrea Varela-Jaramillo, Jeffrew W. Streicher, Pablo J. Venegas, Santiago R. Ron
