# Supplementary material for: ﻿Three new species of torrent treefrogs (Anura, Hylidae) of the Hyloscirtusbogotensis group from the eastern Andean slopes and the biogeographic history of the genus
Source: Zookeys. 2025 Mar 13;1231:233–92. doi: 10.3897/zookeys.1231.124926 (PMC11926613; doi:10.3897/zookeys.1231.124926)
Supplement: Supplementary material 1 — Additional information [file zookeys-1231-233_article-124926__-s001.doc]

**SUPPLEMENTARY INFORMATION**

**Supplementary Tables**

**Table S1.** **Taxon, voucher number, locality and GenBank data of the species included in the Sanger-based phylogenetic analysis. The asterisks (*) represent the individuals also included in the genomic analysis (UCEs).**

| **Species** | **Museum Number** | **Locality** | **Gen bank Accession Number** | | | | **Source** |
| --- | --- | --- | --- | --- | --- | --- | --- |
|  |  |  | **12S** | **ND1** | **c-myc** | **RAG-1** |  |
| *Hyloscirtus albopunctulatus* | MZUTI  192 | Ecuador: Napo: Stream in the Chaco-Lago Agrio Road (0.0995°S, 77.5840°W, 1243 m) | KT279522 | - | - | - | Guayasamin et al. 2015 |
| *Hyloscirtus albopunctulatus* | QCAZ  46447* | Ecuador: Morona Santiago: Nuevo Israel (2.1650°S, 77.9029°W, 1289 m) | - | PQ893113 | PQ894757 | - | This study |
| *Hyloscirtus albopunctulatus* | QCAZ  48254* | Ecuador: Napo: Archidona-Baeza road, Lucian river (0.9180°S, 77.7920°W, 647 m) | PP482858 | PQ893117 | PQ894761 | PQ898865 | This study |
| *Hyloscirtus albopunctulatus* | QCAZ  48503* | Ecuador: Orellana: Rio Bigal Biological Reserve (0.5251°S, 77.4172°S, 944 m) | PP482859 | PQ893118 | PQ894762 | PQ898866 | This study |
| *Hyloscirtus albopunctulatus* | QCAZ  54111 | Ecuador: Pastaza: K10 camp, Campo Villano (1.4739°S, 77.5310°W) | PP482867 | PQ893126 | PQ894770 | - | This study |
| *Hyloscirtus albopunctulatus* | QCAZ 59809 | Ecuador: Pastaza: Zarentza Community, Llanganates National Park (1.3543°S, 78.0620°W, 1388 m) | PP482879 | PQ893138 | PQ894782 | PQ898881 | This study |
| *Hyloscirtus albopunctulatus* | QCAZ 59810 | Ecuador: Pastaza: Zarentza Community, Llanganates National Park (1.3543°S, 78.0620°W, 1388 m) | PP482880 | PQ893139 | PQ894783 | PQ898882 | This study |
| *Hyloscirtus albopunctulatus* | QCAZ  59813* | Ecuador: Pastaza: Zarentza Community, Llanganates National Park (1.3543°S, 78.0620°W, 1388 m) | PP482881 | PQ893140 | PQ894784 | - | This study |
| *Hyloscirtus albopunctulatus* | QCAZ  59814* | Ecuador: Pastaza: Zarentza Community, Llanganates National Park (1.3626°S, 78.0582°W, 1350 m) | PP482882 | PQ893141 | PQ894785 | PQ898883 | This study |
| *Hyloscirtus albopunctulatus* | QCAZ 59815* | Ecuador: Pastaza: Llanganates National Park (1.3626°S, 78.0582°W, 1350 m) | PP482883 | PQ893142 | PQ894786 | PQ898884 | This study |
| *Hyloscirtus albopunctulatus* | QCAZ  59816* | Ecuador: Pastaza: Zarentza Community, Llanganates National Park (1.3570°S, 78.0581°W, 1354 m) | PP482884 | PQ893143 | PQ894787 | PQ898885 | This study |
| *Hyloscirtus albopunctulatus* | QCAZ  59817* | Ecuador: Pastaza: Zarentza Community, Llanganates National Park (1.3570°S, 78.0581°W, 1354 m) | PP482885 | PQ893144 | PQ894788 | PQ898886 | This study |
| *Hyloscirtus albopunctulatus* | QCAZ  59822* | Ecuador: Pastaza: Zarentza Community, Llanganates National Park (1.3524°S, 78.0756°W, 1419 m) | PP482888 | PQ893147 | PQ894791 | PQ898889 | This study |
| *Hyloscirtus albopunctulatus* | QCAZ  59823* | Ecuador: Pastaza: Zarentza Community, Llanganates National Park (1.3397°S, 78.0594°W, 1360 m) | PP482889 | PQ893148 | PQ894792 | PQ898890 | This study |
| *Hyloscirtus albopunctulatus* | QCAZ  59825 | Ecuador: Pastaza: Zarentza Community, Llanganates National Park (1.3397°S, 78.0594°W, 1360 m) | PP482890 | PQ893149 | PQ894793 | PQ898891 | This study |
| *Hyloscirtus albopunctulatus* | QCAZ  59826* | Ecuador: Pastaza: Zarentza Community, Llanganates National Park (1.3626°S, 78.0582°W, 1350 m) | PP482891 | PQ893150 | PQ894794 | PQ898892 | This study |
| *Hyloscirtus albopunctulatus* | QCAZ  59827* | Ecuador: Pastaza: Zarentza Community, Llanganates National Park (1.3625°S, 78.0578°W, 1391 m) | PP482892 | PQ893151 | PQ894795 | PQ898893 | This study |
| *Hyloscirtus albopunctulatus* | QCAZ  59828* | Ecuador: Pastaza: Zarentza Community, Llanganates National Park (1.3603°S, 78.0611°W, 1340 m) | PP482893 | PQ893152 | PQ894796 | PQ898894 | This study |
| *Hyloscirtus albopunctulatus* | QCAZ 62188 | Ecuador: Pastaza: Kurintza Community, Villano camp (1.5059°N, 77.5096°W, 389 m) | PP482837 | PQ893095 | PQ894738 | PQ898855 | This study |
| *Hyloscirtus alytolylax* | MZUTI 409 | Ecuador: Pichincha: Las Gralarias Reserve, Lucy’s creek (0.0040°S, 78.7400°W, 1822 m) | KT279504 | - | - | - | Guayasamin et al. 2015 |
| *Hyloscirtus alytolylax* | QCAZ  24376 | Ecuador: Cotopaxi: San Francisco de las Pampas, Quebrada Santa Elena (0.4333°S, 78.9666°W, 1760 m) | X155799 | - | - |  | Coloma et al. 2012 |
| *Hyloscirtus alytolylax* | QCAZ  40606* | Ecuador: Cotopaxi: La Envidia, Correra Sapanal, segunda cascada (1.0097°S, 79.2376°W, 414 m) | PP482844 | PQ893102 | PQ894745 | - | This study |
| *Hyloscirtus alytolylax* | QCAZ  51332* | Ecuador: Bolivar: Telimbela puente (1.6675°S, 79.1705°W, 1063 m) | PP482860 | PQ893119 | PQ894763 | PQ898867 | This study |
| *Hyloscirtus armatus* | AMNHA 165163 | Bolivia: Santa Cruz: San Juan. Amboro National Park | AY549321 | KF794111 | - | - | Faivovich et al. 2004 |
| *Hyloscirtus callipeza* | UIS-A 5947 | Colombia: Santander: Vereda Vegas del Quemado | MG596780 | - | - | - | Rojas-Runjaic et al. 2018 |
| *Hyloscirtus charazani* | AMNHA 165132 | Bolivia: La Paz: Charazani | AY843618 | KF794112 | - | - | Faivovich et al. 2005 |
| *Hyloscirtus colymba* | SIUC H-7079 | Panama: Cocle: El Cope National Park | AY843620 | KF794113 | - | - | Faivovich et al. 2005 |
| *Hyloscirtus condor* | MEPN 14754 | Ecuador: Zamora Chinchipe: Reserva Biológica Cerro Plateado (4.6044°S, 78.8210°fW, 2317 m) | KF756939 | - | - | - | Almendáriz et al. 2014 |
| *Hyloscirtus criptico* | QCAZ 43422* | Ecuador: Imbabura: near Cuellaje, San Antonio (0.4747°N, 78.5550°W, 2560 m) | JX155814 | - | - | - | Coloma et al. 2012 |
| *Hyloscirtus dispersus*  sp. nov. | MZUTI  1353 | Ecuador: Napo: Cordillera de los Guacamayos (0.9354°S, 77.7930°W, 1243 m) | KT279521 | - | - | - | Guayasamin et al. 2015 |
| *Hyloscirtus dispersus*  sp. nov. | MZUTI 1354 | Ecuador: Napo: Cordillera de los Guacamayos (0.9354°S, 77.7930°W, 1243 m) | KT279515 | - | - | - | Guayasamin et al. 2015 |
| *Hyloscirtus dispersus*  sp. nov. | MZUTI 2383 | Ecuador: Napo: Stream in the Chaco–Lago Agrio road (0.0995° S, 77°5840° W; 1243 m) | KT279518 | - | - | - | Guayasamin et al. 2015 |
| *Hyloscirtus dispersus*  sp. nov. | MZUTI 2384 | Ecuador: Napo: Stream in the Chaco–Lago Agrio road (0.0995° S, 77°5840° W; 1243 m) | KT279519 | - | - | - | Guayasamin et al. 2015 |
| *Hyloscirtus dispersus*  sp. nov. | MZUTI 2385 | Ecuador: Napo: Stream in the Chaco–Lago Agrio road (0.0995° S, 77°5840° W; 1243 m) | KT279517 | - | - | - | Guayasamin et al. 2015 |
| *Hyloscirtus dispersus*  sp. nov. | MZUTI 2386 | Ecuador: Napo: Stream in the Chaco–Lago Agrio road (0.0995° S, 77°5840° W; 1243 m) | KT279516 | - | - | - | Guayasamin et al. 2015 |
| *Hyloscirtus dispersus*  sp. nov. | QCAZ  18275* | Ecuador: Morona Santiago: Chiguinda (3.2270°S, 78.7200°W, 1741m) | PP482838 | PQ893096 | PQ894739 | - | This study |
| *Hyloscirtus dispersus*  sp. nov. | QCAZ  23936 | Ecuador: Morona Santiago: 16 km M El Ideal, Cuenca road (3.2426°S, 78.6725°W, 1600 m) | PP482839 | PQ893097 | PQ894740 | - | This study |
| *Hyloscirtus dispersus*  sp. nov. | QCAZ  23937* | Ecuador: Morona Santiago: 16 km M El Ideal, Cuenca road (3.2426°S, 78.6725°W, 1600 m) | PP482840 | PQ893098 | PQ894741 | - | This study |
| *Hyloscirtus dispersus*  sp. nov. | QCAZ  23945* | Ecuador: Morona Santiago: 16 km M El Ideal, Cuenca road (3.2426°S, 78.6725°W, 1600 m) | PP482841 | PQ893099 | PQ894742 | - | This study |
| *Hyloscirtus dispersus*  sp. nov. | QCAZ  32267 | Ecuador: Morona Santiago: Nueve de Octubre (2.2445°S, 78.2069°W, 1671m) | PP482842 | PQ893100 | PQ894743 | - | This study |
| *Hyloscirtus dispersus*  sp. nov. | QCAZ 32271 | Ecuador: Morona Santiago: ca. Nueve de Octubre (2.24453°S, 78.20747°W, 1527 m) | PP482843 | PQ893101 | PQ894744 | - | Coloma et al. 2012  This study |
| *Hyloscirtus dispersus*  sp. nov. | QCAZ  40978* | Ecuador: Morona Santiago: 4 km Limón, Plan de Milagro road (2.9970°S, 78.4550°W, 1409 m) | - | PQ893103 | PQ894746 | - | This study |
| *Hyloscirtus dispersus*  sp. nov. | QCAZ  41031 | Ecuador: Zamora Chinchipe: Miazi Alto (4.2502°S, 78.6174°W, 1250 m) | PP482845 | - | PQ894747 | - | This study |
| *Hyloscirtus dispersus*  sp. nov. | QCAZ  41032* | Ecuador: Zamora Chinchipe: Miazi Alto (4.2502°S, 78.6174°W, 1250 m) | PP482846 | PQ893104 | PQ894748 | PQ898856 | Coloma et al. 2012  This study |
| *Hyloscirtus dispersus*  sp. nov. | QCAZ  41554 | Ecuador: Zamora Chinchipe: Miazi Alto (4.2502°S, 78.6174°W, 1250 m) | PP482847 | PQ893105 | PQ894749 | PQ898857 | This study |
| *Hyloscirtus dispersus*  sp. nov. | QCAZ  41649* | Ecuador: Zamora Chinchipe: Miazi Alto (4.2502°S, 78.6174°W, 1250 m) | PP482848 | PQ893106 | PQ894750 | PQ898858 | This study |
| *Hyloscirtus dispersus*  sp. nov. | QCAZ  41901* | Ecuador: Morona Santiago: Gral. Leónidas Plaza Gutiérrez (2.9962°S, 78.4558°W, 1373 m) | PP482849 | PQ893107 | PQ894751 | PQ898859 | This study |
| *Hyloscirtus dispersus* sp. nov. | QCAZ  41951* | Ecuador: Morona Santiago: Gral. Leónidas Plaza Gutiérrez, Napinaza river (2.9230°S, 78.4080°W, 1430 m) | - | - | - | - | This study |
| *Hyloscirtus dispersus*  sp. nov. | QCAZ  42002 | Ecuador: Morona Santiago: Gral. Leónidas Plaza Gutiérrez, Napinaza river (2.9230°S, 78.4080°W, 1430 m) | PP482850 | PQ893108 | PQ894752 | PQ898860 | This study |
| *Hyloscirtus dispersus*  sp. nov. | QCAZ  42047* | Ecuador: Morona Santiago: Gral. Leónidas Plaza Gutiérrez, Napinaza river (2.9230°S, 78.4080°W, 1430 m) | PP482851 | PQ893109 | PQ894753 | PQ898861 | This study |
| *Hyloscirtus dispersus*  sp. nov. | QCAZ  45679* | Ecuador: Napo: Pacto Sumaco, Pabayacu Refuge (0.5639°S, 77.6154°W, 2775 m) | PP482852 | PQ893110 | PQ894754 | PQ898862 | This study |
| *Hyloscirtus dispersus*  sp. nov. | QCAZ  46297 | Ecuador: Pastaza: Private Reserve Ankaku, Challuwa Yacu river (1.2676°S, 78.0479°W, 1668 m) | PP482854 | PQ893112 | PQ894756 | - | This study |
| *Hyloscirtus dispersus*  sp. nov. | QCAZ  47074 | Ecuador: Zamora Chinchipe: Los Encuentros, Colibrí (3.7568°S, 78.6457°W, 879 m) | PP482855 | PQ893114 | PQ894758 | PQ898863 | This study |
| *Hyloscirtus dispersus*  sp. nov. | QCAZ  47110* | Ecuador: Zamora Chinchipe: Los Encuentros, Colibrí (3.7568°S, 78.6457°W, 879 m) | PP482856 | PQ893115 | PQ894759 | PQ898864 | This study |
| *Hyloscirtus dispersus*  sp. nov. | QCAZ  52006* | Ecuador: Tungurahua: Caserío Machay, 3 km E Rio Verde (1.3923°S, 78.2801°W, 1349 m) | PP482861 | PQ893120 | PQ894764 | - | This study |
| *Hyloscirtus dispersus*  sp. nov. | QCAZ  52007* | Ecuador: Tungurahua: Caserío Machay, 3 km E Rio Verde (1.3923°S, 78.2801°W, 1349 m) | PP482862 | PQ893121 | PQ894765 | PQ898868 | This study |
| *Hyloscirtus dispersus*  sp. nov. | QCAZ  52458* | Ecuador: Tungurahua: Rio Zuñac Reserve (1.3765°S, 78.1540°W, 1594 m) | PP482863 | PQ893122 | PQ894766 | - | This study |
| *Hyloscirtus dispersus*  sp. nov. | QCAZ  52462* | Ecuador: Tungurahua: Rio Zuñac Reserve (1.3765°S, 78.1538°W, 1600 m) | PP482864 | PQ893123 | PQ894767 | PQ898869 | This study |
| *Hyloscirtus dispersus*  sp. nov. | QCAZ  57015 | Ecuador: Morona Santiago: Nueve de Octubre-Macas road (2.2351°S, 78.2167°W, 1683 m) | PP482868 | PQ893127 | PQ894771 | - | This study |
| *Hyloscirtus dispersus*  sp. nov. | QCAZ  57016* | Ecuador: Morona Santiago: Nueve de Octubre-Macas road (2.2352°S, 78.2167°W, 1688 m) | PP482869 | PQ893128 | PQ894772 | PQ898872 | This study |
| *Hyloscirtus dispersus*  sp. nov. | QCAZ  57099* | Ecuador: Zamora Chinchipe: Nuevo Paraíso, 700 m NO Las Tres Aguas (4.8710°S, 78.9757°W, 1742 m) | PP482870 | PQ893129 | PQ894773 | PQ898873 | This study |
| *Hyloscirtus dispersus*  sp. nov. | QCAZ  57100 | Ecuador: Zamora Chinchipe: Nuevo Paraíso, 700 m NO Las Tres Aguas (4.8710°S, 78.9757°W, 1742 m) | PP482871 | PQ893130 | PQ894774 | PQ898874 | This study |
| *Hyloscirtus dispersus*  sp. nov. | QCAZ  57664 | Ecuador: Zamora Chinchipe: Numbami Reserve, 18 km Zamora-Romerillos road (4.1760°S, 78.9561°W, 1464 m) | PP482872 | PQ893131 | PQ894775 | PQ898875 | This study |
| *Hyloscirtus dispersus*  sp. nov. | QCAZ  57665* | Ecuador: Zamora Chinchipe: Numbami Reserve, 18 km Zamora-Romerillos road (4.1756°S, 78.9562°W, 1434 m) | PP482873 | PQ893132 | PQ894776 | PQ898876 | This study |
| *Hyloscirtus dispersus*  sp. nov. | QCAZ  57666* | Ecuador: Zamora Chinchipe: Numbami Reserve, 18 km Zamora-Romerillos road (4.1832°S, 78.9604°W, 1583 m) | PP482874 | PQ893133 | PQ894777 | PQ898877 | This study |
| *Hyloscirtus dispersus*  sp. nov. | QCAZ  57667 | Ecuador: Zamora Chinchipe: Numbami Reserve, 18 km Zamora-Romerillos road (4.1807°S, 78.9599°W, 1583 m) | PP482875 | PQ893134 | PQ894778 | - | This study |
| *Hyloscirtus dispersus*  sp. nov. | QCAZ  58732 | Ecuador: Morona Santiago: Sardinayacu, Sangay National Park (2.0928°S, 78.1687°W, 1475 m) | PP482876 | PQ893135 | PQ894779 | PQ898878 | This study |
| *Hyloscirtus dispersus*  sp. nov. | QCAZ  58735* | Ecuador: Morona Santiago: Sardinayacu, Sangay National Park (2.0721°S, 78.2182°W, 1735 m) | PP482877 | PQ893136 | PQ894780 | PQ898879 | This study |
| *Hyloscirtus dispersus*  sp. nov. | QCAZ  59099* | Ecuador: Morona Santiago: Sardinayacu, Sangay National Park (2.0757°S, 78.2157°W, 1724 m) | PP482878 | PQ893137 | PQ894781 | PQ898880 | This study |
| *Hyloscirtus dispersus*  sp. nov. | QCAZ  59819* | Ecuador: Pastaza: Zarentza Community, Llanganates National Park (1.3523°S, 78.0597°W, 1419 m) | PP482829 | - | - | - | This study |
| *Hyloscirtus dispersus*  sp. nov. | QCAZ  59820* | Ecuador: Pastaza: Zarentza Community, Llanganates National Park (1.3523°S, 78.0597°W, 1419 m) | PP482886 | PQ893145 | PQ894789 | PQ898887 | This study |
| *Hyloscirtus dispersus*  sp. nov. | QCAZ 59821 | Ecuador: Pastaza: Zarentza Community, Llanganates National Park (1.3524°S, 78.0756°W, 1419 m) | PP482887 | PQ893146 | PQ894790 | PQ898888 | This study |
| *Hyloscirtus dispersus*  sp. nov. | QCAZ  60688 | Ecuador: Zamora Chinchipe: Bombuscaro, Podocarpus National Park (4.1344°S, 78.9938°W, 1443 m) | PP482895 | PQ893154 | PQ894798 | PQ898896 | This study |
| *Hyloscirtus dispersus*  sp. nov. | QCAZ  60692* | Ecuador: Zamora Chinchipe: Bombuscaro, Podocarpus National Park (4.1344°S, 78.9938°W, 1443 m) | PP482896 | PQ893155 | PQ894799 | PQ898897 | This study |
| *Hyloscirtus dispersus*  sp. nov. | QCAZ  60694* | Ecuador: Zamora Chinchipe: Bombuscaro, Podocarpus National Park (4.1344°S, 78.9938°W, 1443 m) | PP482897 | PQ893156 | PQ894800 | - | This study |
| *Hyloscirtus dispersus*  sp. nov. | QCAZ  60695 | Ecuador: Zamora Chinchipe: Bombuscaro, Podocarpus National Park (4.1344°S, 78.9938°W, 1443 m) | PP482898 | PQ893157 | PQ894801 | PQ898898 | This study |
| *Hyloscirtus dispersus*  sp. nov. | QCAZ 63488 | Ecuador: Napo: Cocodrilos, Baeza-Archidona road (0.6666°S, 77.7938°W, 1602 m) | PP482900 | PQ893159 | PQ894803 | PQ898899 | This study |
| *Hyloscirtus dispersus*  sp. nov. | QCAZ 66050 | Ecuador: Zamora Chinchipe: Concesión Mirador ECSA, Río Wawayme basin, towards Canales (3.5914°S, 78.4212°W, 1637 m) | PP482901 | PQ893160 | PQ894804 | PQ898900 | This study |
| *Hyloscirtus dispersus*  sp. nov. | QCAZ 66051 | Ecuador: Zamora Chinchipe: Concesión Mirador ECSA, Río Wawayme basin, towards Canales (3.5914°S, 78.4212°W, 1637 m) | PP482902 | PQ893161 | PQ894805 | PQ898901 | This study |
| *Hyloscirtus dispersus*  sp. nov. | QCAZ 66052 | Ecuador: Zamora Chinchipe: Concesión Mirador ECSA, Río Wawayme basin, towards Canales (3.5914°S, 78.4212°W, 1637 m) | PP482903 | PQ893162 | PQ894806 | PQ898902 | This study |
| *Hyloscirtus dispersus*  sp. nov. | QCAZ 66709 | Ecuador: Sucumbíos: Hostería El Reventador (0.0752°S, 77.5921°W, 1807 m) | PP482904 | - | PQ894807 | - | This study |
| *Hyloscirtus dispersus*  sp. nov. | QCAZ 66710 | Ecuador: Sucumbíos: Hostería El Reventador (0.0752°S, 77.5921°W, 1807 m) | PP482905 | - | PQ894808 | PQ898903 | This study |
| *Hyloscirtus dispersus*  sp. nov. | QCAZ 68056 | Ecuador. Zamora Chinchipe: Camp besides Río Nangaritza, trail from Ciudad Perdida to camp (4,4803°S, 78,8294°W, 1334 m) | PP482912 | PQ893169 | PQ894815 | PQ898910 | This study |
| *Hyloscirtus dispersus*  sp. nov. | QCAZ 69548 | Ecuador: Morona Santiago: Puchimi (2.7774°S, 78.1595°W, 1401 m) | PP482913 | PQ893170 | PQ894816 | PQ898911 | This study |
| *Hyloscirtus dispersus*  sp. nov. | QCAZ 69550 | Ecuador: Morona Santiago: Puchimi (2.7774°S, 78.1595°W, 1369 m) | PP482914 | PQ893171 | PQ894817 | PQ898912 | This study |
| *Hyloscirtus dispersus*  sp. nov. | QCAZ 69555 | Ecuador: Morona Santiago: Puchimi (2.7774°S, 78.1595°W, 1379 m) | PP482915 | PQ893172 | PQ894818 | PQ898913 | This study |
| *Hyloscirtus dispersus*  sp. nov. | QCAZ 69561 | Ecuador: Morona Santiago: Puchimi (2.7774°S, 78.1595°W, 1365 m) | PP482916 | PQ893173 | PQ894819 | PQ898914 | This study |
| *Hyloscirtus dispersus*  sp. nov. | QCAZ 69562 | Ecuador: Morona Santiago: Puchimi (2.7774°S, 78.1595°W, 1450 m) | PP482917 | - | PQ894820 | PQ898915 | This study |
| *Hyloscirtus dispersus*  sp. nov. | QCAZ 71029 | Ecuador: Morona Santiago: Cordillera del Cóndor, 2-3 Km SE of Kunkuk Shuar Community (3.3302°S, 78.1972°W, 1521 m) | PP482835 | PQ893093 | PQ894736 | PQ898854 | This study |
| *Hyloscirtus dispersus*  sp. nov. | QCAZ 71428 | Ecuador: Morona Santiago: Cordillera de Cutucú lowlands, Carlos Hurtado’s house surroundings (2.7818°S, 78.1604°W, 1380 m) | PP482836 | PQ893094 | PQ894737 | - | This study |
| *Hyloscirtus elbakyanae*  sp. nov. | QCAZ  53807* | Ecuador: Morona Santiago: Shaime Community near Mirador de la Virgen (2.9755°S, 77.8034°W, 622 m) | PP482865 | PQ893124 | PQ894768 | PQ898870 | This study |
| *Hyloscirtus elbakyanae*  sp. nov. | QCAZ 53808* | Ecuador: Morona Santiago: Shaime Community near Mirador de la Virgen (2.9755°S, 77.8034°W, 622 m) | PP482866 | PQ893125 | PQ894769 | PQ898871 | This study |
| *Hyloscirtus elbakyanae*  sp. nov. | QCAZ 72665 | Ecuador: Morona Santiago: Río Shaime surroundings, upper area (2.9409°S, 77.8012°W, 511 m) | PP482830 | - | - | - | This study |
| *Hyloscirtus elbakyanae*  sp. nov. | QCAZ 72667 | Ecuador: Morona Santiago: Tiwintza-Shaime road, bridge (2.9750°S, 77.7957°W, 214 m) | PP482831 | - | - | - | This study |
| *Hyloscirtus elbakyanae*  sp. nov. | QCAZ 72668 | Ecuador: Morona Santiago: Mirador de la Virgen, Tiwintza-Shaime road (2.9756°S, 77.8015°W, 529 m) | PP482832 | - | - | - | This study |
| *Hyloscirtus elbakyanae*  sp. nov. | QCAZ 72669 | Ecuador: Morona Santiago: Peñas-Shaime road, bridge (2.9663°S, 77.8468°W, 363 m) | PP482833 | - | - | - | This study |
| *Hyloscirtus jahni* | MHNLS 20318 | Venezuela: Mérida: Quebrada La Sucia, road Ejido-Jají | MG596776 | - | - | - | Rojas-Runjaic et al. 2018 |
| *Hyloscirtus jahni* | MHNLS 20324 | Venezuela: Mérida: road between Las Cruces and Miraflores | MG596779 | - | - | - | Rojas-Runjaic et al. 2018 |
| *Hyloscirtus japreria* | MHNLS 19235 | Venezuela: Zulia: Rio Negro basin, Guacharaca camp (10.0727°N, 72.8546° W, 1661 m) | MG596769 | - | - | - | Rojas-Runjaic et al. 2018 |
| *Hyloscirtus japreria* | UISA 5496 | Colombia: Guajira: El Manantialito (10.5877°N, 72.8185° W, 1754 m) | MG596770 | - | - | - | Rojas-Runjaic et al. 2018 |
| *Hyloscirtus larinopygion* | QCAZ 45462 | Ecuador: Carchi: Road Tulcán-Maldonado. Quebrada Centella (0.8179°N, , 78.0160°W, 2806 m) | JX155818 | - | - | - | Coloma et al. 2012 |
| *Hyloscirtus lascinius* | KU  181086 | Venezuela: Tachira: 11 km S Delicias | DQ380359 | - | - | - | Wiens et al. 2006 |
| *Hyloscirtus lascinius* | MHNLS 19163 | Venezuela: Zulia: Guacharaca camp (10.0727°N, 72.8546°W, 1661 m) | MG596762 | - | - | - | Rojas-Runjaic et al. 2018 |
| *Hyloscirtus lascinius* | MHNLS 19164 | Venezuela | MG596763 | - | - | - | Rojas-Runjaic et al. 2018 |
| *Hyloscirtus lindae* | QCAZ 45463 | Ecuador: Sucumbíos: near Santa Bárbara vía La Bonita (0.6159°N, 77.4879°W, 2341m) | JX155823 | - | - | - | Coloma et al. 2012 |
| *Hyloscirtus mashpi* | MZUTI  610 | Ecuador: Pichincha Reserva de Biodiversidad Mashpi (0.0680°N, 77.3970°W; 908 m) | KT279510 | - | - | - | Guayasamin et al. 2015 |
| *Hyloscirtus mashpi* | QCAZ  46000 | Ecuador: Imbabura: near Reserva Cotacachi-Cayapas (0.3310°N, 78.9315°W, 670 m) | PP482853 | PQ893111 | PQ894755 | - | This study |
| *Hyloscirtus maycu*  sp. nov. | QCAZ 67082 | Ecuador: Zamora Chinchipe. Reserva Natural Maycu (4,2287°S, 78,6160°W, 1183 m) | PP482906 | PQ893163 | PQ894809 | PQ898904 | This study |
| *Hyloscirtus maycu*  sp. nov. | QCAZ 67084 | Ecuador: Zamora Chinchipe: Reserva Natural Maycu (4,2287°S, 78,6160°W, 1183 m) | PP482907 | PQ893164 | PQ894810 | PQ898905 | This study |
| *Hyloscirtus maycu*  sp. nov. | QCAZ 67085 | Ecuador: Zamora Chinchipe: Reserva Natural Maycu (4,2216°S, 78,6452°W, 983 m) | PP482908 | PQ893165 | PQ894811 | PQ898906 | This study |
| *Hyloscirtus maycu*  sp. nov. | QCAZ 67086 | Ecuador: Zamora Chinchipe: Reserva Natural Maycu (4,2216°S, 78,6452°W, 983 m) | PP482909 | PQ893166 | PQ894812 | PQ898907 | This study |
| *Hyloscirtus maycu*  sp. nov. | QCAZ 67087 | Ecuador: Zamora Chinchipe: Reserva Natural Maycu (4,2216°S, 78,6452°W, 983 m) | PP482910 | PQ893167 | PQ894813 | PQ898908 | This study |
| *Hyloscirtus maycu*  sp. nov. | QCAZ 68055 | Ecuador: Zamora Chinchipe: Nuevo Paraíso, camp near Río Nangaritza (4,4442°S, 78,8134°W, 1127 m) | PP482911 | PQ893168 | PQ894814 | PQ898909 | This study |
| *Hyloscirtus pacha* | KU 202760 | Ecuador: Azuay: 2.0 km SSE Palmas (2340 m) | AY326057 | - | - | - | Darst and Cannatella 2004 |
| *Hyloscirtus palmeri* | MZUTI  608 | Ecuador: Pichincha: Mashpi Biodiversity Reserve (0.0680°N, 77.3970°W, 908 m) | KT279549 | - | - | - | Guayasamin et al. 2015 |
| *Hyloscirtus palmeri* | QCAZ  55526* | Ecuador: Esmeraldas: Durango (1.0427°N, 78.6244°W, 188 m) | PP482834 | PQ893092 | - | - | This study |
| *Hyloscirtus palmeri* | QCAZ 64850 | Ecuador: Carchi: Chical (1.0079°N, 78.2222°W, 998 m) | PP482827 | - | - | - | This study |
| *Hyloscirtus palmeri* | QCAZ 71776 | Ecuador: Pichincha: Milpe Bird Sanctuary (0.0348°N, -78.8662°W, 1062 m) | PP482828 | - | - | - | This study |
| *Hyloscirtus palmeri* | SIUCH  6924 | Panamá: El Cope: Omar Torrijos National Park | AY843650 | - | AY819324 |  | Faivovich et al. 2005 |
| *Hyloscirtus pantostictus* | QCAZ 45435 | Ecuador: Sucumbíos: near Santa Bárbara (0.6444°N, 77.5522°W, 2709 m) | JX155820 | - | - | - | Coloma et al. 2012 |
| *Hyloscirtus platydactylus* | MHNLS 20321 | Venezuela: Mérida: Quebrada La Sucia, road Ejido-Jají | MG596772 | - | - | - | Rojas-Runjaic et al. 2018 |
| *Hyloscirtus platydactylus* | MHNLS 20325 | Venezuela: Mérida: road between Las Cruces and Miraflores | MG596774 | - | - | - | Rojas-Runjaic et al. 2018 |
| *Hyloscirtus phyllognathus* | KU  212119 | Peru: San Martin: 17 km NE Tarapoto (850 m) | DQ380369 | - | - | - | Wiens et al. 2006 |
| *Hyloscirtus princecharlesi* | QCAZ 42165 | Ecuador: Imbabura: near Cuellaje, San Antonio (0.4775°N, 78.5626°W, 2720 m) | JX155806 | - | - | - | Coloma et al. 2012 |
| *Hyloscirtus psarolaimus* | QCAZ 46095 | Ecuador: Napo: 60 km E Salcedo (0.9709°S, 78.2413°W, 2748 m) | JX155809 | - | - | - | Coloma et al. 2012 |
| *Hyloscirtus ptychodactylus* | QCAZ 46030 | Ecuador: Cotopaxi: near Pilaló (0.9424°S, 78.9956°W, 2500 m) | JX155804 | - | - | - | Coloma et al. 2012 |
| *Hyloscirtus simmonsi* | KU  181167 | Colombia: Valle: Calima river, 1.5 km W Lago Calima | DQ380376 | AY819555 | AY819325 | - | Wiens et al. 2006 |
| *Hyloscirtus staufferorum* | QCAZ 45962* | Ecuador: Pastaza: Reserva Comunitaria Ankaku, Río Challuwa Yacu (1.2792°S, 78.0779°W, 2250 m) | JX155816 | - | - | - | Coloma et al. 2012 |
| *Hyloscirtus tapichalaca* | QCAZ 16704 | Ecuador: Zamora Chinchipe: Reserva Tapichalaca (4.4834°S, 79.1359°, 2697 m) | AY563625 | - | - | - | Faivovich et al. 2004 |
| *Hyloscirtus tigrinus* | QCAZ 41351 | Ecuador: Sucumbíos: Santa Bárbara, Quebrada Corazón (0.6437°N, 77.5321°W, 2638 m) | JX155810 | - | - | - | Coloma et al. 2012 |
| *Hyloscirtus* sp*.* | MZUTI  3474 | Ecuador: El Oro: Buenaventura Reserve (3.6646°S, 79.7478°W, 1073m) | KT279500 | - | - | - | Guayasamin et al. 2015 |
| *Hyloscirtus* sp. | QCAZ  63067 | Ecuador: El Oro: Buenaventura Reserve (3.6533°S, 79.7668°W, 578 m) | PP482899 | PQ893158 | PQ894802 | - | This study |
| *H.* UCS 1 | QCAZ 48199 | Ecuador: Morona Santiago: Indanza (3.1407°S, 78.4020°W, 700 m) | PP482857 | PQ893116 | PQ894760 | - | This study |
| *H*. UCS 2 | QCAZ  60025* (CORBIDI9976) | Perú: Picota: Puesto de Control 15, Quebrada Mishquillaquilla (6.94078°S, 76.06388°W, 959 m) | PP482894 | PQ893153 | PQ894797 | PQ898895 | This study |
| *Boana fasciata* | QCAZ 48583* | Ecuador: Zamora Chinchipe: Zamora (4.1013°S, 78.9623°W, 992 m) | JN970490 | - | - | - | Funk et al. 2012 |
| *Boana lanciformis* | QCAZ 20641* | Ecuador: Orellana: Yasuni National Park (0.6756°S, 76.3970°W, 250 m) | JN970512 | - | - | - | Funk et al. 2012 |
| *Dendropsophus reticulatus* | QCAZ 43085 | Ecuador: Sucumbios: Reserva Biológica Limoncocha (0.4062°S, 76.6194°W, 261 m) | KY406304 | KY406499 | - | KY406714 | Caminer et al. 2017 |
| *Dendropsophus triangulum* | QCAZ 44457 | Ecuador: Orellana: Rio Napo, Chiroisla (0.5756°S, 75.8998°W, 203 m) | KY406323 | KY406548 | - | KY406718 | Caminer et al. 2017 |
| *Agalychnis callidryas* | SAMN05559871* | - | - | - | - | - | Streicher et al. 2016  BioSample |
| *Boana lanciformis* | SAMN05559916* | - | - | - | - | - | Streicher et al. 2016  BioSample |
| *Ceratophrys cornuta* | SAMN05559887* | - | - | - | - | - | Streicher et al. 2016  BioSample |
| *Dendropsophus leali* | SAMN05559892* | - | - | - | - | - | Streicher et al. 2016  BioSample |
| *Hyla cinerea* | SAMN05559910* | - | - | - | - | - | Streicher et al. 2016  BioSample |
| *Lepidobatrachus laevis* | SAMN05559917* | - | - | - | - | - | Streicher et al. 2016  BioSample |
| *Litoria caerulia* | SAMN05559920* | - | - | - | - | - | Streicher et al. 2016  BioSample |
| *Phyllomedusa tomopterna* | SAMN05559926* | - | - | - | - | - | Streicher et al. 2016  BioSample |
| *Scinax catharinae* | SAMN05559930* | - | - | - | - | - | Streicher et al. 2016  BioSample |

**Table S2.** Details of the preferred partitions found with their best substitution models under Partition Finder and Model Find searches.

| **Partition Finder search** | |
| --- | --- |
| **Preferred partition** | **Substitution model** |
| (1) 12S and ND1 first and third codon positions and adjacent tRNAs | GTR+I+G |
| (2) ND1 second codon position, RAG-1 second and third codon position | HKY+I+G |
| (3) c-myc first and second codon position | JC+I |
| (4) c-myc third codon position | JC+G |
| (5) RAG-1 first codon position | K80+G |
| **Model Finder search** | |
| **Preferred partition** | **Substitution model** |
| (1) 12S, ND1 first and third codon position and adjacent tRNAs | GTR+F+I+G4 |
| (2) ND1 second codon position | HKY+F+I+G4 |
| (3) c-myc first and second codon position | K2P+I |
| (4) c-myc third codon position | K2P+G4 |
| (5) RAG-1 first codon position | TN+F+I+G4 |
| (6) RAG-1 second codon position | TN+F |
| (7) RAG-1 third codon position | K3P+I |

**Table S3. Sequences for complete gene 16S, obtained from GenBank and amplified in this study.** See Table S1 for locality data.

| **Museum Number** | **Species** | **GenBank Accession Number** |
| --- | --- | --- |
| QCAZ 59815 | *H. albopunctulatus* | PV158113 |
| QCAZ 53808 | *H. elbakyanae* sp. nov. | PV158112 |
| QCAZ 48199 | *H*. UCS 1 | PV158111 |
| QCAZ 60025 | *H*. UCS 2 | PV158109 |
| MZUTI 2383 | *H. dispersus* sp. nov. | KT279545 |
| MZUTI 2384 | *H. dispersus* sp. nov. | KT279546 |
| MZUTI 2385 | *H. dispersus* sp. nov. | KT279547 |
| MZUTI 2386 | *H. dispersus* sp. nov. | KT279548 |
| QCAZ 23938 | *H. dispersus* sp. nov. | PV158106 |
| QCAZ 32271 | *H. dispersus* sp. nov. | PV158108 |
| QCAZ 41032 | *H. dispersus* sp. nov. | PV158107 |
| QCAZ 59820 | *H. dispersus* sp. nov. | PV158105 |
| QCAZ67087 | *H. maycu* sp. nov. | PV158110 |

**Table S4. Examined specimens for the morphological analysis include the voucher number, sex of the specimen and the hosting museum.** Abbreviations are: NHM: Natural History Museum London, QCAZ: Museo de Zoología QCAZ, CORBIDI: Centro de Ornitología y Biodiversidad.

| **Species** | **Voucher number** | **Sex** | **Museum** |
| --- | --- | --- | --- |
| *H. albopunctulatus* type | NHM159 | Male | NHM |
| *H. albopunctulatus* type | NHM160 | Male | NHM |
| *H. albopunctulatus* type | NHM161 | Male | NHM |
| *H. albopunctulatus* type | NHM162 | Male | NHM |
| *H. albopunctulatus* | QCAZ54111 | Male | QCAZ |
| *H. albopunctulatus* | QCAZ46447 | Male | QCAZ |
| *H. albopunctulatus* | QCAZ48254 | Male | QCAZ |
| *H. albopunctulatus* | QCAZ48503 | Male | QCAZ |
| *H. albopunctulatus* | QCAZ59810 | Male | QCAZ |
| *H. albopunctulatus* | QCAZ59813 | Male | QCAZ |
| *H. albopunctulatus* | QCAZ59814 | Male | QCAZ |
| *H. albopunctulatus* | QCAZ59815 | Male | QCAZ |
| *H. albopunctulatus* | QCAZ59816 | Male | QCAZ |
| *H. albopunctulatus* | QCAZ59817 | Male | QCAZ |
| *H. albopunctulatus* | QCAZ59822 | Male | QCAZ |
| *H. albopunctulatus* | QCAZ59823 | Male | QCAZ |
| *H. albopunctulatus* | QCAZ59827 | Male | QCAZ |
| *H. albopunctulatus* | QCAZ62188 | Female | QCAZ |
| *H. albopunctulatus* | QCAZ59825 | Female | QCAZ |
| *H. albopunctulatus* | QCAZ59826 | Female | QCAZ |
| *H. albopunctulatus* | QCAZ59828 | Female | QCAZ |
| *H. maycu* sp. nov. | QCAZ67081 | Female | QCAZ |
| *H. maycu* sp. nov. | QCAZ67082 | Male | QCAZ |
| *H. maycu* sp. nov. | QCAZ67087 | Male | QCAZ |
| *H. maycu* sp. nov. | QCAZ68055 | Male | QCAZ |
| *H. maycu* sp. nov*.* | QCAZ67086 | Female | QCAZ |
| *H. elbakyanae* sp. nov. | QCAZ53807 | Male | QCAZ |
| *H. elbakyanae* sp. nov. | QCAZ53808 | Male | QCAZ |
| *H. elbakyanae* sp. nov. | QCAZ53831 | Male | QCAZ |
| *H. elbakyanae* sp. nov. | QCAZ72667 | Male | QCAZ |
| *H. elbakyanae* sp. nov. | QCAZ72666 | Male | QCAZ |
| *H. elbakyanae* sp. nov. | QCAZ72669 | Male | QCAZ |
| *H. elbakyanae* sp. nov. | QCAZ72668 | Male | QCAZ |
| *H. elbakyanae* sp. nov. | QCAZ72665 | Male | QCAZ |
| *H. elbakyanae* sp. nov. | QCAZ73709 | Male | QCAZ |
| *H. dispersus* sp. nov. | QCAZ57664 | Male | QCAZ |
| *H. dispersus* sp. nov. | QCAZ57667 | Male | QCAZ |
| *H. dispersus* sp. nov. | QCAZ18275 | Male | QCAZ |
| *H. dispersus* sp. nov. | QCAZ23937 | Male | QCAZ |
| *H. dispersus* sp. nov. | QCAZ23938 | Male | QCAZ |
| *H. dispersus* sp. nov. | QCAZ23945 | Male | QCAZ |
| *H. dispersus* sp. nov. | QCAZ32267 | Male | QCAZ |
| *H. dispersus* sp. nov. | QCAZ32271 | Male | QCAZ |
| *H. dispersus* sp. nov. | QCAZ40978 | Male | QCAZ |
| *H. dispersus* sp. nov. | QCAZ41554 | Male | QCAZ |
| *H. dispersus* sp. nov. | QCAZ42002 | Male | QCAZ |
| *H. dispersus* sp. nov. | QCAZ42047 | Male | QCAZ |
| *H. dispersus* sp. nov. | QCAZ46297 | Male | QCAZ |
| *H. dispersus* sp. nov. | QCAZ52006 | Male | QCAZ |
| *H. dispersus* sp. nov. | QCAZ52007 | Male | QCAZ |
| *H. dispersus* sp. nov. | QCAZ52458 | Male | QCAZ |
| *H. dispersus* sp. nov. | QCAZ52462 | Male | QCAZ |
| *H. dispersus* sp. nov. | QCAZ57015 | Male | QCAZ |
| *H. dispersus* sp. nov. | QCAZ57016 | Male | QCAZ |
| *H. dispersus* sp. nov. | QCAZ58735 | Male | QCAZ |
| *H. dispersus* sp. nov. | QCAZ59099 | Male | QCAZ |
| *H. dispersus* sp. nov. | QCAZ59820 | Male | QCAZ |
| *H. dispersus* sp. nov. | QCAZ59821 | Male | QCAZ |
| *H. dispersus* sp. nov. | QCAZ60688 | Male | QCAZ |
| *H. dispersus* sp. nov. | QCAZ60692 | Male | QCAZ |
| *H. dispersus* sp. nov. | QCAZ63488 | Male | QCAZ |
| *H. dispersus* sp. nov. | QCAZ66050 | Male | QCAZ |
| *H. dispersus* sp. nov. | QCAZ66051 | Male | QCAZ |
| *H. dispersus* sp. nov. | QCAZ66052 | Male | QCAZ |
| *H. dispersus* sp. nov. | QCAZ66709 | Male | QCAZ |
| *H. dispersus* sp. nov. | QCAZ66710 | Male | QCAZ |
| *H. dispersus* sp. nov. | QCAZ69548 | Male | QCAZ |
| *H. dispersus* sp. nov. | QCAZ69550 | Male | QCAZ |
| *H. dispersus* sp. nov. | QCAZ69555 | Male | QCAZ |
| *H. dispersus* sp. nov. | QCAZ69561 | Male | QCAZ |
| *H. dispersus* sp. nov. | QCAZ69562 | Male | QCAZ |
| *H. dispersus* sp. nov. | QCAZ57099 | Female | QCAZ |
| *H. dispersus* sp. nov. | QCAZ57100 | Female | QCAZ |
| *H. dispersus* sp. nov. | QCAZ57665 | Female | QCAZ |
| *H. dispersus* sp. nov. | QCAZ57666 | Female | QCAZ |
| *H. dispersus* sp. nov. | QCAZ41031 | Female | QCAZ |
| *H. dispersus* sp. nov. | QCAZ41649 | Female | QCAZ |
| *H. dispersus* sp. nov. | QCAZ41901 | Female | QCAZ |
| *H. dispersus* sp. nov. | QCAZ59099 | Female | QCAZ |
| *H. dispersus* sp. nov. | QCAZ60694 | Female | QCAZ |
| *H. dispersus* sp. nov. | QCAZ60695 | Female | QCAZ |
| *H. dispersus* sp. nov. | QCAZ68056 | Female | QCAZ |
| *H. phyllognathus* | CORBIDI16984 | Male | CORBIDI |
| *H. phyllognathus* | CORBIDI16985 | Male | CORBIDI |
| *H. phyllognathus* | CORBIDI16986 | Male | CORBIDI |
| *H. phyllognathus* | CORBIDI16987 | Male | CORBIDI |
| *H. phyllognathus* | CORBIDI16988 | Male | CORBIDI |
| *H. phyllognathus* | CORBIDI16994 | Male | CORBIDI |
| *H. phyllognathus* | CORBIDI16995 | Male | CORBIDI |
| *H. phyllognathus* | CORBIDI9590 | Male | CORBIDI |
| *H.* UCS 2 | CORBIDI9976 | Male | CORBIDI |

**Table S5**. **Measurements (in mm) of specimens of *Hyloscirtus.*** *Hyloscirtus albopunctulatus* (Lineage A), *H. maycu* sp. nov. (Lineage B), *H. elbakyanae* sp. nov. (Lineage D), *H. dispersus* sp. nov. (Lineage G), *H. phyllognathus* sensu stricto (Lineage E) and the *H.* UCS 2 (Lineage F). Mean ± SD is given with range in parentheses. Abbreviations are: SVL = Snout-vent length; HL = Head length; HW = Head width; ED = Eye diameter; TD = Tympanum diameter; TL = Tibia length; FeL = Femur length; FL = Foot length; IoD = Interorbital distance; InD = Internarial distance. Numbers in parentheses represent the number of individuals analyzed.

| **Measure** | ***H. albopunctulatus*** | | ***H. maycu* sp. nov.** | | ***H. elbakyanae* sp. nov.** | ***H. dispersus* sp. nov.** | | ***H. phyllognathus*** | **UCS 2** |
| --- | --- | --- | --- | --- | --- | --- | --- | --- | --- |
|  | **Males  (17)** | **Females (4)** | **Males (3)** | **Females (2)** | **Males (9)** | **Males (36)** | **Females (11)** | **Males (8)** | **Male**  **(1)** |
| **SVL** | 32.2±1.4 | 37.8±0.4 | 33.4±1.3 | 42.7±1.4 | 36.3±1.2 | 34.1±1.8 | 41.3±2.6 | 36.9±1.5 | 37.9 |
|  | (30.3–35.5) | (37.1–38.1) | (31.9–34.2) | (41.7–43.7) | (34.5–37.6) | (31.3–38.7) | (35.4–45.2) | (35.2–38.9) |  |
| **FL** | 12.94±0.58 | 13.3±0.66 | 13.30±0.66 | 16.3±0.4 | 14.20±0.38 | 14.31±0.86 | 16.88±1.37 | 14.55±0.81 | 15.3 |
|  | (12.25–14.07) | (12.62–13.95) | (12.62–13.95) | (16.1–16.5) | (13.59–14.74) | (12.69–15.87) | (15.09–18.51) | (13.48–15.74) |  |
| **ED** | 2.95±0.20 | 3.04±0.04 | 3.04±0.04 | 3.96±0.16 | 3.55±0.27 | 3.24±0.23 | 3.79±0.46 | 3.82±0.24 | 3.5 |
|  | (2.69–3.24) | (3–3.08) | (3–3.08) | (3.88–4.04) | (3.12–3.92) | (2.73–3.82) | (3.21–4.58) | (3.48–4.09) |  |
| **TD** | 1.14±0.35 | 1.52±0.46 | 1.52±0.46 | 1.81±0.37 | 1.6±0.18 | 1.17±0.26 | 1.49±0.27 | 1.81±0.13 | 1.8 |
|  | (0.64–1.8) | (1.09–2.0) | (1.09–2) | (1.62–1.99) | (1.44–1.99) | (0.72–1.63) | (1.07–1.88) | (1.58–1.99) |  |
| **HW** | 10.15±0.52 | 10.33±0.66 | 10.33±0.66 | 12.53±0.36 | 11.00±0.45 | 10.29±0.81 | 12.35±1.07 | 14.63±0.79 | 12.7 |
|  | (9.1–11.06) | (9.77–11.07) | (9.77–11.07) | (12.35–12.71) | (10.54–11.73) | (9.05–12.73) | (9.95–14.39) | (13.48–15.74) |  |
| **HL** | 9.75±0.53 | 9.61±0.37 | 9.61±0.37 | 11.87±1.3 | 11.05±0.67 | 10.69±0.70 | 12.07±0.94 | 11.26±0.41 | 10.8 |
|  | (8.7–10.52) | (9.21–9.93) | (9.21–9.93) | (11.22–12.52) | (10.05–11.93) | (9.32–12.33) | (10.65–13.95) | (10.78–11.73) |  |
| **TL** | 15.10±0.88 | 16.37±0.77 | 16.37±0.77 | 19.23±1.96 | 17.49±0.60 | 16.91±0.81 | 20.16±1.23 | 18.65±0.76 | 18.7 |
|  | (13.87–16.79) | (15.62–17.16) | (15.62–17.16) | (18.25–20.21) | (16.7–18.13) | (15.2–18.64) | (18.21–22.05) | (18.18–20.48) |  |
| **FeL** | 14.41±0.93 | 15.65±1.51 | 15.65±1.51 | 19.56±1.27 | 16.97±0.52 | 15.56±0.97 | 19.06±1.49 | 17.60±0.90 | 17.7 |
|  | (12.39–15.37) | (14.15–17.17) | (14.15–17.17) | (18.92–20.19) | (16.36–17.78) | (13.19–17.56) | (16.98–21.48) | (16.74–19.57) |  |
| **IoD** | 4.58±0.40 | 4.81±0.09 | 4.81±0.09 | 5.32±0.45 | 5.37±0.23 | 4.82±0.43 | 5.43±0.36 | 3.97±0.35 | 4.1 |
|  | (4.04–5.22) | (4.76–4.92) | (4.76–4.92) | (5.09–5.54) | (5.1–5.76) | (4.17–5.86) | (4.65–5.83) | (3.57–4.74) |  |
| **InD** | 2.82±0.26 | 3.04±0.33 | 3.04±0.33 | 3.79±0.18 | 3.09±0.29 | 3.06±0.22 | 3.56±0.33 | 2.98±0.17 | 3.1 |
|  | (2.38–3.28) | (2.68–3.34) | (2.68–3.34) | (3.7–3.88) | (2.62–3.49) | (2.68–3.5) | (2.98–4.1) | (2.74–3.21) |  |

**Table S6. Character loadings and eigenvalues for Principal Components (PC I, II and III) of the morphology analysis.** The analysis was based on seven size-corrected morphometric variables of adult males and females of *Hyloscirtus albopunctulatus* (Lineage A), *H. maycu* sp. nov. (Lineage B), *H. elbakyanae* sp. nov. (Lineage D; only males), *H. dispersus* sp. nov. (Lineage G), H. phyllognathus sensu stricto (Lineage E) and one male of the *H.* UCS 2 (Lineage F). Morphometric variables of *H. albopunctulatus* type material were included in the analysis. Bold numbers indicate the highest loadings.

| **Morphometric Variables** | **PC I** | **PC II** | **PCIII** |
| --- | --- | --- | --- |
| Foot length | 0.05 | **-0.48** | 0.33 |
| Eye diameter | **0.57** | -0.12 | -0.25 |
| Head width | **-0.46** | 0.26 | 0.31 |
| Head length | -0.45 | -0.38 | -0.37 |
| Femur length | -0.41 | -0.08 | **0.57** |
| Interorbital distance | 0.26 | -0.45 | 0.29 |
| Internarial distance | 0.01 | **-0.56** | **0.41** |
| Eigenvalue | 1.74 | 1.61 | 1.11 |
| % of variation | 24.4 | 23.0 | 15.9 |

**Table S7. Character loadings and eigenvalues for Principal Components (PC I–II) of the acoustic analysis.** Six acoustic variables of adult of *Hyloscirtus albopunctulatus* (Lineage A), *H. maycu* sp. nov. (Lineage B), *H. elbakyanae* sp. nov. (Lineage D), *H. phyllognathus* sensu stricto (Lineage E), *H.* UCS 2 (Lineage F), *H. dispersus* sp. nov. (Lineage G) and *H. torrenticola* sensu stricto were analyzed. Bold numbers indicate the highest loadings.

| **Acoustic variables** | **PC I** | **PC II** |
| --- | --- | --- |
| Call duration | **0.47** | 0.12 |
| Rise time of the call | -0.22 | **0.64** |
| Inter-call interval | 0.29 | **0.61** |
| Dominant frequency of the call | **0.48** | -0.18 |
| Fundamental frequency of the call | **0.51** | -0.27 |
| Bandwidth frequency of the call | -0.40 | -0.32 |
| Eigenvalue | 3.19 | 1.52 |
| % of variation | 53.1 | 25.3 |

**Table S8. Character loadings and eigenvalues for Principal Components (PC I–II) of the environmental analysis.** The analysis was based on eight environmental variables of 67 populations of *Hyloscirtus albopunctulatus* (Lineage A), *H. maycu* sp. nov. (Lineage B), *H.* UCS 1 (Lineage C), *H. elbakyanae* sp. nov. (Lineage D), *H. phyllognathus* sensu stricto (Lineage E), *H.* UCS 2 (Lineage F), *H. dispersus* sp. nov. (Lineage G), and *H. torrenticola* sensu stricto. Bold numbers indicate the highest loadings.

| **Environmental variables** | **PC I** | **PC II** |
| --- | --- | --- |
| Annual mean temperature – BIO1 | -0.33 | **0.40** |
| Mean temperature diurnal range (Mean of monthly (max temp – min temp)) – BIO2 | 0.12 | **0.49** |
| Temperature seasonality – BIO4 | **-0.40** | 0.007 |
| Maximum temperature of warmest month – BIO5 | -0.28 | **0.44** |
| Minimum temperature of the coldest month – BIO6 | -0.37 | 0.35 |
| Annual precipitation – BIO12 | **-0.41** | -0.28 |
| Precipitation of warmest quarter – BIO18 | **-0.45** | -0.18 |
| Precipitation of coldest quarter – BIO19 | -0.33 | -0.39 |
| Eigenvalue | 3.91 | 3.43 |
| % of variation | 48.9 | 42.9 |

**Table S9.** **Additional specimens examined.**

| **Museum Voucher** | **Species** | **Locality** |
| --- | --- | --- |
| QCAZ 53831 | *H. elbakyanae* sp. nov. | Ecuador: Morona Santiago: Comunidad Shaime, cerca al Mirador de la Virgen (2.9755°S, 77.8034°W, 622 m) |
| QCAZ 72666 | *H. elbakyanae* sp. nov. | Ecuador: Morona Santiago: near Río Shaime (2.9416°S, -77.8010°W, 448 m) |
| QCAZ 73709 | *H. elbakyanae* sp. nov. | Ecuador: Morona Santiago: Peñas-Shaime (2.9645°S, 77.8448°W, 211 m) |
| QCAZ 40878 | *H. dispersus* sp. nov. | Ecuador: Pastaza: Reserva Zanja Sancha Arajuno (1.353299°S, 77.86450°W, 940 m) |
| QCAZ 49032 | *H. dispersus* sp. nov. | Ecuador: Morona Santiago: Bosque Protector Abanico (2.244842°S, 78.20528°W, 1646 m) |
| QCAZ 52463 | *H. dispersus* sp. nov. | Ecuador: Tungurahua: Reserva Río Zuñac (1.3755°S, 78.15360°W, 1597 m) |
| QCAZ 57014 | *H. dispersus* sp. nov. | Ecuador: Morona Santiago: near to 9 de Octubre (2.2350°S, 78.2164°W, 1675 m) |
| QCAZ 58733 | *H. dispersus* sp. nov. | Ecuador: Morona Santiago: Sardinayacu, Sangay National Park (2.0928°S, 78.1687°W, 1475 m) |
| QCAZ 69546 | *H. dispersus* sp. nov. | Ecuador: Morona Santiago: Puchimi (2,7828°S, 78,1602°W, 1421 m) |
| QCAZ 69563 | *H. dispersus* sp. nov. | Ecuador: Morona Santiago: Puchimi (2,7828°S, 78,1602°W, 1421 m) |
| QCAZ 59824 | *H. albopunctulatus* | Ecuador: Pastaza: Zarentza Community, Llanganates National Park (1.3397°S, 78.0594°W, 1360 m) |
| QCAZ 59811 | *H. albopunctulatus* | Ecuador: Pastaza: Zarentza Community, Llanganates National Park (1.3543°S, 78.0620°W, 1388 m) |
| QCAZ 59808 | *H. albopunctulatus* | Ecuador: Pastaza: Zarentza Community, Llanganates National Park (1.3564°S,78.0580°W, 1367 m) |
| QCAZ 67081 | *H. maycu* sp. nov. | Ecuador: Zamora Chinchipe. Reserva Natural Maycu (4,2287°S, 78,6160°W, 1183 m) |
| QCAZ 67083 | *H. maycu* sp. nov. | Ecuador: Zamora Chinchipe. Reserva Natural Maycu (4,2287°S, 78,6160°W, 1183 m) |
| CORBIDI 16984 | *H. phyllognathus* | Perú: San Martín: La Banda de Chiclayo: Km 35, carreterra Tarapoto-Yurimaguas  (6.4294°S, 76.2676°W, 594 m). |
| CORBIDI 16985 | *H. phyllognathus* | Perú: San Martín: La Banda de Chiclayo: Km 35, carreterra Tarapoto-Yurimaguas  (6.4294°S, 76.2676°W, 594 m). |
| CORBIDI 16986 | *H. phyllognathus* | Perú: San Martín: La Banda de Chiclayo: Km 35, carreterra Tarapoto-Yurimaguas  (6.4294°S, 76.2676°W, 594 m). |
| CORBIDI 16987 | *H. phyllognathus* | Perú: San Martín: La Banda de Chiclayo: Km 35, carreterra Tarapoto-Yurimaguas  (6.4294°S, 76.2676°W, 594 m). |
| CORBIDI 16988 | *H. phyllognathus* | Perú: San Martín: La Banda de Chiclayo: Km 35, carreterra Tarapoto-Yurimaguas  (6.4294°S, 76.2676°W, 594 m). |
| CORBIDI 16994 | *H. phyllognathus* | Perú: San Martín: La Banda de Chiclayo: Km 35, carreterra Tarapoto-Yurimaguas  (6.4294°S, 76.2676°W, 594 m). |
| CORBIDI 16995 | *H. phyllognathus* | Perú: San Martín: La Banda de Chiclayo: Km 35, carreterra Tarapoto-Yurimaguas  (6.4294°S, 76.2676°W, 594 m). |
| CORBIDI 9590 | *H. phyllognathus* | Perú: San Martín: La Banda de Chiclayo: Catarata Ahuashiyacu (6.5000°S, 76.3333°W, 730 m). |

**Supplementary Figures**

**Figure S1. Map showing the current distribution range known for *H. albopunctulatus, H. phyllognathus* and *H. torrenticola* before this study.** The distribution polygons were downloaded from the International Union for Conservation of Nature (IUCN, 2024) website.

**
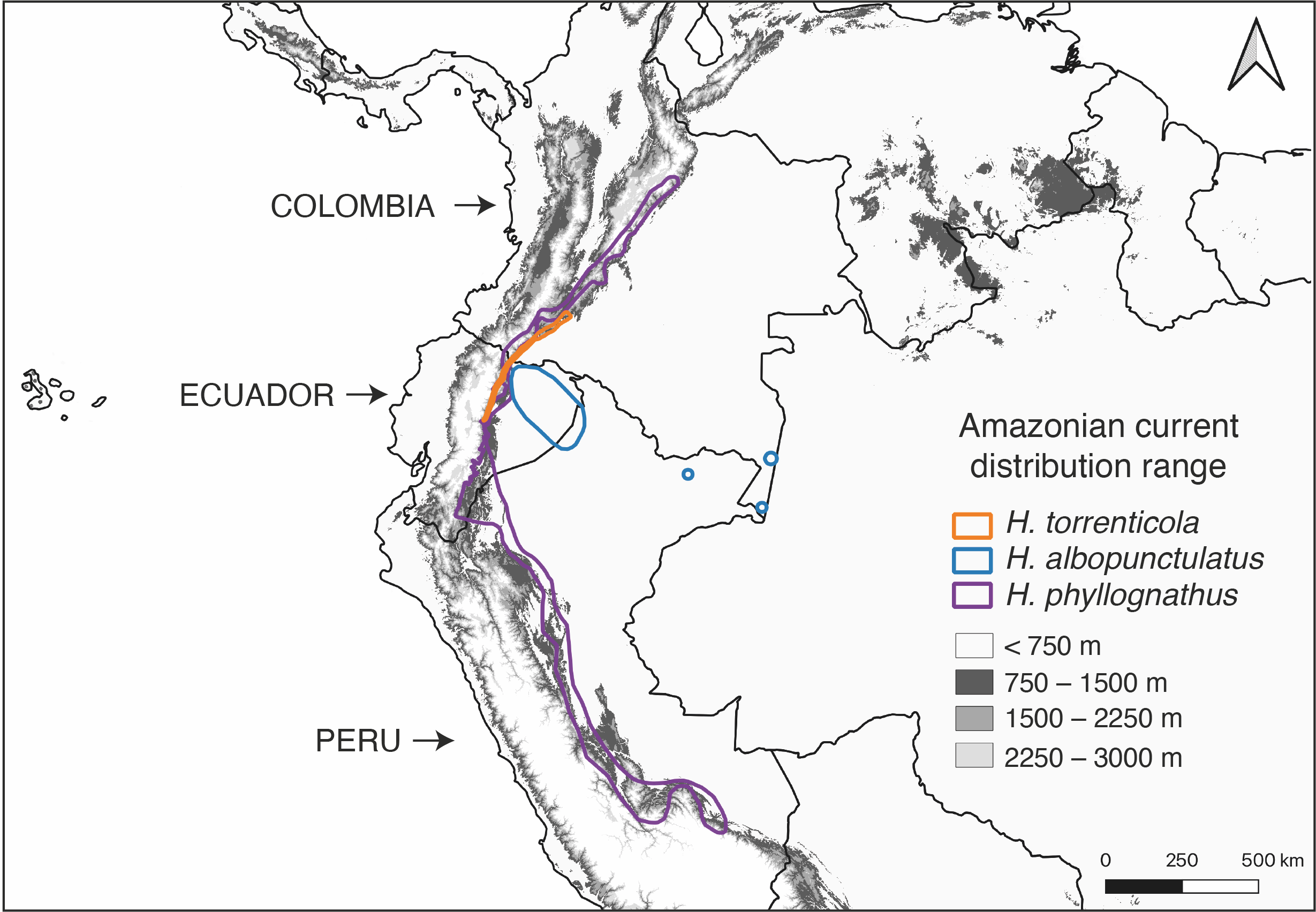
**

**Figure S2.** Consensus tree obtained under Maximum likelihood criterion when using taxa with more than 300 UCE loci enriched. Museum number of each individual is indicated. Numbers over the branches represent bootstrap values. Missing values indicate branch’s support below 60. All samples within the *H. bogotensis* group belong to Ecuador except for QCAZ 60025 from Peru (Lineage F).


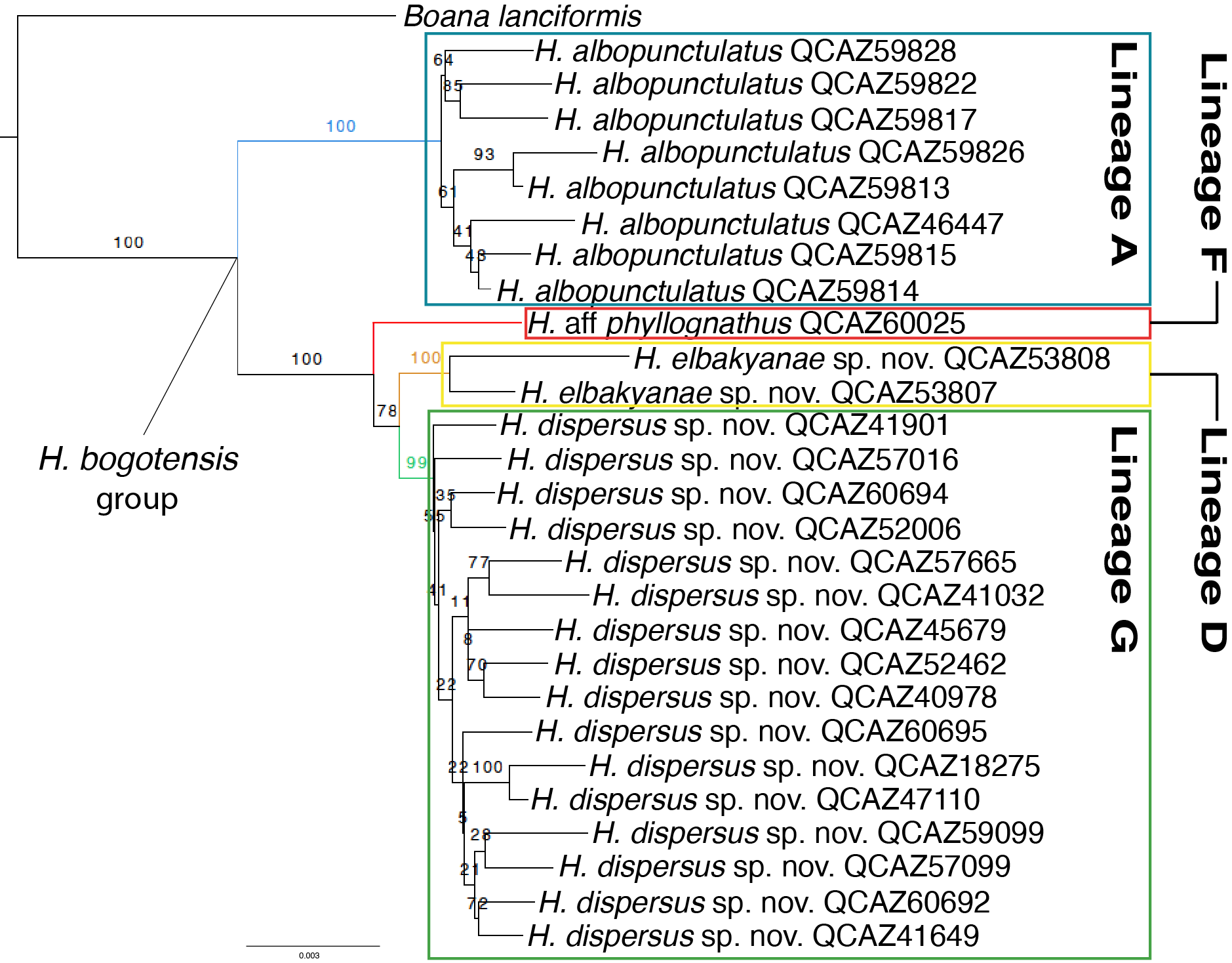


**Figure S3.** Consensus tree obtained under Maximum likelihood criterion when using taxa with more than 200 UCE loci enriched. Museum number of each individual is indicated. Numbers over the branches represent bootstrap values. Missing values indicate branch’s support below 60. Red branches were removed from the phylogeny shown in Figure 3 because of incorrect placement. All samples within the *H. bogotensis* group belong to Ecuador except for QCAZ 60025 (Lineage F) from Perú.


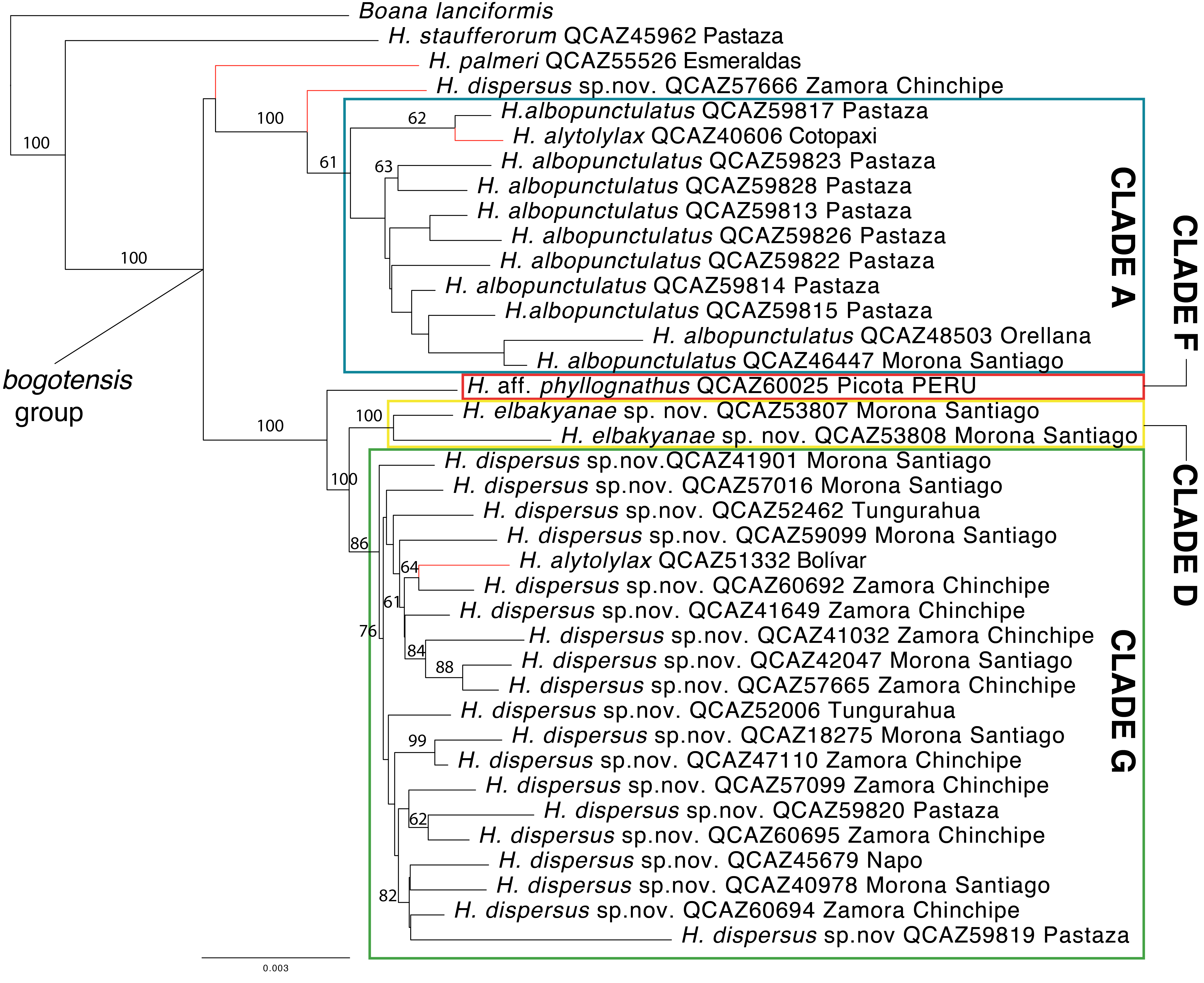


**Figure S4.** Consensus tree obtained under Maximum likelihood criterion when using taxa with more than 300 UCE loci enriched. Museum number of each individual is indicated. Numbers over the branches represent bootstrap values. Missing values indicate branch’s support below 60. Red branches were removed from the phylogeny shown in Figure S3 because of incorrect placement. All samples within the *H. bogotensis* group belong to Ecuador except for QCAZ 60025 (Lineage F) from Perú.


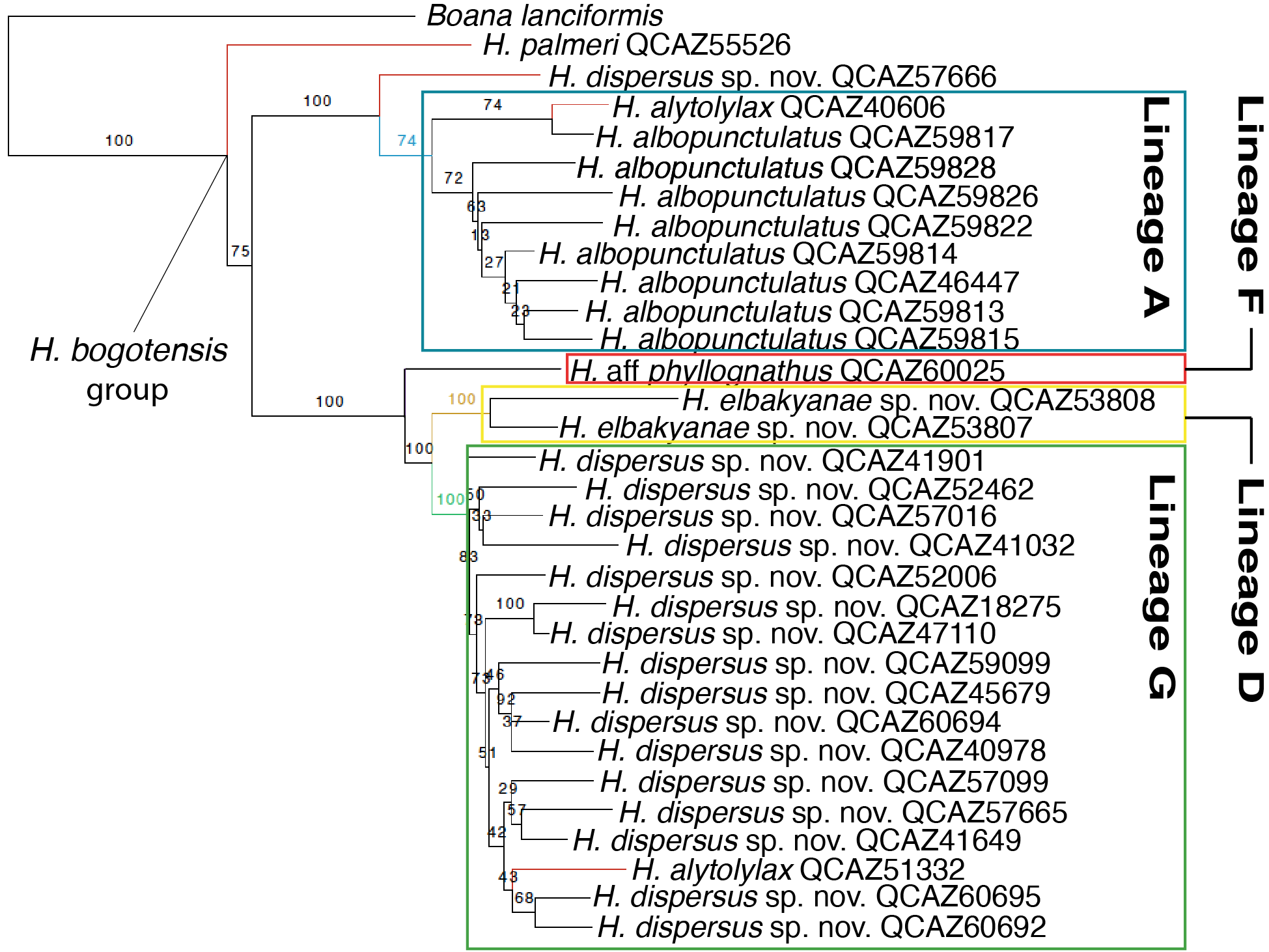


**Figure S5.** S**pecies delimitation tree based on the Poisson Tree Process (PTP) criteria, using the 12S mitochondrial gene.** The blue lines indicate branching processes among species, while red lines indicate taxa that should be considered as part of the same lineage.

**
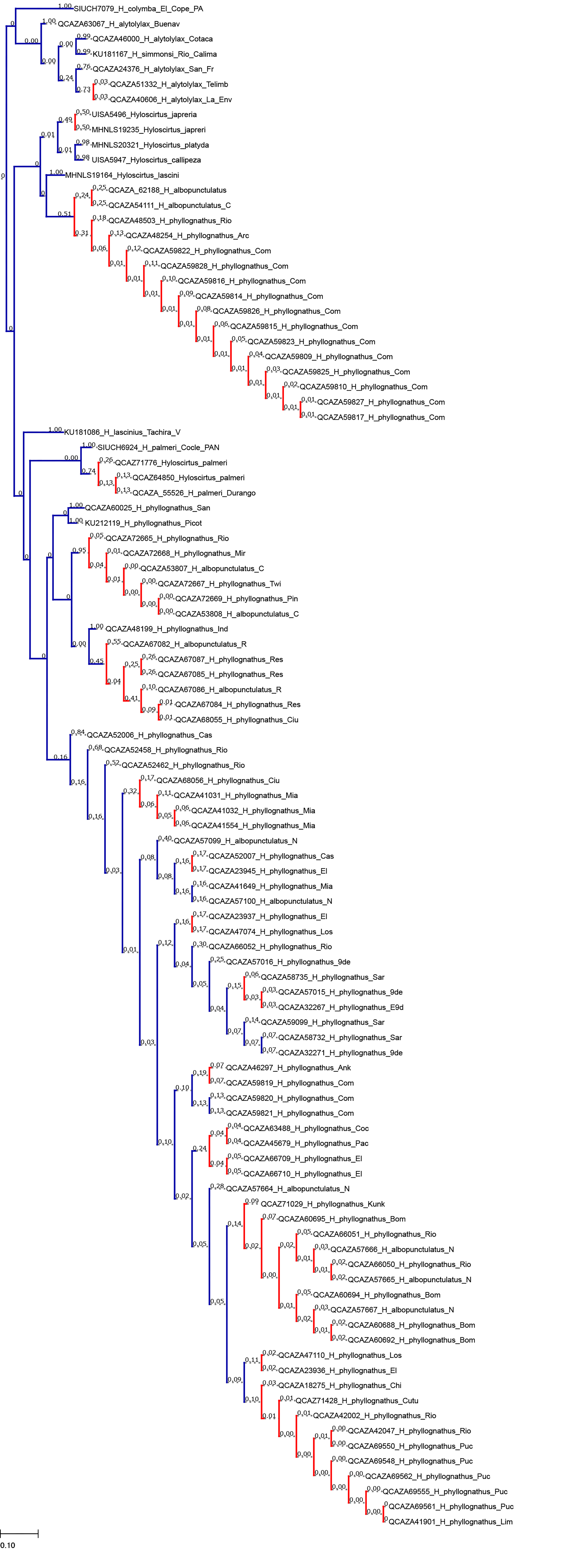
**

**Figure S6.** **Plot depicting the number of species recovered by the ABGD criteria as a function of the prior for intraspecific genetic divergences.**

**
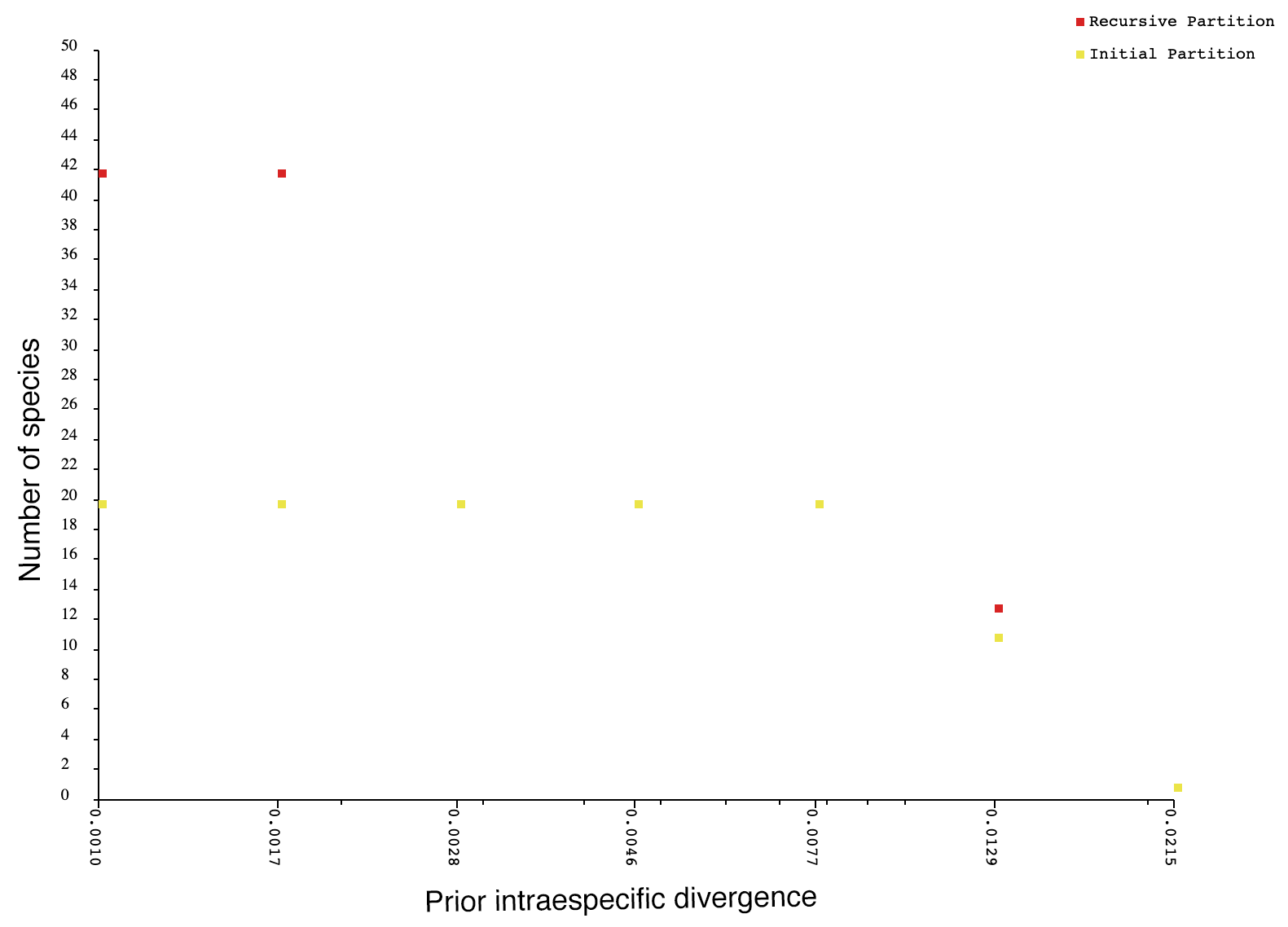
**

**Figure S7. Haplotype network for DNA sequences of 59 individuals for the nuclear gene C-myc.** Haplotype I is shown with semitransparent color to visualize alternative links (dashed lines).

**
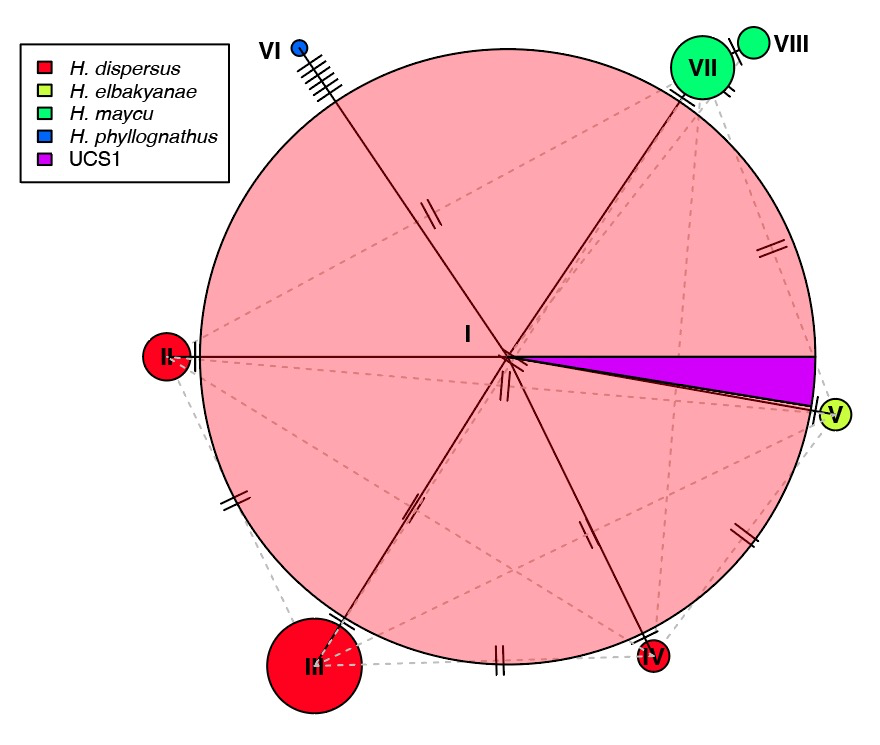
**
